# A rhythmically pulsing leaf-spring DNA-origami nanoengine that drives a passive follower

**DOI:** 10.1038/s41565-023-01516-x

**Published:** 2023-10-19

**Authors:** Mathias Centola, Erik Poppleton, Sujay Ray, Martin Centola, Robb Welty, Julián Valero, Nils G. Walter, Petr Šulc, Michael Famulok

**Affiliations:** 1 LIMES Program Unit Chemical Biology & Medicinal Chemistry, c/o Kekulé Institut für Organische Chemie und Biochemie, Universität Bonn, Bonn, Germany; 2 Max-Planck Institute for Neurobiology of Behaviour, Bonn, Germany; 3 School of Molecular Sciences and Center for Molecular Design and Biomimetics, The Biodesign Institute, Arizona State University, Tempe, AZ USA; 4 Single Molecule Analysis Group, Department of Chemistry, Ann Arbor, MI USA; 5 Max-Planck-Institute of Biophysics, Frankfurt, Germany; 6 Present Address: Max-Planck-Institute for Medical Research, Heidelberg, Germany; 7 Present Address: Interdisciplinary Nanoscience Center – INANO-MBG, iNANO-huset, Århus, Denmark

**Keywords:** DNA nanomachines, Molecular machines and motors

## Abstract

Molecular engineering seeks to create functional entities for modular use in the bottom-up design of nanoassemblies that can perform complex tasks. Such systems require fuel-consuming nanomotors that can actively drive downstream passive followers. Most artificial molecular motors are driven by Brownian motion, in which, with few exceptions, the generated forces are non-directed and insufficient for efficient transfer to passive second-level components. Consequently, efficient chemical-fuel-driven nanoscale driver–follower systems have not yet been realized. Here we present a DNA nanomachine (70 nm × 70 nm × 12 nm) driven by the chemical energy of DNA-templated RNA-transcription-consuming nucleoside triphosphates as fuel to generate a rhythmic pulsating motion of two rigid DNA-origami arms. Furthermore, we demonstrate actuation control and the simple coupling of the active nanomachine with a passive follower, to which it then transmits its motion, forming a true driver–follower pair.

## Abstract (web summary)

An autonomous DNA-origami nanomachine powered by the chemical energy of DNA-templated RNA-transcription-consuming nucleoside triphosphates as fuel performs rhythmic pulsations is demonstrated. In combination with a passive follower, the nanomachine acts as a mechanical driver with molecular precision.

## Main

Active mechanical motion of nanoscale objects is paramount for the bottom-up construction of bio- or technomimetic nanomechanical machines^1–4^ that can perform tasks such as pumping^5^, walking^6^, transduction or sensing of molecules or signals^7,8^, transport^9,10^ or any process involving motion^11,12^. Both in the nano- and the macroscopic worlds these processes require fuel-powered engines that perform periodically repeating motion. Impressive examples of synthetic pumping, rotating or moving fuel-driven nanodevices exist^13–18^, and even an electric-field-driven Brownian motion ratchet rotor capable of torque transmission was recently realized^17^. In contrast, the creation of engines that generate active rhythmic or rotating motion at the nanoscale, driven by chemical fuel, remains challenging^19,20^. Here we report a biohybrid nanoengine that pulses rhythmically, driven by a covalently bound T7 RNA polymerase (T7RNAP) that consumes nucleoside triphosphates (NTPs) as fuel to build up potential energy, which is stored as spring-tension in a compliant flexure mechanism, followed by active relaxation. We have previously presented a biohybrid DNA nanomachine consisting of a stator with a non-covalently bound T7RNAP that unidirectionally rotates a catenated DNA wheel^21^. The nanoengine introduced here represents an advance over the previous system in that the generated pulsating motion can be directly transferred to a passive downstream structure. The engine thus acts as a mechanical driver that can actuate a passive follower, opening opportunities for its future use in driving more complex nanomachines, similar to the balance wheel in a watch or in Leonardo da Vinci’s self-propelled cart.

## Functional characterization of the nanoengine supports pulsing motion

**Fig. 1:**
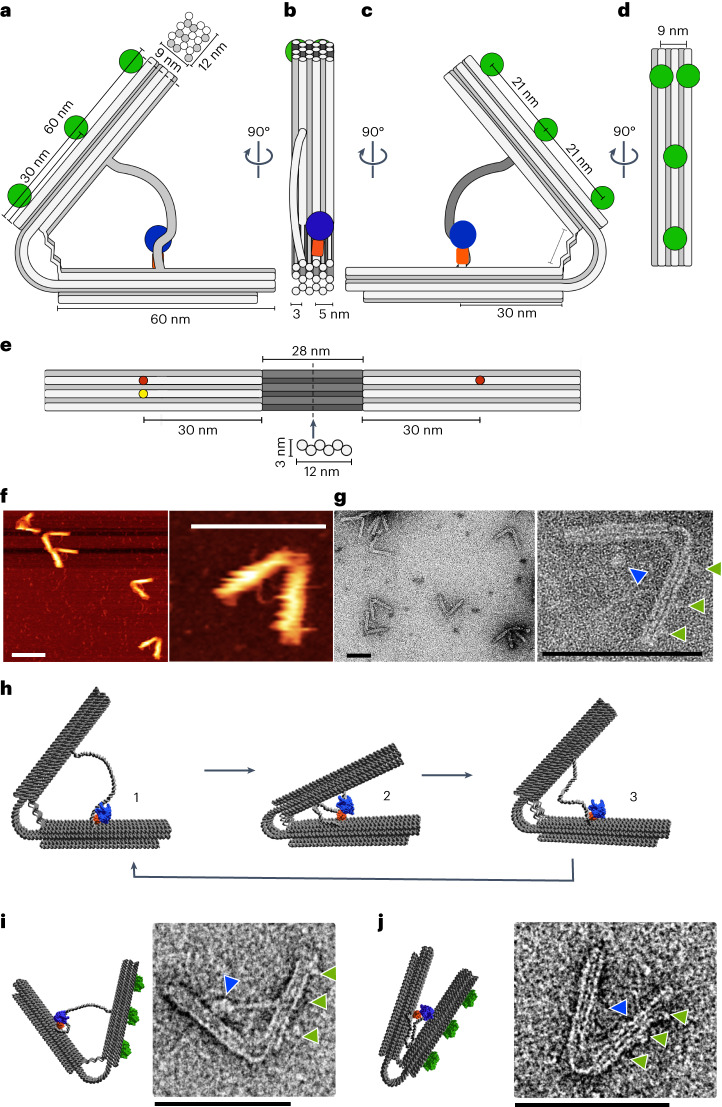
Design and dimensions of the DNA leaf-spring nanoengine. **a**, Schematic of the leaf-spring nanoengine, showing the dimensions of the stiff origami arms. Green circles, attachment sites for streptavidin binding; blue circle, T7RNAP part of the HT–T7RNAP fusion protein. Top: arrangement and dimensions of the 18-helix bundle that forms the origami arms. **b**, Schematic of the 90° left turn of the view shown in **a**. Orange rectangle, HT; blue circle, T7RNAP. **c**, Schematic and dimensions of the 90° left turn of the view shown in **b**. **d**, Schematic and dimensions of the 90° left turn of the view shown in **c**. **e**, Schematic and dimensions of the inner surface of the origami arms flanking the 28-nm-long leaf-spring helices (dark grey) that are arranged in a sliced honeycomb lattice fashion (below). Red dots, attachment sites of the dsDNA template strand; yellow dot, attachment site of the HT–T7RNAP. **f**, AFM characterization of the leaf-spring nanoengine. Overview (left) and detailed image (right) of the nanoengines. **g**, TEM of the nanoengine in negative staining. Overview (left) and detailed image (right) of the nanoengines. Green arrows, streptavidin molecules bound to biotin-modified staples protruding from one of the origami arms opposite to the location of the HT–T7RNAP fusion protein (blue arrow). **h**, Full opening and closing cycle of the compliant mechanical structure: (1) in the open structure the dsDNA template is bound by the immobilized HT–T7RNAP fusion protein and transcription begins; (2) upon transcription, HT–T7RNAP pulls the opposing origami arm towards itself, forcing the structure to close; (3) when the terminator sequence is reached, the T7RNAP releases the dsDNA template linker, which causes the structure to actively open to its equilibrium conformation. The T7RNAP can initiate the next closing cycle. **i**, Example of the nanoengine engaged in transcription. Blue arrow, HT–T7RNAP; green arrows, streptavidin. **j**, Example of the nanoengine engaged in transcription. Blue arrow, HT–T7RNAP; green arrows, streptavidin. All scale bars, 100 nm. Source data

**Extended Data Fig. 1:**
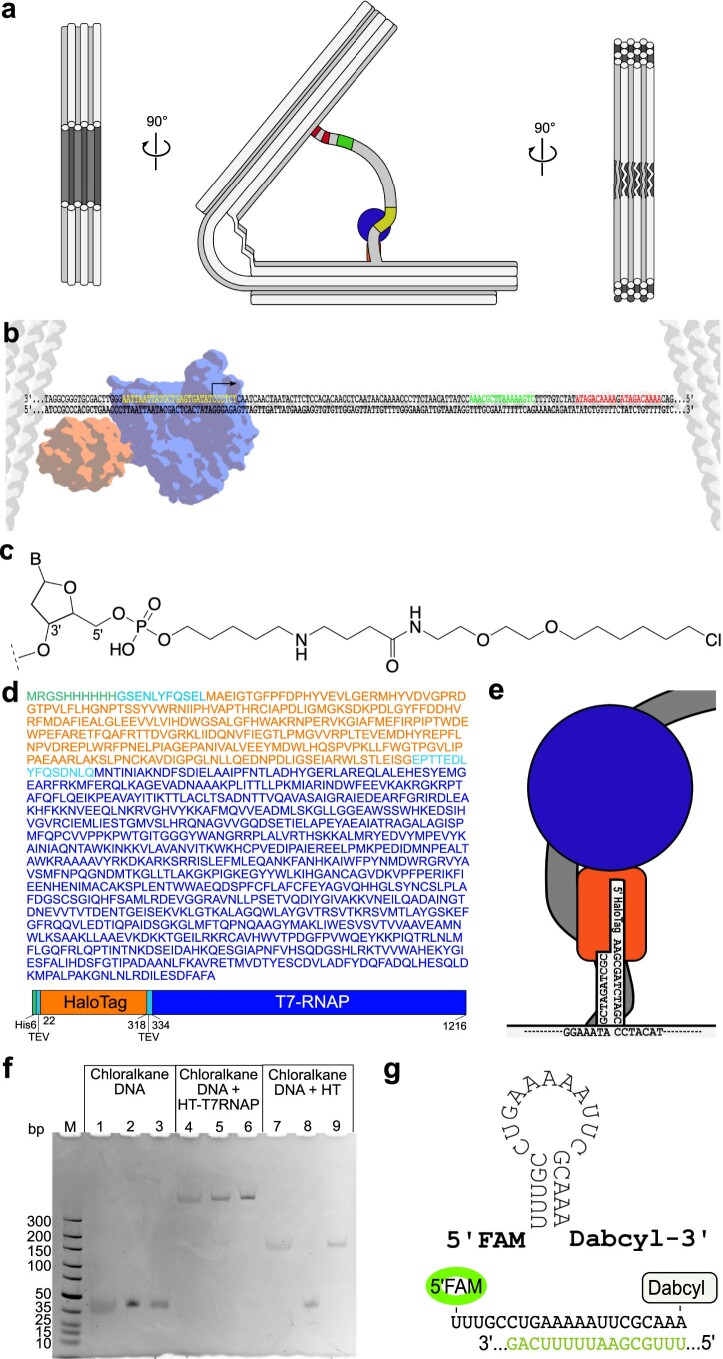
Design features of NE and molecular beacon. Design features of the nanoengine and molecular beacon (**a**) Schematic of the nanoengine with the leaf-spring (left panel), a side-view (middle) that shows the dsDNA template (dsDNA-t) and the location of the T7 promoter (yellow), the sequence coding for a binding site for a molecular beacon to determine transcription yields (green), and the two terminator sequences (red). The right panel shows the location of the six ssDNA sequences in the hinge area. (**b**) Sequence details of dsDNA-t. Yellow: T7 promoter, green: sequence coding for molecular beacon-binding, red: terminator sequence. (**c**) Chemical structure of the halogenated 5′-end of the protruding HT–T7RNAP attachment staple. (**d**) Primary amino acid sequence (upper panel) and design (lower panel) of the HT–T7RNAP fusion protein. (**e**) Schematic of the HT–T7RNAP and sequence of the protruding staple containing the 5′-halogenated attachment site. (**f**) Chloroalkane DNA connection to the HaloTag (HT) enzyme. Lanes 1-3: Chloralkane DNA in (1) origami buffer, (2) H_2_O, (3) Origami buffer + EDTA; lanes 4-6: Chloralkane DNA + HT–T7RNAP in (4) origami buffer, (5) H_2_O, (6) Origami buffer + EDTA; lanes 7-9: Chloralkane DNA + HT in (7) origami buffer, (8) H_2_O, (9) Origami buffer + EDTA. The Halo enzyme alone in absence of MgCl_2_ cannot bind to the chloroalkane modified DNA. In presence of the buffer the protein can bind to the DNA and the connection is covalent and strong enough that even after addition of EDTA to remove the MgCl_2_ the connection of DNA and protein is maintained. In the fusion protein of Halo T7 RNA pol this phenomenon is not present, likely due to the affinity of the polymerase towards DNA that probably increases the affinity of the fused Halo tag towards the DNA. (**g**) Sequence, secondary structure, and labels (5′-FAM, 3′-Dabcyl) of the molecular beacon RNA that detects the green sequence in the RNA generated during transcription.

**Extended Data Fig. 2:**
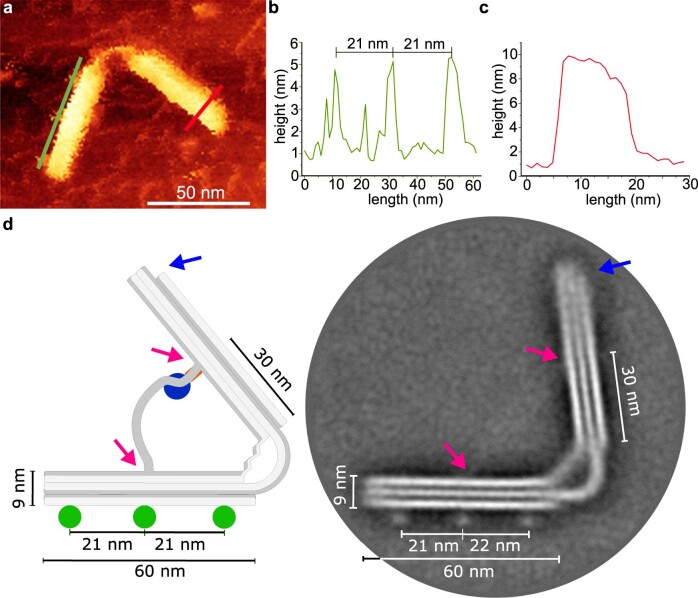
Assembly verification of the NE by AFM and TEM. Assembly verification of the nanoengine by AFM and TEM. (**a**) Detailed AFM image of the origami structure; green line: spans the streptavidin molecules attached to the respective origami arm; red line: marks the cross-section of the opposing origami arm. (**b**, **c**) Height profiles of the green and red lines confirming the distance of the streptavidin molecules on the respective origami arm to be spaced exactly 21 nm as designed (**b**), and the cross-section of the opposing origami arm to be exactly 9 nm as designed (**c**). (**d**) Comparison between the nanoengine design (left) and a 2D average image obtained from negative stained TEM micrographs (right). The 2D average shows an origami arm length of 60 nm as per design. The presence of streptavidin, depicted as green dots on the image on the left are visible in the 2D average as 21-22 nm evenly spaced bright dots on the outside of the horizontal origami arm. The pink arrows point to dsDNA-t, in particular where the strand contacts the origami, that is visible as a fuzzy shadow in the 2D average image. The blue arrow points to a nicked corner of the upper origami arm, a feature found in the 2D average. Source data

The design features of the rhythmic pulsating leaf-spring DNA nanomachine are described in detail^21–27^ in Supplementary Chapter 1 (Fig. 1a–j, Extended Data Figs. 1a–g and 2a–d, Supplementary Datasets 1 and 2, Nanobase^22^ entry https://nanobase.org/structure/196 and Supplementary Movie 1).

**Fig. 2:**
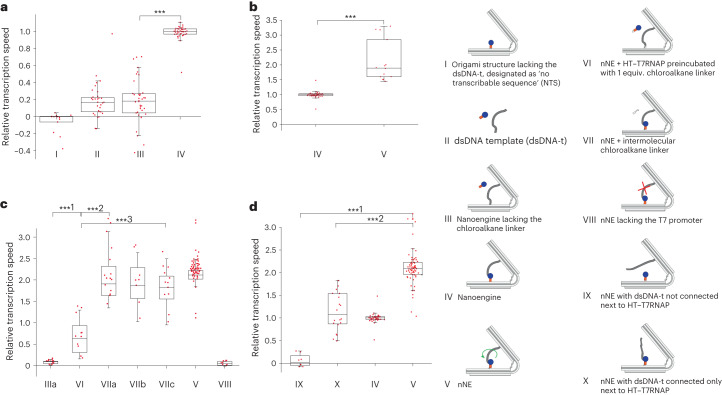
Structural characterization by bulk transcription experiments. **a**–**d**, Transcriptional rates were determined from the linear fit during the linear growth phase of fluorescence increase due to MB binding to the transcribed RNA (Supplementary Dataset 4 and Supplementary Fig. 2b,c). All rates relate to the transcriptional rate of the nanoengine. **a**, Column I, NTS; column II, intermolecular transcription rate from the dsDNA-t incubated with HT–T7RNAP; column III, nanoengine lacking the chloroalkane linker; column IV, nanoengine (I: *n* = 14, −0.06 ± 0.12, min = −0.38, max = 0.04, median = 0.00; II: *n* = 32; 0.16 ± 0.15, min = −0.14, max = 1.48, median= 0.16; III: *n* = 38, 0.19 ± 0.26, min = −0.42, max = 0.70, median = 0.18; IV: *n* = 40, 1.00 ± 0.12, min = 0.52, max = 1.48 median = 1.00. ***P = 3.0 × 10^−23^). **b**, Transcription rates of the nanoengine (column IV) compared with the nNE (column V) (IV: *n* = 40; 1.00 ± 0.12, min = 0.52, max = 1.48, median = 1.00; V: *n* = 14, 2.12 ± 0.69, min = 1.45, max = 3.30, median = 1.89. ***P = 3.9 × 10^−5^). **c**, Column IIIa, nNE lacking the chloroalkane linker; column VI, nNE + HT–T7RNAP preincubated with 1 equiv. of the chloroalkane linker; columns VIIa–c, nNE in the presence of 1 (VIIa), 2 (VIIb) and 5 (VIIc) equiv. of the chloroalkane linker; column V, nNE (IIIa: *n* = 14, 0.08 ± 0.04, min = 0.01, max = 0.15, median = 0.09; VI: *n* = 12, 0.65 ± 0.40, min = 0.16, max = 1.30, median = 0.63; VIIa: *n* = 14, 2.03 ± 0.53, min = 1.35, max = 3.13, median = 1.91; VIIb, *n* = 8, 1.89 ± 0.55, min = 1.03, max = 2.64, median = 1.87; VIIc: *n* = 12, 1.77 ± 0.44, min = 0.95, max = 2.49, median = 1.82; V: *n* = 56, 2.12 ± 0.32, min = 1.04, max = 3.19, median = 2.12; ***^1^*P* = 0.0005, ***^2^*P* = 9.6 × 10^−8^, ***^3^*P* = 1.6 × 10^−6^); column VIII: nNE lacking the T7 promoter (*n* = 8, 0.05 ± 0.04, min = −0.01, max = 0.10, median = 0.05). **d**, Transcription rates of constructs with different attachments of dsDNA-t to the origami: column IX, nNE with dsDNA-t not connected next to HT–T7RNAP; column X, nNE with dsDNA-t connected only next to HT–T7RNAP (both dsDNA-t have a nick at the single attachment site); column IV, nanoengine; column V, nNE (IX: *n* = 8, 0.06 ± 0.14, min = −0.07, max = 0.27, median = 0.02; X: *n* = 20, 1.15 ± 0.43, min = 0.50, max = 1.83, median = 1.08; IV: *n* = 40; 1.00 ± 0.12, min = 0.52, max = 1.48, median = 1.00; V: *n* = 70, 2.12 ± 0.41, min = 1.04, max = 3.30, median = 2.09; ***^1^*P* = 1.6 × 10^−21^, ***^2^*P* = 7.8 × 10^−10^). *P* values were obtained with a two-tailed, heteroscedastic *t*-test. Box-plot edges, 25th and 75th percentiles; box lines, 50th percentiles; whisker sizes, 1.5 × interquartile range (IQR); red dots, single datapoints. Error ranges are mean and s.d.

To investigate the functionality of the nanoengine, we used excess molecular beacon (MB) molecules present in solution to quantify RNA transcription of the transcribable double-stranded DNA template (dsDNA-t) strand (Fig. 2, Supplementary Fig. 2 and Supplementary Datasets 3 and 4). The importance of the covalent attachment of the HaloTag (HT)–T7RNAP was corroborated by comparing the transcriptional rates of multiple design variants directly to that of the nanoengine (Fig. 2a). Notably, the origami structure lacking the dsDNA-t (designated as no-transcribable sequence, NTS) showed a negative transcription rate, probably due to slow photobleaching (Fig. 2a, column 1). The rate of transcription of the dsDNA-t alone by the HT–T7RNAP controls for the transcription efficiency in an intermolecular state (column 2). A nanoengine lacking the chloroalkane attachment site, preventing the HT–T7RNAP from covalently attaching to the origami provides another intermolecular transcription control (column 3). Accordingly, the transcription rate is comparable to that in column 2, indicating that the origami does not present steric hindrances. The fully assembled nanoengine (column 4) has a transcription rate approximately five times higher than the negative controls (columns 2 and 3). From these bulk experiments, we estimate that 2.3 ± 0.8 (*n* = 6, mean and error from s.d.) transcripts are produced per minute. The higher transcriptional efficiency of the nanoengine with covalently bound HT–T7RNAP compared with structures in which HT–T7RNAP is not covalently bound can be attributed to the proximity effect and high local concentration due to colocalization of T7RNAP and promoter.

During transcription, the polymerase needs to unwind the dsDNA-t that, due to its anchoring to the origami arms, will accumulate torsional stress as transcription proceeds. Only upon release of HT–T7RNAP can this torsional stress be relieved, to rebuild in the next transcription cycle. The accumulation of torsional stress can be counteracted by introducing two single-stranded nicks in the dsDNA-t strands close to the points of connection of the dsDNA-t to the origami (Supplementary Fig. 3, red arrows). This slight structural modification permits rotation along the axis of the dsDNA-t without any accumulation of torsional stress. Accordingly, we observed a twofold increase in the rate of transcription for the nanoengine construct containing the two nicks (nicked-nanoengine, nNE) compared to the non-nicked sequence (Fig. 2b).

**Extended Data Fig. 3:**
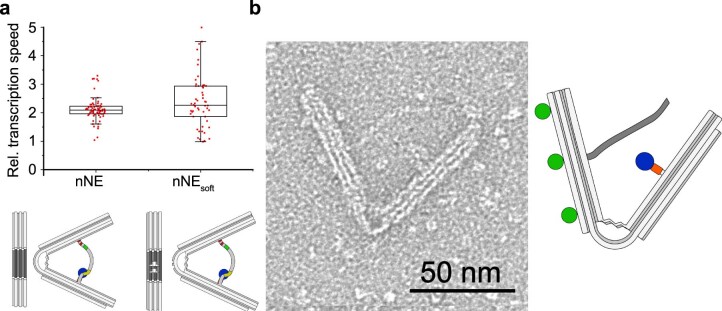
Influence of altered hinges on transcription. (**a**) Hinge effect on transcription rate determined by the relative transcription rate of the nicked-nanoengine (nNE, left) and nicked-nanoengine_soft (nNE_soft_, right). nNE: n = 70, 2.12 ± 0.41 mean and SD, min=1.04 max=3.30 median = 2.09; nNE_soft_: n = 52, 2.39 ± 0.92 mean and SD, min=0.98 max=4.99 median = 2.26; Box-plot edges: 25^th^ and 75^th^ percentiles. Box lines: 50^th^ percentile. Whisker size: 1.5× the IQR, red dots: single datapoints. Error ranges: mean and S.D. (**b**) Representative TEM image (left panel) of construct **IX** shown in Fig. 2d. The detached end of dsDNA-t extends out of the inside of the origami. Right panel: schematic of the structure shown in the TEM micrograph. Source data

To test whether changes in the compliant hinge region also influence the transcription rate, we removed two staples from the flat spring region to create two ‘holes’ in the double-stranded origami fabric, leaving only two helix strands continuously double-stranded, whereas the others are partially single-stranded (Extended Data Fig. 3a). We hypothesized that this spring softening of the nNE would decrease the resistance of the hinge region to the closure of the origami structure, which may increase the rate of transcription. Somewhat unexpectedly, however, we observed a comparable transcription rate of nNE_soft_ and the genuine nNE, an effect explained in the chapter on molecular dynamics simulations.

As seen before for the nanoengine, an nNE lacking the chloroalkane linker is virtually inactive (Fig. 2c, column 1). Preincubation of the HT–T7RNAP with 1 equiv. of the chloroalkane linker to saturate the HT binding site preventing its covalent attachment to the nNE origami similarly resulted in a slow transcription rate of 0.7 relative to the nanoengine (column 2). In contrast, the addition of increasing concentrations of 1, 2 and 5 equiv. of free chloroalkane linker to an already assembled nNE with attached HT–T7RNAP only minimally affected its relative transcription rate (columns 3–5), as expected. A version of the nNE in which the dsDNA-t lacked the promoter region (Supplementary Fig. 3b) was virtually inactive (Fig. 2c, column 7).

Finally, to explore the influence of the dual attachment of the dsDNA-t on the relative transcription rate, we tested versions of the nNE in which dsDNA-t was attached only to one or the other of the two origami arms (Fig. 2d, Extended Data Fig. 3b and Supplementary Fig. 3c,d). The corresponding results and their interpretations are described in Supplementary Text 2.

## Angle distribution statistics of nNE arms confirms pulsation

**Fig. 3:**
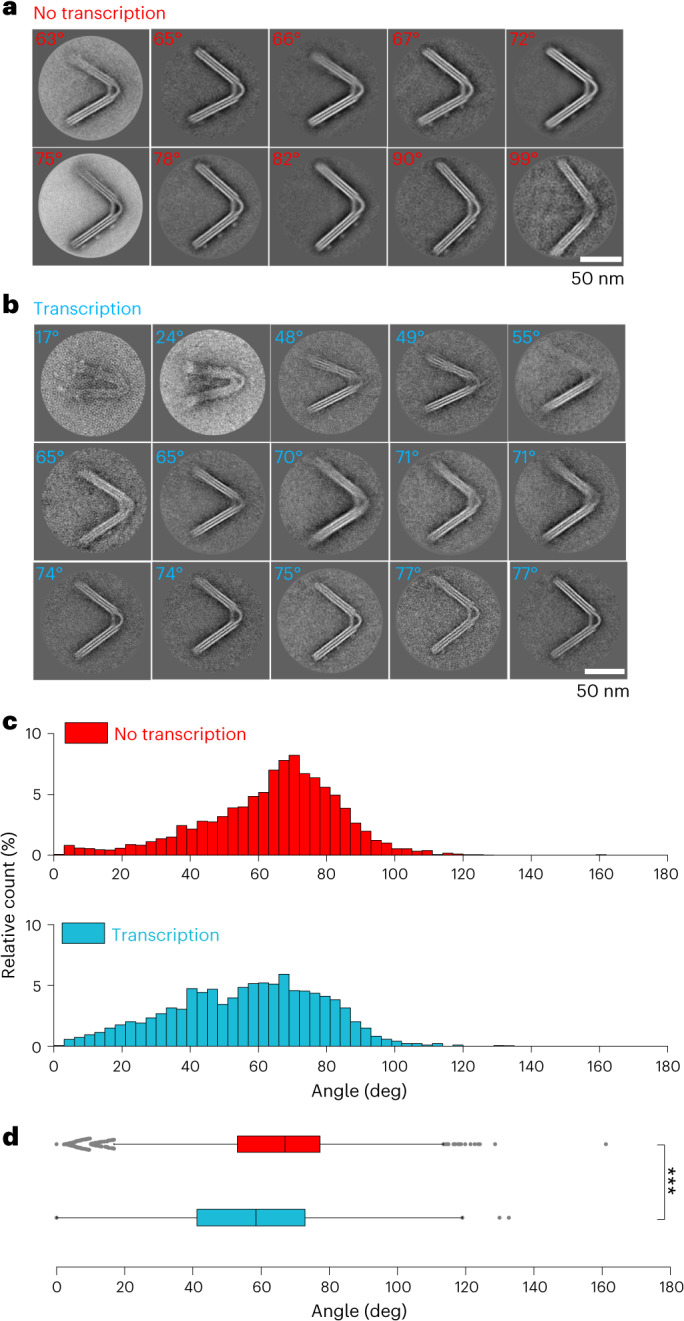
2D averages from negatively stained TEM micrographs of the nNE no-transcription sample and the nNE transcription sample. **a**, 2D classes with identifiable angles of the nNE no-transcription sample (65 micrographs, 2,502 particles). The 2D averages depict structures with opening angle ranges that go from 63° up to 99°. **b**, 2D averages of the nNE transcription sample (80 micrographs, 1,903 particles) with classes that depict structures with opening angles ranging from 17° to 77°. **c**, Comparison of the angle distribution under the condition of transcription and no transcription. Red, distribution of angles in nanoengines deposited on TEM grids that did not undergo transcription (*n* = 5,135, 189 micrographs, 64.13° ± 20.04°, min = 0.00°, max = 161.11°, median = 67.03°); cyan, distribution of angles in nanoengines that underwent transcription deposited on TEM grids (*n* = 3,266, 99 micrographs, 56.51° ± 21.79°, min = 0.00°, max = 132.64°, median = 58.43). The angles were measured using the angle measurement tool of ImageJ software (Supplementary Dataset 4, Extended Data Fig. 4 and Supplementary Table 1). Percentages of relative counts were calculated by dividing the counts of each bar (bin width, 3°) by the total amount of data in the population. **d**, Box-plot of angle distributions for the nanoengines not undergoing transcription (red) and the nanoengines that are engaged in transcription (cyan). ***P = 3 × 10^−57^. *P* values were obtained with a two-tailed, heteroscedastic *t*-test. Box-plot edges, 25th and 75th percentiles; box lines, 50th percentiles; whisker size, 1.5 × IQR; grey dots, outliers. Error ranges are the mean and s.d.

**Extended Data Fig. 4:**
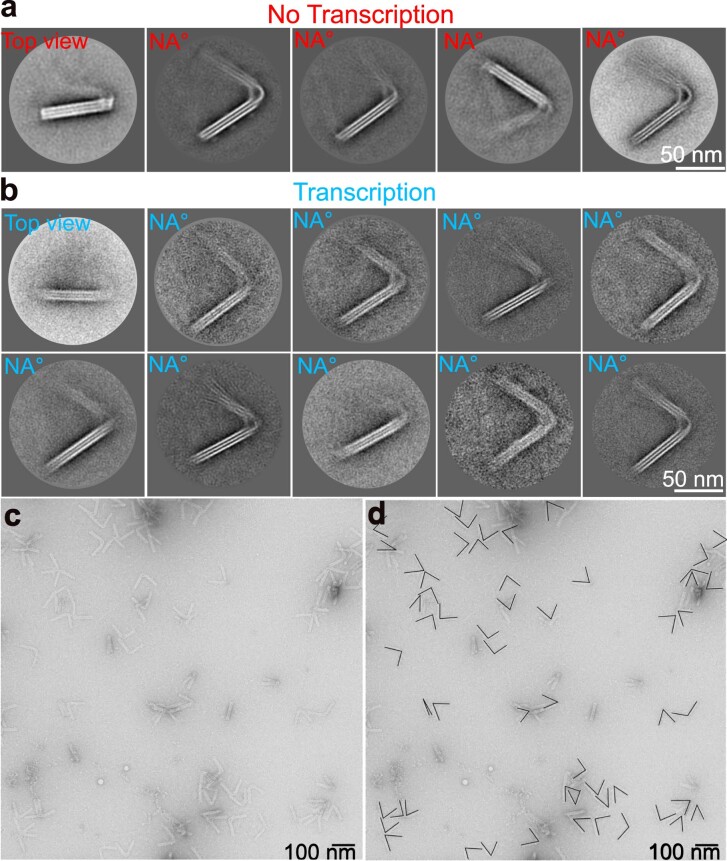
Exemplary 2D classes and exemplary angle measurement under no transcription and transcription conditions. (**a**) Representation of 2D classes of the nicked-nanoengine no transcription sample that depict a top view and four more averaged classes with no clearly discernible features of the structures that hindered a clear angle determination. (**b**) 2D classes of nicked-nanoengine transcription sample with a top view image in the upper left corner and 2D classes where no clear identification of the opening angle was possible. (**c**) Example of raw data of a TEM image. For angle measurements, only structures that lay on the surface were considered. These were identified based on visibility and correct form of the hinge structures as well as clear visibility of the axial dsDNA patterns in the origami arms. (**d**) Examples of angle measurements (black).

To get an overview of the angle distribution, we performed two-dimensional (2D)-average classifications of transmission electron microscopy (TEM) micrographs of nNEs with and without transcription. The 2D classifications were obtained using RELION^28^ (Methods and Fig. 3a,b). The two data sets, nNE without transcription (nNE no transcription, Fig. 3a) and with transcription (nNE transcription, Fig. 3b), both yield 2D averages of the origami structures with the most abundant aperture angles of ~70°, while other classes are less well defined. Importantly, lower aperture angles were too rare to generate a separate class for the nNE sample without transcription. In contrast, analysis of the nNE transcriptional sample revealed 2D classes with angles as small as 17°, indicating the presence of origami with smaller aperture angles (Supplementary Table 1). This observation supports the expected behaviour that the nNE increases the number of origamis with acute angles under transcription conditions. Of note, both samples also produced additional 2D averages without clearly identifiable features and without measurable angles (Extended Data Fig. 4a,b and Supplementary Text 3).

To get a better understanding of the angle population we used the thousands of nNE structures visible in the TEM micrographs to statistically compare the angle distributions of the two origami arms in nNEs that did not undergo transcription with those that did (Supplementary Methods, Supplementary Dataset 4 and Extended Data Fig. 4c,d). Due to the transitional nature of the opening and closing process, bulk experiments like these will always result in angle distributions over a fairly wide range, but we expected a broader angle distribution with a higher proportion of acute angles in the nNE samples engaged in transcription. The reference sample ‘no-transcription’ (Fig. 3c, red) shows a skewed distribution of angles that increases from 20° to a maximum at 70° and descends rapidly towards zero for larger angles, indicating that a stretching of the structure above a certain angle is disfavoured. Angles below 20° are evenly distributed with low frequency. By comparison, the transcription sample (Fig. 3c, cyan) exhibits a more dispersed distribution of angles, with the median shifting towards more acute angles. A significantly smaller number of nNEs adopt angles >60°, while angles between 10° and 60° are more prevalent.

Box-plots depicting the angle distributions (Fig. 3d) show a narrower angle distribution of the non-transcribed nNEs (red) than the transcribed ones (cyan). This observation indicates that transcription promotes closing of the nNE and thus induces smaller angles and less time spent in the large angle, ‘open’ equilibrium conformations. Indeed, the average angle and other distribution parameters for the nNE under transcription conditions always shift towards more acute angles. The shift towards acute angles is statistically highly significant (Fig. 3d) and demonstrates that a new population of structures with smaller angles between the arms emerges during transcription.

The loss of a defined narrow peak in the angle distribution in favour of more acute angles of nNE upon transcription indicates a lack of one dominant equilibrium conformation, implying that the origami structure becomes more heterogeneous and dynamic. This is consistent with our postulated mechanism in which the active closure of the two origami arms produced by the pull of the immobilized HT–T7RNAP on the dsDNA-t is counterbalanced by the mechanical properties of the leaf-spring.

## Single-molecule FRET quantifies kinetics of nNE pulsation

**Fig. 4:**
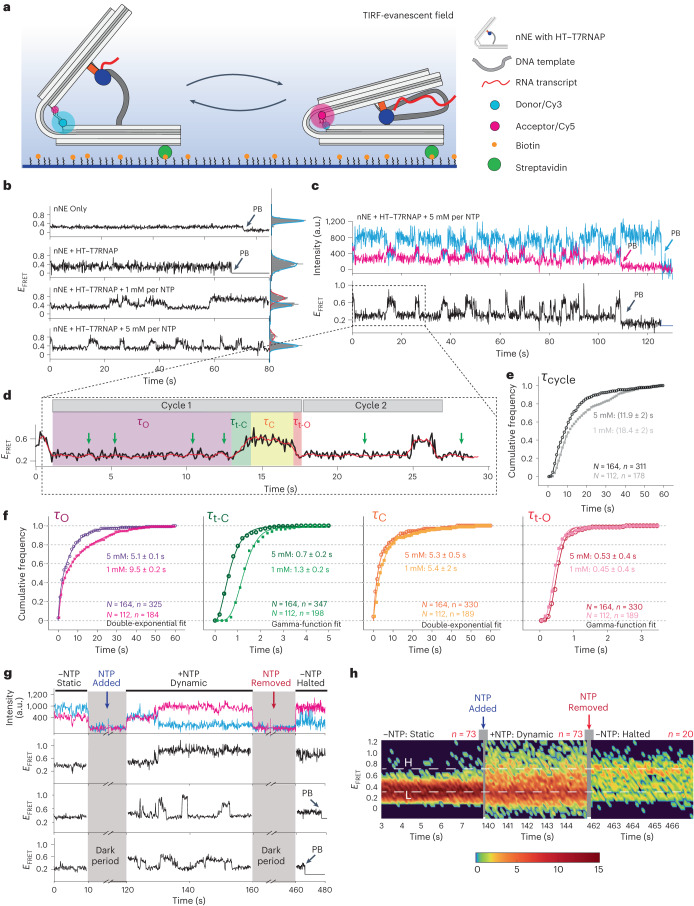
Single-molecule kinetic analysis of the nNEs. **a**, Schematic of the smFRET assay. FRET between the Cy3 (cyan) donor and the Cy5 (magenta) acceptor dyes monitors the distance between the two arms of the nNEs. **b**, Representative FRET time traces of single nNEs under varying conditions. Static traces are observed in the absence of either HT–T7RNAP or NTPs; dynamic traces are observed only when both were present. Arrows, dye photobleaching (PB). Right: histograms for each trace showing the low- (blue) and high-FRET (red) states. **c**, Representative fluorescence time trajectory of a single nNE in the presence of 5 mM of each NTP. The anticorrelated intensities of Cy3 and Cy5 are monitored until Cy5 and/or Cy3 photobleach. The smFRET trajectory (black) shows multiple transitions between two dominant FRET states. **d**, Representative segmentation analysis of a dynamic smFRET trace reveals the cycle time (*τ*_cycle_) of nNE opening–closing events, subdivided into low-FRET-state time (*τ*_O_), transition time from low- to high-FRET (*τ*_t-C_), high-FRET-state time (*τ*_C_), and transition time from high- to low-FRET (*τ*_t-O_). Green arrows, abortive transcription events. **e**,**f**, Cumulative distributions for *τ*_cycle_ (**e**) and individual time components (*τ*_O_, *τ*_t-C_, *τ*_C_, *τ*_t-O_) (**f**) for 1 mM and 5 mM NTP conditions (lighter and darker colour, respectively). Number of molecules (*N*) and transitions (*n*) are shown at the bottom of each plot. *τ*_O_ and *τ*_C_ were fitted with double-exponential functions, *τ*_tF_ and *τ*_tR_ were fitted with gamma functions to obtain their respective transition time constants. Errors represent s.d. of three biological replicates. **g**, Single-molecule traces from non-equilibrium ‘NTP-switch’ experiments with segments before addition of NTPs (−NTP, static), after addition of NTPs (+NTPs, dynamic) and after removal of NTP (−NTP, halted). Grey axis break indicates the dark period between segments during which the buffer was exchanged. **h**, Heat map of all molecules in the respective states, representing a cumulative behaviour. Static molecules were observed in the low-FRET state in −NTP static phase. Upon NTP addition, the same molecules exhibited dynamic behaviour with transition between low-FRET (L) and high-FRET (H) states. When NTPs were washed out, molecules remain either in the H or L state. Colour bar, number of molecules in a particular state in the heat map.

**Extended Data Fig. 5:**
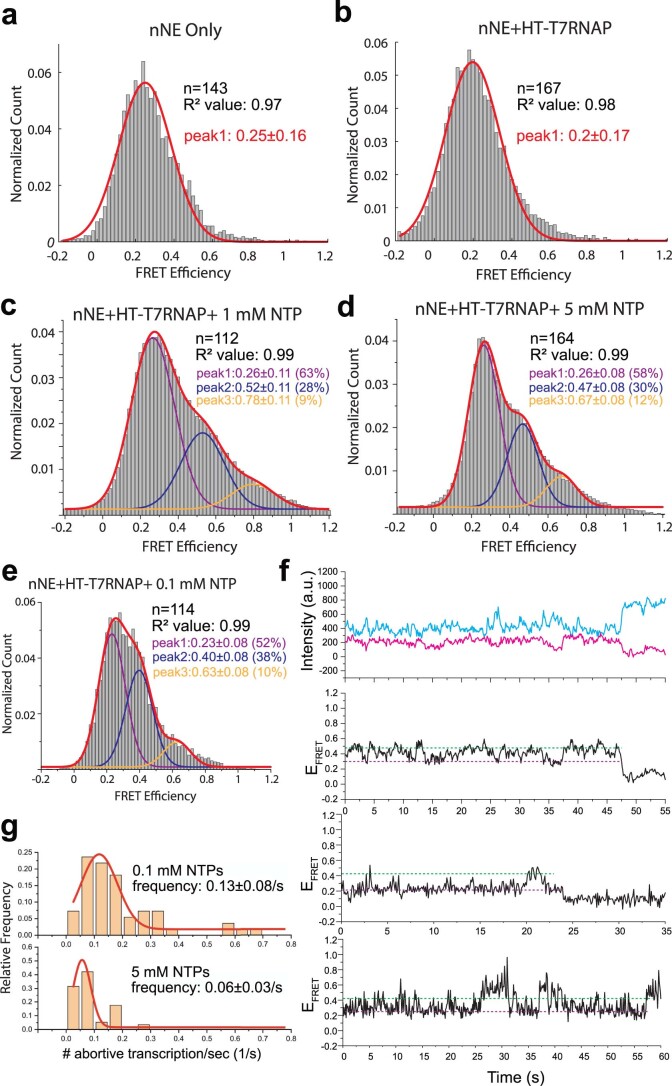
smFRET probability distributions. smFRET probability distributions of (**a**) nicked-nanoengine (nNE) only, (**b**) nicked-nanoengine with HT–T7RNAP, (**c**) nicked-nanoengine with HT–T7RNAP in the presence of 1 mM NTP mix, and (**d**) nicked-nanoengine with HT–T7RNAP in the presence of 5 mM NTP mix. (**e**) FRET probability distributions at 0.1 mM each NTP. (**f**) Representative smFRET traces showing incomplete/abortive transcriptions. (**g**) Histogram showing average number of abortive transcription/unit time spent in the open state in presence of 0.1 mM (top) and 5 mM (bottom) NTPs. Mean frequency and error (S.D.) are calculated by fitting the data to a single Gaussian function (red line).

Static measurements by atomic force microscopy (AFM) and TEM provide snapshots of the angle distributions of nNE populations. To directly monitor the pulsing motions of individual nNEs in real time, we developed a single-molecule Förster resonance energy transfer (smFRET) assay. nNE molecules were labelled with Cy3 donor and Cy5 acceptor dyes in appropriate positions on the two origami arms, then surface-immobilized using biotin–streptavidin linkages for observation via total internal reflection fluorescence microscopy (Fig. 4a). In the absence of HT–T7RNAP, smFRET trajectories (Fig. 4b, top trace) and the corresponding FRET efficiency (*E*_FRET_) histogram (Extended Data Fig. 5a) revealed a single broad peak around a mean FRET value of 0.25 ± 0.16 (mean ± s.d.). Based on a Förster distance (*R*_0_) of ∼54 Å (refs. ^29,30^) we estimate the distance between the two dyes to be ~6.8 nm, in agreement with the estimated distance of ∼7.1 nm for the open state observed by TEM (Supplementary Fig. 4). Attachment of HT–T7RNAP in the absence of NTP fuel resulted in a similar *E*_FRET_ histogram with only a slight decrease in the mean FRET value (0.2 ± 0.17; Extended Data Fig. 5b and Fig. 4b, second from top), which may be attributed to HT–T7RNAP binding.

Next, we introduced NTP fuel so that the HT–T7RNAP can actively transcribe the dsDNA-t in nNEs. Only under these conditions could we observe nNEs undergoing dynamic transitions from the low-FRET (*E*_FRET_ = 0.2 ± 0.15) to a new high-FRET (*E*_FRET_ = 0.7 ± 0.05) state and vice versa (Extended Data Fig. 5c,d). Representative time trajectories in the presence of either 1 mM or 5 mM of each NTP are shown in Fig. 4b (bottom two traces). We estimate the inter-dye distance in the 0.7 FRET state to be ~4.3 nm, again in good agreement with the distance of ~3.6 nm obtained for the smaller angles (~40°) in our TEM measurements (Supplementary Fig. 4), suggesting that the low- and high-FRET states reflect the fully open (O) and closed (C) nNEs, respectively. Repetitive *E*_FRET_ cycles show relatively slow transitions from the low-FRET to the high-FRET state and faster transitions from the high-FRET to the low-FRET state (Fig. 4c,d). Due to the slow inter-state transitions, consistent with the torsional strain expected in the dsDNA-t upon transcription, instead of two-state Markovian modelling, each full movement was segregated into four time segments (Fig. 4d–f), characterized by the dwell time of the low-FRET state (*τ*_O_, nNE open); transition time from low- to high-FRET state (*τ*_t-C_, nNE transition open → closed); dwell time in the high state (*τ*_C_, nNE closed); and transition time from high- to low-FRET state (*τ*_t-O_, nNE transition closed → open). NTP concentration correlates with the duration of the cycles in that increasing NTPs from 1 mM to 5 mM shortens the duration of each *E*_FRET_ cycle. The 1 mM NTP conditions showed slower cycles (18.4 ± 2 s) than the 5 mM NTP conditions (11.9 ± 2 s; Fig. 4e). These values are consistent with the emergence of one new transcript for each nNE every 12 ± 5 s (mean ± s.d.), as estimated in our bulk transcription experiments at 2 mM NTP. Notably, the smFRET experiments were carried out at 25 °C, while the bulk experiments were performed at 37 °C. It is therefore possible that the bulk experiments have an overall faster transcription rate that is reduced by some fraction of nanodevices being inactive.

From our smFRET traces, the individual dwell and transition time constants for each segment were extracted, revealing that increasing the NTP concentration from 1 mM to 5 mM reduces specifically *τ*_O_ from 9.5 ± 0.2 s to 5.1 ± 0.1 s (Fig. 4f). This observation is consistent with the expectation that the wait time for HT–T7RNAP to start a full transcription cycle decreases with increasing NTP concentration^31,32^. Similarly, the transition time *τ*_t-C_ decreased, consistent with the expected increased transcription speed of HT–T7RNAP at the higher NTP concentration^32,33^. In contrast, *τ*_C_ and *τ*_t-O_ remain essentially unchanged when changing the NTP concentration (Fig. 4f), consistent with the notion that they are determined by the intrinsic HT–T7RNAP termination time and leaf-spring constant of the origami structure, respectively. From *τ*_t-C_ values we can roughly estimate that at 25 °C the HT–T7RNAP transcribes 89–110 nucleotides (depending on where it stops in the terminator sequences) in 1.3 ± 0.2 s at a concentration of 1 mM for each NTP. At a concentration of 5 mM for each NTP, this timeframe reduces to 0.7 ± 0.2 s corresponding to an estimated rate of 68 nucleotides per second, which aligns well with previously reported values^34^.

We also found distinct small increases in *E*_FRET_ to ~0.4 that originate specifically from the open nNE state (as indicated by green arrowheads in Fig. 4d), consistent with the known^35^ abortive transcription when T7RNAP fails to transition from the initiation to the elongation phase^32,35–38^. These abortive events are more pronounced at low NTP concentration (0.1 mM) (Extended Data Fig. 5e–g), whereas they are not observed in the absence of NTPs (Fig. 4b, top trace and Extended Data Fig. 5b). In support of this observation, Extended Data Fig. 5f shows multiple representative time traces at 0.1 mM NTP where the distinct ~0.4 FRET state is observed. We found these abortive transcription events at this NTP concentration with a frequency of 0.13 ± 0.07 events per second or one event every ~7.6 s (Extended Data Fig. 5g, top). Notably, the rate of abortive transcription events reduced considerably at high NTP concentrations (Extended Data Fig. 5g, bottom) consistent with a previous report^32^. Taken together, our smFRET observations directly demonstrate that the nNE exhibits pulsing closing–opening cycles, as designed, while they also yield quantitative kinetic data.

## NTP concentration toggling stops and starts the nNE reversibly

The sustained opening and closing of individual nNE molecules under NTP equilibrium conditions raises the question of how they respond to non-equilibrium changes in their environment, and whether the nNE can be reversibly started and stopped by changes in, for example, NTP concentration. To address these questions, we performed an ‘NTP switch’ experiment from 0 to 5 mM of each NTP, followed by a ‘backward switch’ from 5 mM of each NTP to zero, while monitoring the distance between the two nNE arms by smFRET (Supplementary Methods).

FRET signals of surface-immobilized nNEs were initially recorded in the absence of NTPs (Fig. 4g and Methods), then in presence of 5 mM NTPs, and again in absence of NTPs (Fig. 4g), using the protocol described in the Methods. In the initial absence of NTPs, we only found the static low-FRET state, consistent with our equilibrium experiments (Fig. 4h, nNE + HT–T7RNAP condition). Upon addition of NTPs, the nNEs become active and undergo several cycles of reversible open–closed state transitions, as expected. Figure 4h presents a cumulative heat map of *n* = 73 molecules, showing that the molecules generally transition from a static low-FRET phase in the absence of NTPs to a much more dynamic FRET phase in their presence. Once NTPs are removed again, the nNEs remain stalled in the position they had at the moment the NTPs were removed, consistent with the long residence time of elongating T7RNAP on a template upon removal of NTPs^39^. That is, some nNEs remain in the high-FRET state (Fig. 4g, second from top) while others adopt the low-FRET state (Fig. 4g, bottom panel). In the cumulative heat map of the remaining unbleached nNEs, this stalling manifests in more discernible low- and high-FRET states with few transitions between them (*n* = 20, Fig. 4h).

## Molecular dynamics simulations of features governing opening/closing rates

**Fig. 5:**
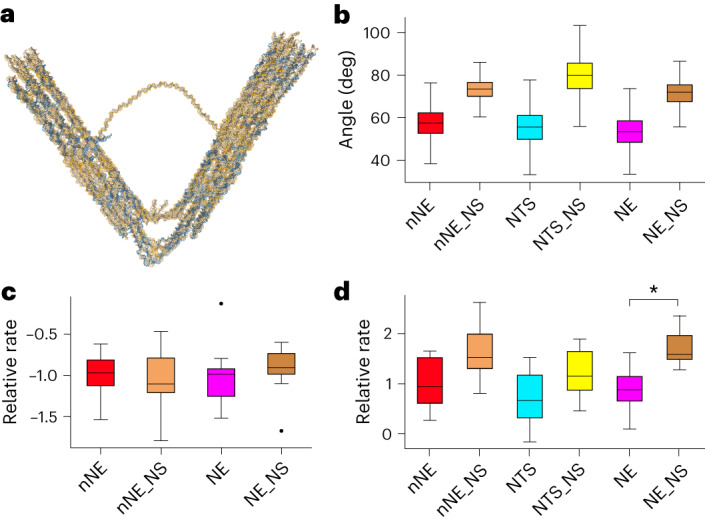
oxDNA simulations were performed to determine the dynamic structural properties of the nanoengine. **a**, Mean structure of an equilibrium sampling simulation of an nNE represented in oxDNA. **b**, Equilibrium angle distribution of six designs during oxDNA simulation. Regions where base pairing was turned off for single-stranded regions of the flexure to isolate the effect of secondary structures forming in the flexure are flagged with NS (no structure). nNE: *n* = 6,000, min = 26.6, max = 81.6, median = 58.4; nNE_NS: *n* = 6,000 min = 50.3, max = 88.8, median = 74.5; NTS: *n* = 5,971, min = 28.9, max = 81.7, median = 55.6; NTS_NS: *n* = 6,000, min = 49.3, max = 107.4, median = 80.0; nNE: *n* = 6,000, min = 26.6, max = 81.6, median = 58.4; nNE_NS: n = 6,000, min = 50.3, max = 88.8, median = 74.5; NE: *n* = 6,000, min = 27.8, max = 77.4, median = 53.5; NE_NS: *n* = 6,000, min = 46.4, max = 86.4, median = 72.0. **c**, Simulation-determined closing rates from pulling simulations relative to nNE. No significant difference in rate was observed. n = 10 for all boxes. nNE: min = −1.5, max = −0.6, median = −1.0; nNE_NS: min = −1.8, max = −0.5, median = −1.1; NE: min = −1.5, max = −0.1 median = −1.0; NE_NS: min = −1.7, max = −0.6, Q2 = −0.9 **d**, Simulation-determined opening rates relative to nNE from relaxation simulations where the forces from the pulling simulations were released and the structure allowed to open again. There is a trend toward higher opening rates in the NS simulations; however, NE and NE_NS are the only pair of corresponding structures where the difference is significant (*P < 0.05, calculated with a two-tailed Komogorov–Smirnov test). *n* = 10 for all boxes. nNE: min = 0.25, max = 1.65, median = 0.93; nNE_NS: min = 0.80 max = 2.63, median = 1.52; NTS: min = −0.17, max = 1.52, median = 0.66; NTS_NS: min = 0.45, max = 1.89, median = 1.15; NE: min = 0.09, max = 1.61, median = 0.87; NE_NS: min = 1.27, max = 2.36, median = 1.58. NE, nanoengine; NE_NS, non-structured nanoengine; nNE, nicked-nanoengine; nNE_NS, non-structured nicked-nanoengine; NTS, origami lacking dsDNA-t; NTS_NS, non-structured origami lacking dsDNA-t.

**Extended Data Fig. 6:**
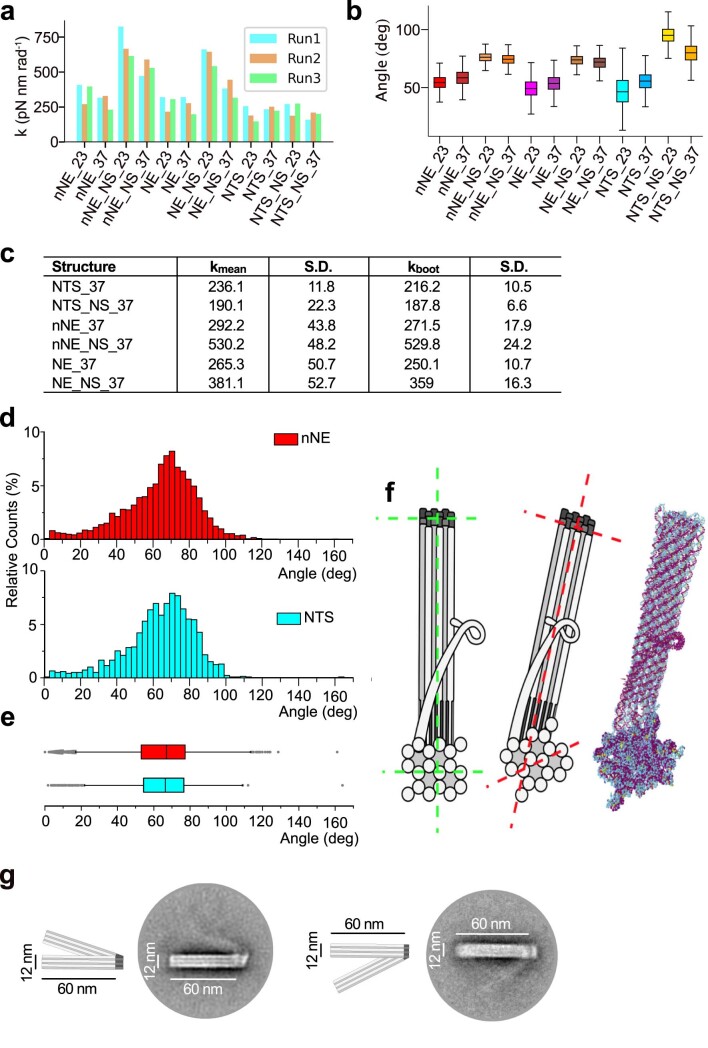
Hinge properties of simulations. **(a)** Calculated spring constant of each design at 23 °C and 37 °C. Only NS with an intact dsDNA-t show a substantial difference between 23 °C and 37 °C, demonstrating the influence of stable secondary structures on angle distribution. **(b)** Angle-distribution simulations (n = 3). At higher temperatures, simulations with secondary structures in the flexure resulted in a slightly increased average angle, while simulations showed a slight decrease when secondary structures were prevented. Presumably, secondary structures limit the opening angle of the structures, while the extended single strands behave more like entropic springs that become more flexible with increasing temperature (nNE_23: n = 5666, min=30.24, max=79.33 median = 54.35; nNE_37: n = 6000, min=26.56, max=81.57, median = 58.42; nNE_NS_23: n = 6000, min=49.62, max=89.42, median = 76.27; nNE_NS_37: n = 6000, min=50.28, max=88.75, median = 74.51; NE_23: n = 6000, min=25.98, max=76.02, median = 49.16; NE_37: n = 6000, min=27.79, max=77.43, median = 53.49; NE_NS_23: n = 6000, min=51.32, max=86.30, median = 73.90; NE_NS_37: n = 6000, min=46.39, max=86.39, median = 72.05,; NTS_23: n = 18000, min=13.09, max=86.33, median = 46.40; NTS_37: n = 5971, min=28.91, max=81.70, median = 55.62; NTS_NS_23: n = 6000, min=68.62, max=120.99, median = 95.14; NTS_NS_37: n = 6000, min=49.34, max=107.45, median = 80.01. (**c**) Estimation of spring constants of individual (nicked-)nanoengines at 37 °C based on the modelling studies, obtained either by taking the average and S.D. (n = 3) or by bootstrapping with 1,000 re-samplings of the combined data of the three replicates. (**d**,**e**) Experimental angle distributions of the nicked-nanoengine (red, n = 5135, 189 micrographs) and NTS (cyan, n = 1382, 28 micrographs) are highly comparable (p = 0.6, two-tailed, heteroscedastic *t*-test). (**e**) NTS: n = 1382, 63.84° ± 18.77°, min=1.68° max=164.00° median = 66.40; nNE: n = 5135, 64.13° ± 20.04°, min=0.00° max=161.11° median = 67.03°. (**f**) Relaxation of the nicked-nanoengine in simulation indicates that the origami arms bend out of plane showing a longitudinal twist in 18 HBs. Reference lines illustrate the amount of bending (green: designed structure, left; red: simulated structure, middle). Right: snapshot from a simulation of the nicked-nanoengine. (**g**) 2D-Averages of nicked-nanoengine top-views. One arm does not overlap the more resolved one, consistent with its simulation-predicted out-of-plane bending. The distortion of surface-deposited nicked-nanoengine likely explains the systematic difference in angle distributions between TEM imaging and simulation. Box-plot edges: 25^th^ and 75^th^ percentiles. Box lines: 50^th^ percentile. Whisker size: 1.5× the IQR, grey dots: outliers. Error ranges: mean and S.D. **Glossary** - nNE: nicked-nanoengine, nNE_23: nicked-nanoengine at 23 °C, nNE_37: nicked-nanoengine at 37 °C, nNE_NS_23: non-structured nicked-nanoengine at 23 °C, nNE_NS_37: non-structured nicked-nanoengine at 37 °C, NE_23: nanoengine at 23 °C, NE_37: nanoengine at 37 °C, NE_NS_23: non-structured nanoengine at 23 °C, NE_NS_37: non-structured nanoengine at 37 °C, NTS_23: origami lacking the dsDNA-t at 23 °C, NTS_37: origami lacking the dsDNA-t 37 °C, NTS_NS_23: non-structured origami lacking the dsDNA-t at 23 °C, NTS_NS_37: non-structured origami lacking the dsDNA-t at 37 °C; NE: nanongine, NE_NS: non-structured nanongine nNE: nicked-nanoengine, nNE_NS: non-structured nicked-nanoengine, NTS: hinge_origami_NTS, NTS_NS: non-structured hinge_origami_NTS.

**Extended Data Fig. 7:**
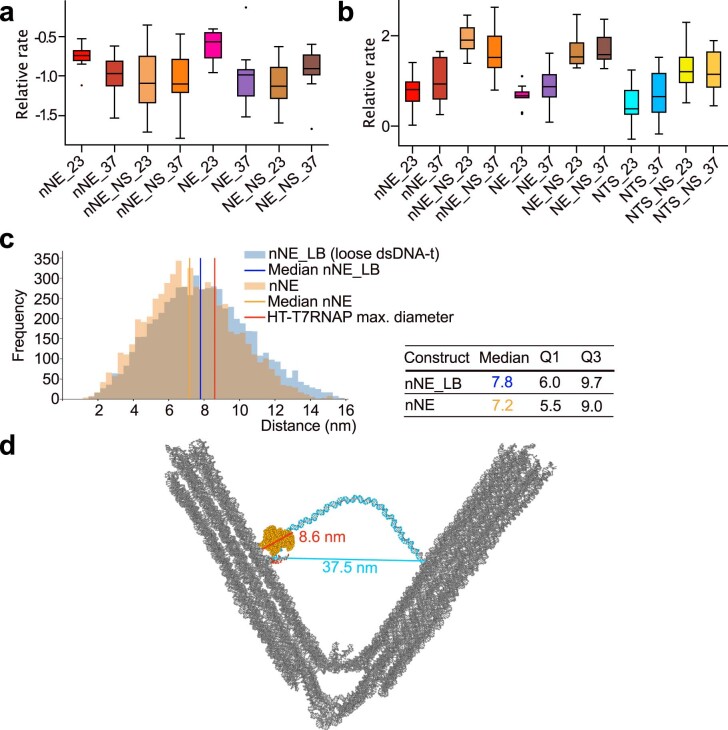
Calculated pulling and re-opening rates. Box-plot parameters in (**a**,**b**) Edges: 25^th^ and 75^th^ percentiles. Lines: 50^th^ percentile. Whisker size: 1.5× the IQR or the largest/ smallest point, whichever is closer to the centre. Black dots: outliers. (**a**) Calculated pulling rates relative to the mean of the nicked-nanoengine at 37 °C under 16 pN applied force between the polymerase attachment point and the terminator sequence on the dsDNA-t for each design at 23 °C and 37 °C. The only notable difference between the two temperatures was observed for the nicked-nanoengine and the nanoengine, where decreasing the number of base pairs in the flexure substantially increased the rate at which the leaf-spring was able to close (n = 10 for all boxes; nNE_23: min = −1.12, max = −0.53, Q2 = −0.74; nNE_37: mi= -1.54 max = −0.62, median = −0.97; nNE_NS_23: min = −1.72, max = −0.35, median = −1.09; nNE_NS_37: min = −1.79, max = −0.47, median = −1.10; NE_23: min = −0.96, max = −0.40, median = −0.57; NE_37: min = −1.52, max = −0.13, median = −0.99; NE_NS_23: min = −1.60, max = −0.63, median = −1.13; NE_NS_37: min = −1.67, max = −0.60, median = −0.91). (**b**) Calculated re-opening rates after release of the 16 pN force. At elevated temperature, both the average rate and the variance for the structures where secondary structures can form in the flexure are increased. If no structure was permitted in the flexure the average rate decreased and no trend in variance for structures was observed (nNE_23: min=0.02, max=1.41, median = 0.81; nNE_37: min=0.26, max=1.65, median = 0.93; nNE_NS_23: min=1.39, max=2.45, median = 1.90; nNE_NS_37: min=0.80, max=2.63, median = 1.52; NE_23: min=0.27, max=1.11, median = 0.67; NE_37: min=0.09, max=1.61, median = 0.87; NE_NS_23: min=1.29, max=2.47, median = 1.53; NE_NS_37: min=1.27, max=2.36, median = 1.58; NTS_23: min = −0.29, max=1.24, median = 0.39; NTS_37: min = −0.17, max=1.52, median = 0.66; NTS_NS_23: min=0.52, max=2.30, median = 1.21; NTS_NS_37: min=0.45, max=1.89, median = 1.15). (**c**) Histogram of the distance between the polymerase attachment site and the start of the T7 promoter on the dsDNA-t. The diameter of T7RNAP is ~8.6 nm. The frequency with which the nicked-nanoengine construct (orange, nNE) dwells within this ideal spacing bubble is higher than when the dsDNA-t is only attached next to T7RNAP (blue, nNE_LB). (**d**) oxView image showing the relative size of T7RNAP (orange; without HT, PDB ID: 3E2E)^45^ compared with the mean structure of the nicked-nanoengine (red: chloroalkane-modified overhang, blue: dsDNA-t). End-to-end length of dsDNA-t: 37.5 nm. Largest dimension of T7RNAP: ~8.6 nm. **Glossary** - nNE_23: nicked-nanoengine at 23 °C, nNE_37: nicked-nanoengine at 37 °C, nNE_NS_23: non-structured nicked-nanoengine at 23 °C, nNE_NS_37: non-structured nicked-nanoengine at 37 °C, NE_23: nanoengine at 23 °C, NE_37: nanoengine at 37 °C, NE_NS_23: non-structured nanoengine at 23 °C, NE_NS_37: non-structured nanoengine at 37 °C, NTS_23: origami lacking the dsDNA-t at 23 °C, NTS_37: origami lacking the dsDNA-t 37 °C, NTS_NS_23: non-structured origami lacking the dsDNA-t at 23 °C, NTS_NS_37: non-structured origami lacking the dsDNA-t at 37 °C.

We performed coarse-grained molecular dynamics (MD) simulations using the oxDNA model^40–43^ (Supplementary Chapter 2) to further characterize the impact of our design choices on the mechanical properties of the (nicked-)nanoengine (Fig. 5a,b and Extended Data Fig. 6a–f), on the behaviour of the nanoengine under tension (Supplementary Text 4, Fig. 5c,d and Extended Data Fig. 7a,b), and on the proximity effects of the distance between the HALO-tagged nucleotide and the T7 promoter in dsDNA-t^44,45^ (Extended Data Fig. 7c,d and Supplementary Text 6).

## An nNE driver can transfer its motion to a passive follower

**Fig. 6:**
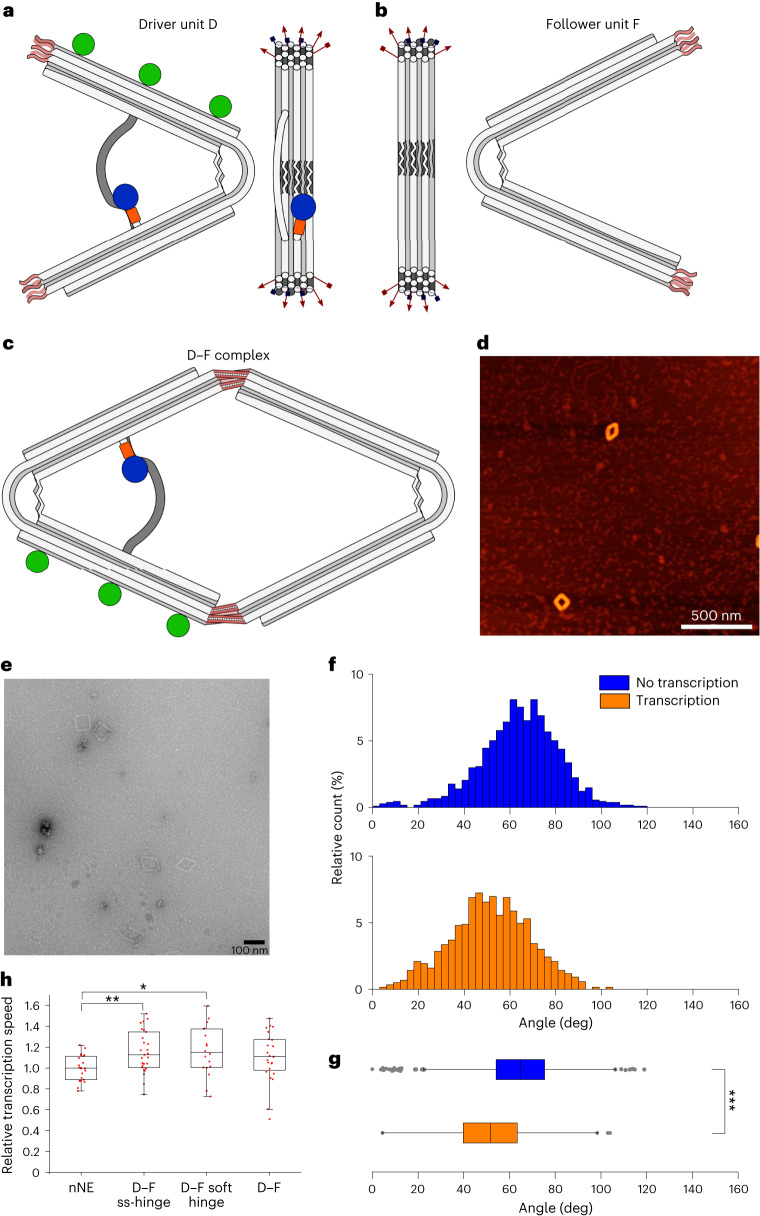
The nNE drives a passive follower unit. **a**,**b**, Schematic of the nNE driver D (**a**) and the passive follower unit F (**b**). The structures are modified to have ssDNA overhangs protruding from the stiff origami arms. Five ssDNA overhangs (red arrows) were introduced on each arm for a total of ten unique overhangs on each origami. The sequences allow the connection of only the active structure to the passive structure. Biotin modifications (green dots) are introduced only on D. Blue circle: polymerase, orange square: HaloTag portion of HT-T7RNAP. **c**, Assembly of the D–F heterodimer is achieved by equimolarly combining the two parts of the system and thermal annealing overnight. **d**,**e**, After 4.5 h of transcription experiments at 37 °C the integrity of the system is confirmed by AFM (**d**) (scale bar, 500 nm, unprocessed scan) and TEM (**e**) (scale bar, 100 nm, unprocessed micrograph). **f**, Angle distribution from TEM images of the D–F dimer complex for samples that did not (*n* = 1,074, blue, 90 micrographs) and did undergo transcription (*n* = 1,190, orange, 87 micrographs). The distribution is plotted as bar graphs with a bin with of 3°; the count of each bar is divided by the total number of counts and displayed as the relative count percentages. **g**, Box-plot representation of distributions. Blue, box-plot of D–F without transcription (*n* = 1,074, 64.09° ± 17.32°, min = 0.00°, max = 118.96°, median = 64.73°); orange: the same with transcription (*n* = 1,190, 51.37° ± 17.38°, min = 4.48°, max = 104.00° median = 51.66;). ***P = 8 × 10^−64^. **h**, The transcription speed of different D–F complexes compared with the transcription speed of D alone (*n* = 27, 1.00 ± 0.12, min = 0.78, max = 1.22, median = 1.00). Transcription speeds: D–F ss-hinge (*n* = 27, 1.15 ± 0.20, min = 0.75, max = 1.52, median = 1.13; **P = 0.001), D–F soft-hinge (*n* = 18, 1.16 ± 0.24, min = 0.73, max = 1.60, median = 1.15; *P = 0.01); D–F complex (n = 24, 1.10 ± 0.24, min = 0.51, max = 1.48, median = 1.11; *P* = 0.06). *P* values were obtained with a two-tailed, heteroscedastic *t*-test. Box-plot edges, 25th and 75th percentiles; box lines, 50th percentiles; whisker size, 1.5 × IQR; grey dots, outliers; red dots, single data points. Error ranges are the mean and s.d.

**Extended Data Fig. 8:**
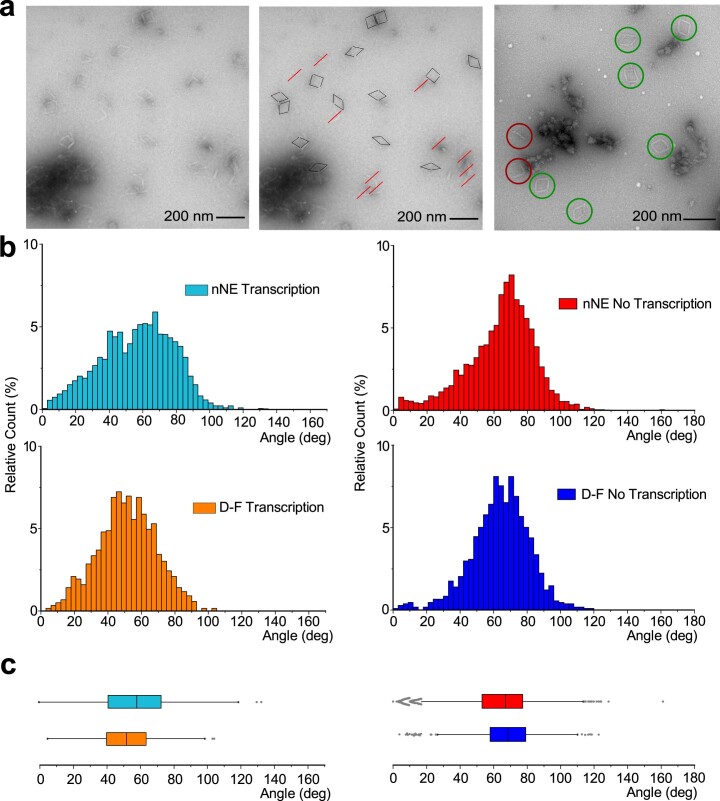
Angle distribution of different D–F constructs measured from TEM micrographs. (**a**) Example of TEM image used to determine the angle of the D–F complex (left panel) and with the overlayed black line to indicate how the angle were measured (middle panel). Not properly joined structures or single origamis have been ignored during the angle measurements (red strikethrough). The right panel is an example for D–F quantification. Green circles: correctly formed D–F-pairs, red circles: single origami or damaged structures. (**b**, **c**) Measuring the angle distribution using TEM images and comparing the distribution of nicked-nanoengine (red, n = 5135, 189 micrographs) with the angle distributions of complete D–F only (blue, n = 1074, 90 micrographs) in the absence of transcription, we notice that the average angle remains unchanged and is 64° ± 20° for the nicked-nanoengine and 64° ± 17° for D–F, but the curve is less skewed and more symmetric with the D–F complex. The same effect is also observed during transcription (nNE, cyan, n = 3266, 99 micrographs, D–F, orange, n = 1190, 87 micrographs). In the nicked-nanoengine sample, the average angle distribution drops to 57° ± 22°, which is comparable to the average value of the D–F complex of 57° ± 17°, but the curve is narrower in the second case. (**c**) Confirmation that the angle distribution in the case of the D–F complex is narrower and less skewed than that of the nicked-nanoengine alone (nNE Transcription: n = 3266, min=0.00° max=132.64° median = 58.45; nNE No Transcription: n = 5135, min=0.00° max=161.11° median = 67.03°; D–F Transcription: n = 1190, min=4.48° max=104.00° median = 51.66; D–F No Transcription: n = 1074 min=0.00° max=118.96° median = 64.73°). This stabilizing effect on the angle distribution is particularly pronounced with the D–F-no-hinge (Extended Data Fig. 9c). Box-plot edges: 25^th^ and 75^th^ percentiles. Box lines: 50^th^ percentile. Whisker size: 1.5× the IQR, grey dots: outliers. Error ranges: mean and S.D.

Any engine that actively performs work must be able to transfer its motion to passive moving parts. Nature has found a vast array of solutions for the transmission of force, such as in myocytes or adherent cells, but examples demonstrating nanomechanical force and motion transmission by synthetic machines are scarce and the motion occurs mostly stochastically^46–49^. To demonstrate that the nNE can act as a non-stochastic, autonomous mechanical ‘driver’ (D, Fig. 6a) to actively transmit its force to a passive part that follows its motion, we coupled the nNE to a similarly shaped, but passive ‘follower’ (F, Fig. 6b) to form a defined D–F pair (Fig. 6c–e). The required design features and sequences are specified in Supplementary Methods, Supplementary Dataset 1 and Supplementary Fig. 5a,b. We evaluated the percentage of the proper D–F duplex formation from TEM micrographs to be 60% ± 20% (mean and error from s.d., 199 TEM micrographs, *n* = 2,718; Extended Data Fig. 8a, Supplementary Dataset 5).

As before for the nNE, angle distributions under the transcription and no-transcription conditions were obtained by analysing large sets of TEM images of the rhomboidal D–F dimers (Fig. 6f and Extended Data Fig. 8a). Box-plots of the distribution (Fig. 6g and Supplementary Dataset 4) show that the average angle and other distribution parameters shift towards more acute angles for D–F that underwent transcription. Moreover, the formation of the D–F complex apparently leads to slightly narrower, less skewed and more symmetric angle distributions under both conditions (Extended Data Fig. 8b,c, blue, orange) compared with the nanoengine (red, cyan), suggesting that the linking of D and F has a stabilizing effect on the angle. These data demonstrate that actively transcribed D–F structures exhibit significantly more acute angles than no-transcribing ones, indicating that the D-bound F unit follows the motion imposed by D.

**Extended Data Fig. 9:**
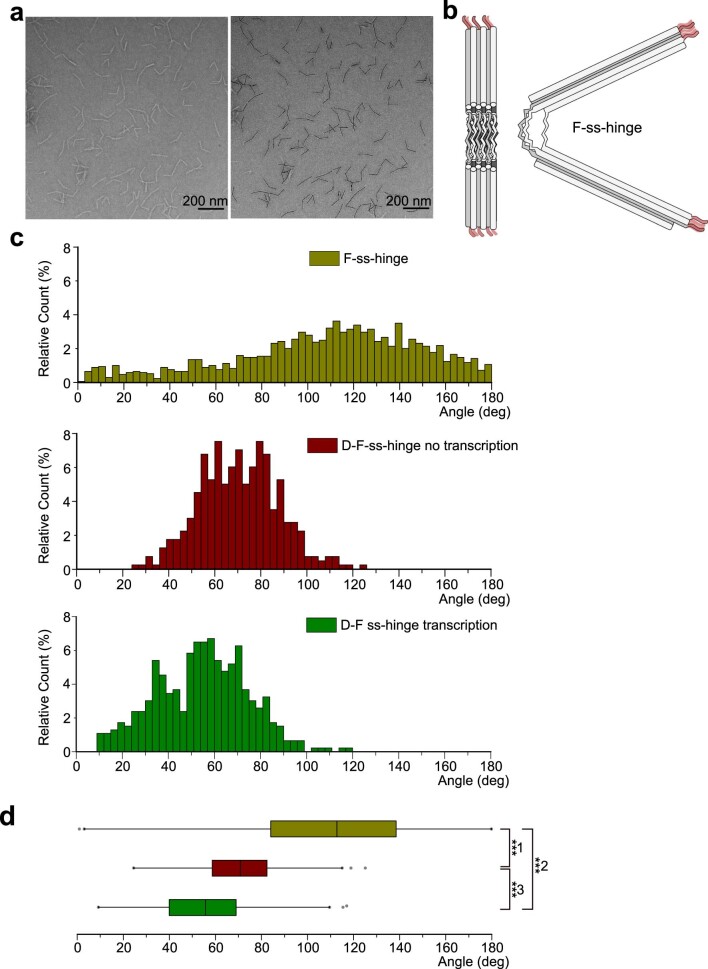
Influence of F-no-hinge and D–F-ss-hinge on angle distribution in D–F. The example of a TEM image for the F-no-hinge structure (**a**) shows how large the angular range is when the double-stranded structure is absent in the flexure region making it impossible to obtain the correct structure that determines the angular shape of the origami structure. The F-no-hinge (**b**) constructs shows a wide and flat distribution (**c**,**d**; F-ss-hinge: olive, n = 1682, 28 micrographs, 107.35° ± 41.03°, min=0.95° max=179.87° median = 112.78°) with obtuse angle. When combining the F-no-hinge origami with the D unit the angle is reduced to 71° ± 17° with a narrower distribution (D–F-ss-hinge No Transcription: wine, n = 398, 21 micrographs, 70.71° ± 17.12°, min=24.52° max=125.08° median = 70.83°). Under transcription conditions, the average distribution angle shifts to 55° ± 20° and becomes slightly larger than expected for the transcription sample (D–F-ss-hinge Transcription: green, n = 462, 84 micrographs, 54.81° ± 19.96° mean and SD, min=9.21° max=117.00° median = 55.63°). **d**) Box-plots show how the distribution for the F-no-hinge (olive) is widely spread over a wide range of angles, having a difference between Q3 and Q1 of 54° while in the D–F this range is reduced to 23° in absence of transcription (wine) and 29° in presence of transcription (green). ***1: p = 4 × 10^−138^, ***2: p = 6 × 10^−229^, ***3: p = 2 × 10^−33^. p-values obtained with two-tailed, heteroscedastic *t*-test. Box-plot edges: 25^th^ and 75^th^ percentiles. Box lines: 50^th^ percentile. Whisker size: 1.5× the IQR, grey dots: outliers. Error ranges: mean and S.D.

This trend becomes even more apparent for a D–F complex in which F has a completely single-stranded hinge region (F-ss-hinge, Extended Data Fig. 9a,b). In this ‘s-hinge’ design the counteraction of the dsDNA flat spring and ssDNA tension sequences is absent. Consequently, F does not assume a defined angle, which is reflected by a broad angle distribution median of 113° (Extended Data Fig. 9c,d, olive, F-ss-hinge). In contrast, when bound to D the median shifts towards a more acute median angle of 71° (Extended Data Fig. 9c,d, wine, D–F-ss-hinge no transcription) under no-transcription conditions, and a median of 56° under transcription conditions (Extended Data Fig. 9c,d, green, D–F-ss-hinge transcription), comparable with the medians obtained for the nNE. The box-plots also show that the angle distributions become considerably narrower in the D–F-ss-hinge complex compared with the F-ss-hinge monomer: under no-transcription conditions the difference between Q3 and Q1 in D–F-ss-hinge measures was only 23°, whereas in the F-ss-hinge it measures 54° (Extended Data Fig. 9d).

**Extended Data Fig. 10:**
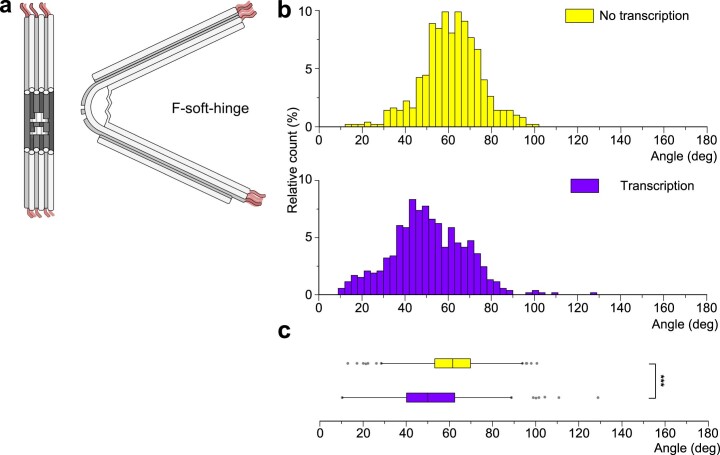
Influence of F-soft hinge on angle distribution in D–F. The change in angle distribution during transcription in the D–F structure is also evident in the presence of F-soft-hinge (**a**) in the complex (**b**, **c**). The D–F-soft-hinge complex shows an average angle of 61° ± 14° (yellow, n = 496, 32 micrographs, min=12.97° max=100.56°  median= 61.60°) in the no transcription case and 51° ± 17° in the transcription sample (violet, n = 528, 78 micrographs, min=10.31° max=128.88° median = 50.08°). (**c**) The box-plot shows how the distribution shifts towards more acute angles in the case of transcription compared to the no transcription sample. ***: p = 2 × 10^−26^, p-values obtained with two-tailed, heteroscedastic *t*-test. Box-plot edges: 25^th^ and 75^th^ percentiles. Box lines: 50^th^ percentile. Whisker size: 1.5× the IQR, grey dots: outliers. Error ranges: mean and S.D.

As a further control, when the D unit was coupled to an F derivative with a soft hinge (F-soft-hinge; Extended Data Fig. 10a), in which two staples are removed from the dsDNA flexure region, the transcription conditions show a behaviour that is comparable to the D–F sample. The median angle of the distribution shifts from 62° in the absence of transcription (Extended Data Fig. 10b,c, yellow) to 50° under transcription conditions (purple).

We next measured the transcription speed of different driver–follower constructs relative to the single D (or nNE) unit. D was combined with the F-ss-hinge, F-soft-hinge or the F unit (Fig. 6h). Although the differences are small, we observed a significant increase in the transcription rate constant for the D–F-ss-hinge and the D–F-soft-hinge of 1.2 ± 0.2 (*P* = 0.001) and 1.2 ± 0.2 (*P* = 0.01), respectively, relative to the nNE. The combination of D with F (D–F) showed no significant increase in transcription rate relative to the nNE (Fig. 6h, nNE versus D–F). These results indicate that the combination of the active driver with a passive follower influences the closing and opening speed of the dimeric system (Supplementary Text 7).

## Conclusion

We describe the bottom-up construction of a biohybrid DNA-origami-based nanomachine that performs tasks fundamental for any device requiring automated motion: an autonomous, fuel-driven, rhythmically pulsing DNA nanoengine that can be coupled as a module with a passive DNA-origami-based ‘follower’ entity to which it transmits its motion and force, thus constituting a driver–follower pair.

Since DNA-origami technology permits modular bottom-up construction of robust nanostructures with diverse properties that span from mechanically rigid to mechanically compliant structures^12,23,24,50^, all allowing for combination into a single architecture, the versatility of mechanical power transmission by the nNE to other devices is high. Although the passive follower structures used here are fairly simple, the prototypical design of D–F suggests that the D should be applicable to other DNA nanostructures as a driving engine to achieve larger and more complex structural rearrangements as exemplified before in non-autonomous systems^51^ (Supplementary Text 8).

For future applications, one may envision introducing a clutch mechanism that allows the driver to be disconnected from one coupled follower and instead connected to another ‘on the fly’ while the engine is still running; for example, by controlling the hybridization of the D–F connecting oligodeoxynucleotides with light-switchable isomers, as demonstrated in other DNA nanomachines^11,52–54^. Similarly, photoresponsive molecules could be introduced into the promoter region^55^ to stop active movement by light even in the presence of fuel (Supplementary Text 9).

## Methods

### Transcription experiments

The samples were prepared to obtain a final concentration of the origami structure of 10 nM with the addition of 2 equiv. HT–T7RNAP. The origami and the fusion protein were premixed in the required ratio with only the addition of transcription buffer (TB) but without diluting the sample to the final concentration, and the sample was incubated for 1 h in ice to favour the combination of the two components. The MB fluorescence was calibrated by incubating known amounts of a complementary oligodeoxynucleotide with the MB to estimate the amount of RNA transcribed within a certain time interval. To be able to follow the transcription, the MB was added in a final concentration of 600 nM with addition of ribonuclease inhibitor (0.38 U µl^−1^ Recombinant RNasin, Promega). To start the transcription 2 mM of NTPs were added to the solution and the sample was diluted and brought to the final concentration in a mixture of 1× nanoengine origami buffer (NEOB) and 1× TB with addition of NaCl to a final concentration of 120 mM. The fluorescence was monitored in a thermocycler over at least 3.5 h at 37 °C (excitation wavelength, 491 nm; emission wavelength, 517 nm). The sample volume loaded for each well is 20 µL for PerkinElmer ProxiPlate-384 F Plus plates or 30 µl for Greiner Fluotrac 200 plates. After each transcription experiment 10 µl of reaction products was run on 6% PAGE to confirm the correct length of the transcription products.

### smFRET studies

Surface-based smFRET experiments were performed on a prism-type total internal reflection fluorescence microscope. The otherwise unaltered nNEs were specifically labelled with one Cy3 donor and one Cy5 acceptor dye by replacing the unlabelled staple strands Hi-MC-62 and Hi-MC-137 for the fluorophore-labelled staple strands Hi-MC-62 ExtCy5 and Hi-MC-137 ExtCy3, respectively (Supplementary Datasets 1 and 2). The fluorophore labelling did not substantially alter the structure of the nNEs (Supplementary Fig. 6).

A prism-type total internal reflection fluorescence set-up built around an Olympus-IX83 microscope, equipped with a 60×, 1.20 numerical aperture water objective and two sCMOS cameras (Hamamatsu, Flash-4 V3) and two different wavelength laser lines, was used to perform all single-molecule fluorescence experiments. Flow cell sample channels were prepared on surface-passivating quartz microscope slides coated with a mixture of 90% methoxy polyethylene glycol succinimidyl valeric acid (m-PEG SVA) and 10% biotin-PEG SVA using previously established protocols^56,57^. A solution containing ~50–200 pM previously formed nNE complex was introduced into the chamber to sparsely coat the PEG surface, resulting in a surface coverage of ~200 molecules per field of view. Excess non-immobilized nNEs were then washed away with 200 μl wash buffer (100 mM Tris–HCl, 120 mM NaCl, 14 mM MgCl_2_ pH 7.5, 25 °C). Average laser powers were optimized to ~300 mW green laser (532 nm, Lase Quantum, Opus-532) for a strong signal-to-noise ratio and limited photobleaching for prolonged observation, and ~600 mW red laser (639 nm, Coherent Genesis MX) to confirm the expected single-step bleaching of the Cy5 as a criterion for trace selection. Movies of 1,000 frames were recorded at 100 ms per frame for each condition with continuous 532 nm excitation to capture FRET excitation) and 639 nm laser during only the last 100 frames to observe Cy5 photobleaching.

To start the leaf-spring nanoengine, purified nNEs were incubated (unless otherwise indicated) with a fivefold molar excess of HT–T7RNAP (1 nM to 5 nM) on ice for 1 h to achieve RNAP loading through the HaloTag (HT). All experiments were performed at ambient temperature (25 °C) in origami transcription buffer (1× OTB = 100 mM Tris–HCl, pH 7.5, 120 mM NaCl, 14 mM MgCl_2_, 1 mM EDTA, 2 mM spermidine). An enzymatic oxygen scavenging system consisting of 44 mM glucose, 2 mM trolox, 165 U ml^−1^ glucose oxidase and 2,170 U ml^−1^ catalase was added to the OTB immediately before imaging. Various concentrations of ultrapure NTP mix (High Purity NTPs, Cytiva) were added where indicated. Typically, three independent biological replicates were performed, and their data were pooled for the analysis. Single-molecule time traces displaying single-step Cy5 bleaching, a signal-to-noise ratio of >1.5, and at least 100 frames of Cy3 and Cy5 signal were extracted using a custom MATLAB code. Additional custom analysis programs in MATLAB were used to derive molecule statistics, and MATLAB scripts and/or Origin Pro-2017 were used to plot the data. Individual time traces were analysed by visual inspection to obtain dwell and transition times, where open (low-FRET, *τ*_o_) and closed (high-FRET, *τ*_c_) dwell times were fit with double-exponential functions, yielding short (*τ*_1_) and long (*τ*_2_) time constants and their respective amplitudes *A*_1_ and *A*_2_ to calculate weighted averages as *A*_1_*τ*_1_ + *A*_2_*τ*_2_. Gamma distribution functions of the form (Δ*t*)^*N*−1^exp(−*k*Δ*t*) were instead used to fit the mechanistically more complex transition times from low- to high-FRET and back, *τ*_t-C_ and *τ*_t-O_, respectively.

### NTP concentration toggling smFRET experiments

For ‘NTP-switch’ experiments, surface-immobilized nNE molecules were first exposed to OTB supplemented with an oxygen scavenging system (OSS) in the absence of NTPs and monitored for 10 s. The buffer was then exchanged with fresh OTB solution containing OSS and 5 mM of each NTP over 110 s of dark phase (laser excitation off) (Fig. 4g, grey segment). The dark period allowed time for a homogeneous exchange of buffer and for the OSS to reduce photobleaching probability. After the dark period, the nNEs in the same field of view were observed in real time for another 40 s. In a second round of buffer exchange in the dark, all NTPs were washed out with at least 10 volumes of OTB without NTPs, followed by a final wash with OTB containing OSS but no NTPs. Finally, the same field of view was monitored for another 20 s, with representative nNE time traces shown in Fig. 4g.

### MD simulations

Multiple designs of the nanoengine were simulated using the oxDNA coarse-grained model for DNA origami. OxDNA is described in detail elsewhere^40–43^, but briefly, it is an empirically derived force field designed with DNA nanostructures in mind. The model has been shown to reproduce structural, kinetic and thermodynamic properties of DNA, including persistence length, strand displacement rates and free-energy barriers between states, with reasonably high accuracy, while still being sufficiently coarse-grained to allow simulations of DNA origami at timescales of up to milliseconds^58,59^. Equilibrium simulations of the nanoengine were carried out for four different designs: nNE, nanoengine, nanoengine_NB and nNE_NB where ‘NB’ structures had the dsDNA-t deleted prior to simulation.

Starting configurations were obtained by exporting the cadnano^60^ design file into oxDNA format using the TacoxDNA converter^61^ in oxView^62,63^. Rigid body dynamics in oxView were used to bend the arms into a rough initial configuration. Relaxation was then performed using the method described previously^64^. After relaxation, the dsDNA template was built using oxView’s editing tools and a further round of relaxation performed. After the average energy per particle stabilized around −1.5 simulation units, the structures were equilibrated with production conditions for a further 2.5 × 10^8^ simulation steps to allow equilibration of the angle distribution.

Equilibrium simulations were carried out for 10^9^ oxDNA simulation steps with an integration timestep of 0.003, and a temperature of either 23 °C or 37 °C was imposed using an Andersen-like thermostat. Configuration snapshots were saved for analysis every 5 × 10^5^ steps, giving 2,000 configurations per simulation which were verified to have well-decorrelated angle distributions. To identify the effects of secondary structure in the flexure region, a second set of equilibrium simulations (no structure, NS) was performed with the same parameters as before; however, the base type of the nucleotides in the single-stranded region of the flexure were changed such that they could not form base pairs.

Closing rates were measured by running ten replicates of simulations with a constant force of 16 pN (based on the typical value of tension exerted by a polymerase on a duplex DNA^65^) applied between the nucleotide where HT–T7RNAP is covalently linked to the first nucleotide in the first stop sequence in the dsDNA-t with a cut-off radius of T7RNAP (7.5 nm) (ref. ^45^). The simulations were started from the final configuration of one replicate of the associated equilibrium simulation and run for 10^7^ steps with snapshots saved every 5,000 steps for a total of 2,000 configurations per simulation. Other parameters were the same as in the equilibrium simulations. One additional simulation was performed for each design where the force was applied for 10^9^ steps to allow the structure to equilibrate in the closed position. Relative size calculations of T7RNAP were based on PDB 3E2E (ref. ^45^).

Opening rates were measured by running ten replicates starting from the final configuration of the equilibrated pulling simulation. Each simulation was run for 2 × 10^7^ steps with snapshots saved every 5,000 steps for a total of 4,000 configurations per simulation. In addition to the full nanoengine, simulations were also performed for NTS and NTS-NS, allowing the structure to open under only the influence of the flexure. Other parameters were the same as in the equilibrium simulations.

Simulations were aligned and mean structures obtained from the equilibrium simulations using oxdna_analysis_tools^63^ and movies of the trajectories were produced using oxView^63^. At equilibrium, the structure demonstrates random thermal fluctuations that can be related to the spring constant of the structure

To obtain spring constants and opening/closing rates, linear regression was performed on the point clouds corresponding to the arms of each hinge and the angle between the arms calculated for each snapshot. Spring constants were then estimated using the equipartition theorem for a simple harmonic oscillator:

$$\frac{1}{2}k\overline{{\left(\theta -{\theta }_{0}\right)}^{2}}=\frac{1}{2}{k}_{{\rm{B}}}T$$

where *k* is the torsional spring constant, *θ* and *θ*_0_ are the per-frame angular displacement and the average position, and *k*_B_*T* is the thermal energy.

Opening and closing rates were calculated using a linear regression of the angle traces over the simulations. Because it is difficult to establish real-time correspondence of events in coarse-grained simulations, all rates were normalized to the mean rate of the corresponding nNE simulation. The significance of distributions was determined using a two-tailed Kolmogorov–Smirnov test.

### TEM

To determine angle distributions, TEM images of the different origami structures were collected. The samples were diluted to a concentration of 1 nM and 3 μl was applied to a TEM grid (Cu 3 mm 400 MESH TEM GRID, SPI-GIDS) coated with a 5 nm continuous carbon film prepared previously with a Leica EM ACE600. Prior to the application of the sample the TEM grid was glow-discharged in a PELCO easiGLOW (Ted Pella) for 45 s at a current of 15 mA. Samples were incubated on the grid for 20 s and excess liquid was removed by filter paper absorption. Samples were stained by applying 3 μl of a 2% uranyl formate solution, which was immediately removed with filter paper, followed by addition of another 3 μl of a 2% uranyl formate solution and a 20 s incubation before the excess stain was removed by filter paper absorption and the grid allowed to dry completely. Images were taken in low-dose mode using a FEI Tecnai Spirit Bio-Twin Microscope at 120 kV equipped with a Gatan US4000 4k × 4k charge-coupled device camera. The magnification used was 30,000× (pixel size, 3.80 Å per pixel) for angle distributions and 68 kx (pixel size 1.66 Å per pixel) for detailed figures. The images were collected using DigitalMicrograph (Gatan) and stored in .mrc or .dm4 format. For transcription and non-transcription conditions, the origamis were treated as described in the Transcription experiments section, omitting the addition of NTPs for the non-transcription samples. Before imaging, all samples were incubated for 1.5–4 h at 37 °C.

### 2D class averaging

RELION v.4.0-beta-2 was used for 2D classification^66^. The program’s reference-free 2D classification finds a maximum a posteriori that leads to clear images of rigid features but also blurring and loss of information of flexible features, producing average structures of the most abundant angles. Data were converted, when necessary, from .dm4 format using IMOD v.4.10.51 with dm2mrc (ref. ^67^) and from tif format with the tif2mrc command, to mrc files. Contrast transfer function estimation was performed with ctffind 4.1.1.3 incorporated in RELION^68,69^. Two 2D-classification runs were performed: the first classification was performed on particles extracted from a manual picking job, with a box size of 540 pixels. The classification was performed by requiring 30 classes with a regularization parameter *T* value of 2 and 30 iterations. The circular mask used for classification was set at 1,300 Å. The in-plane angular sampling was 2 and a wide offset search of 15 pixels was used with an offset search step of 1 pixel. Classes with distinguishable features were selected, centred and extracted with a box size of 384 pixels. A second round of 2D classification was performed with 20 classes and a regularization parameter *T* value of 10 and 30 iterations. The angular search was kept at 2°, while the offset search range was reduced to 3 pixels for the second classification cycle. The images of the 2D averages were exported from RELION in png format. The size and angles were measured in Fiji^70^ and for size estimation the known size of the box was used for calibration and pixel size determination.

### Assembly of the D–F system

We added five unique ssDNA overhangs to the axial extremities of each origami arm of both D and F (Fig. 6a–c, pink). Each of these in total ten ssDNA sequences in D allow for the hybridization of only the complementary ten ssDNA sequences on F to form the rhomboid-shaped heterodimer D–F (Fig. 6c–e); formation of the homodimers D–D or F–F is impeded due to the non-complementarity of the sequences (Supplementary Fig. 5a,b). To strengthen the connection between D and F, three LNA modifications^71^ were included in two of the overhanging sequences on each D arm that connect to F (Supplementary Fig. 5b, red). The purified driver and follower origami were combined equimolarly without further dilution and incubated at 30 °C over 2 h to favour the combination of the two structures. Typical concentrations of the driver and follower solutions were 300–400 nM, resulting in an estimated concentration of the D–F system of 150–200 nM. After the first incubation the samples were subsequently diluted for further application.

### Angle measurement from TEM micrographs

To determine the angle distribution several TEM images were examined using the open-source imaging software Fiji (imagej.net/software/fiji/), a package distribution of ImageJ2. The Angle Tool was used to measure the angle formed by the two stiff arms of a single origami structure. The contrast differences produced by the DNA helices, which are visible due to differences in the staining density of the origami matrix, were used as a guide for the alignment of the line from the Angle Tool. All structures with a clearly visible, correct and complete structure were measured, with no distinction made based on visible angle. Structures that were broken, showed signs of missing parts, or were not clearly flat on the surface were ignored in the angle measurements to ensure that only intact origami were measured. In case of the D–F system, only constructs that clearly showed the connected origami structures were considered for the angle measurement. The angles of the D and F units were measured independently for each duplex structure. To avoid operator bias during the measurements, all micrographs observed were labelled with a letter and number code, and only after the measurement was the letter–number code associated with the corresponding sample. TEM micrographs were obtained from at least two independent experimental sets for each sample.

### Reporting summary

Further information on research design is available in the Nature Portfolio Reporting Summary linked to this article.

## Online content

Any methods, additional references, Nature Portfolio reporting summaries, source data, extended data, supplementary information, acknowledgements, peer review information; details of author contributions and competing interests; and statements of data and code availability are available at 10.1038/s41565-023-01516-x.

### Supplementary information

Supplementary Information Supplementary Chapters 1 and 2, Methods, Fig. 1–6, Table 1 and Texts 1–9.

Reporting Summary

Supplementary Video 1 Video cartoon showing the closing and opening of the nanoengine.

Supplementary Dataset S1 Detailed protocol and procedure for the assembly and purification of the DNA nanoengine origamis and complete list of all ODNs used for the DNA nanoengine origamis.

Supplementary Dataset S2 Graphical representation of the CadNano maps of the nanoengine origami.

Supplementary Dataset S3 Glossary and detailed description of the various constructs I–X used in this study.

Supplementary Dataset S4 Source data for Figs. 2a–d, 3c,d, 6f–h and Extended Data Figs. 3a, 6d,e, 8b,c, 9c,d, 10b,c.

Supplementary Dataset S5 Source data on quantification of proper formation of D–F duplexes based on TEM micrographs.

### Source data

Source Data Fig. 1, Source Data Supplementary Fig. 4b and Extended Data Figs. 2 and 3. Unprocessed AFM scan of Fig. 1f, unprocessed TEM micrograph of Fig. 1g,i,j, unprocessed TEM micrograph of Supplementary Fig. 4b, unprocessed AFM scan of Extended Data Fig. 2a, unprocessed TEM micrograph of Extended Data Fig. 3b

## Data Availability

All data generated or analysed during this study are either included in this published article (and its Supplementary Information files) or are available as follows: data for smFRET analysis, http://deepblue.lib.umich.edu/data/concern/data_sets/474299762; original design file and the edited oxDNA structures used to start the simulations are found in the Nanobase repository, https://nanobase.org/structure/196; all generated simulation trajectories, https://zenodo.org/record/8248808. Source data are provided with this paper.

## Code availability

Codes and algorithms for the smFRET analysis can be accessed without restrictions at https://github.com/walterlab-um/rhythmically pulsing-leaf-spring_SMCodes. The processed MD simulation data used to generate the figures, and the analysis and plotting code are available at https://github.com/sulcgroup/hinges.
