# Supplementary material for: A rhythmically pulsing leaf-spring DNA-origami nanoengine that drives a passive follower
Source: Nat Nanotechnol. 2023 Oct 19;19(2):226–36. doi: 10.1038/s41565-023-01516-x (PMC10873200; doi:10.1038/s41565-023-01516-x)
Supplement: Supplementary file 1 — Supplementary Chapters 1 and 2, Methods, Fig. 1–6, Table 1 and Texts 1–9. [file 41565_2023_1516_MOESM1_ESM.pdf]

nature nanotechnology

Article https://doi.org/10.1038/s41565-023-01516-x

A rhythmically pulsing leaf-spring DNA-origami nanoengine that drives a passive follower

In the format provided by the authors and unedited

Table of Contents

**Table of contents**	1
**Supplementary Chapter 1**	2
**Supplementary Chapter 2**	3
**Supplementary Methods**	6
Buffer Systems	6
ODNs	7
DNA origami design	7
Assembly of the DNA origami	8
DNA origami purification	8
Expression and purification of HT-T7RNAP fusion protein	9
Assembly of the dsDNA-t	9
Testing Chloroalkane DNA connection to the HT enzyme	10
AFM imaging	10
MB signal calibration	11
Chloroalkane ODN competition experiments	11
MD trajectory video links	11
Evaluation of the yield of Driver-Follower duplex formation	12
Gel electrophoresis	12
Statistics and Reproducibility	12
**Supplementary Figures S1-S6**	14
Suppl. Fig. S1	14
Suppl. Fig. S2	15
Suppl. Fig. S3	16
Suppl. Fig. S4	17
Suppl. Fig. S5	18
Suppl. Fig. S6	18
**Supplementary Table S1**	19
**Supplementary Text**	20
Suppl. Text 1	20
Suppl. Text 2	20
Suppl. Text 3	20
Suppl. Text 4	21
Suppl. Text 5	21
Suppl. Text 6	21
Suppl. Text 7	21
Suppl. Text 8	22
Suppl. Text 9	22
**Supplementary References**	22

Supplementary Chapter 1

Design of a rhythmically pulsing leaf-spring DNA nanoengine

Two straight and rigid 60 nm long origami arms are formed by a honeycomb lattice of 18 helix bundles (18hb, **Fig. 1a**, **Suppl. Dataset S1**, **Suppl. Dataset S2**, **Nanobase**[1] entry https://nanobase.org/structure/196). They are connected by six 28 nm long dsDNA helices arranged as a 12 nm wide sheet that can bend to serve as the compliant leaf-spring, as shown in previous work[2,3], and by six single-stranded (ss)DNA strands that ensure the formation of the bent V-shape by being overall shorter than the double-stranded compliant component (**Fig. 1 a-e**; **Ext. Data Fig. S1a**). A 154 nucleotide (nt) long transcribable double-stranded DNA template (dsDNA-t) strand (**Fig. 1a-c**, **Ext. Data Fig. S1a**,**b**) spans the origami arms and is firmly connected to each of them at 30 nm distance from the leaf-spring ends (**Fig. 1a-c**; **Fig. 1e**, red dots). One of the origami arms contains a sequence with a 5'-chloroalkyl group attached near the dsDNA-t (**Fig. 1e**, yellow dot; **Ext. Data Fig. S1c**), to which a HaloTag (HT)-T7RNAP fusion protein (**Fig. 1a-c**, orange-blue; **Ext. Data Fig. S1b,d,e**) is covalently coupled through its HT subunit[4] (**Ext. Data Fig. S1d-f**), allowing its use to exert traction on the dsDNA-t under transcription conditions[5-7]. The dsDNA-t contains a T7RNAP-promoter region (yellow, **Ext. Data Fig. S1a,b**) and a sequence that, once transcribed, binds a molecular beacon (MB) (green, **Ext. Data Figs. S1a,b,g**) that monitors the amount of RNA generated during transcription. The relative proximity of the HT-attachment site and the dsDNA-t (**Fig. 1e**, yellow and red dots) helps the HT-T7RNAP capture the T7 promoter sequence in the dsDNA-t (**Ext. Data Fig. S1a,b**, yellow) and facilitates initiation of transcription in the presence of NTPs. Once the T7RNAP reaches the terminator sequence at the opposite end of the dsDNA-t (**Ext. Data Fig. S1a,b**, red) the sequence is released by the polymerase. The opposing origami arm contains four biotin residues that protrude at the outer part of the structure, to which four streptavidin protein molecules can be attached to unambiguously identify each arm by atomic force microscopy (AFM, **Fig. 1d**,**f**) or transmission electron microscopy (TEM, **Fig. 1g**). The biotin residues can also serve as attachment points to streptavidin-coated surfaces or to other origamis to form larger bio-hybrid complexes.

These features were designed to operate the nanoengine autonomously and continuously in a repeating opening/closing cycle (**Fig. 1h**, **Suppl. Movie 1**), starting from the "open" conformation of the structure, in which the T7 promoter is bound by the HT-T7RNAP to begin transcription (**Fig. 1h**, **1**). The pulling of the dsDNA-t through the immobilized HT-T7RNAP closes the origami structure while building up spring tension in the compliant segment of the structure that generates a counteracting force (**Fig. 1h**, **2**). Once the terminator sequence has been reached, the polymerase releases the dsDNA-t so that the structure opens to its equilibrium conformation, discharging spring tension (**Fig. 1h**, **3**), and is reset to begin a new cycle.

The purity of the nanoengine was validated by gel electrophoresis (**Suppl. Fig. S1a**,**b**) and its structural integrity confirmed by AFM (**Fig. 1f**, **Ext. Data Fig. S2a-c**), and TEM (**Fig. 1g, Ext. Data Fig. 2d**). In the AFM images of nanoengines that are not engaged in transcription the dsDNA-t is visible between the origami arms. Height profiling confirms the expected distance of 21 nm between the streptavidin tags and the height of the origami arms (**Ext. Data Fig. S2a-c**). The TEM images additionally show the HT-T7RNAP (**Fig, 1g**, right panel, blue arrow) and biotinylated positions occupied by streptavidin (green arrows). 2D-averaging on the negatively stained TEM micrographs improved the signal-to-noise ratio of the expected V-shaped origami leaf-spring structure (**Ext. Data Fig. S2d**) and further confirms multiple structural features of the DNA origami (**Suppl. Text 1**).

Analysis of nanoengines during transcription by TEM (**Fig. 1i,j**) indicates that the dsDNA-t, designed to be relaxed in the origami's open position, exists in a stretched state during the initial phase of transcription (**Fig. 1i**, right), as shown in the 3D model (**Fig. 1i**, left). Examples of nanoengines in an advanced phase of transcription show the origami arms in a "closed" state (**Fig. 1j**, right), as depicted in the 3D model (left).

Supplementary Chapter 2

MD simulations define nicked-nanoengine features governing opening/closing rates

Coarse-grained molecular dynamics (MD) simulations using the oxDNA model[8-11] of different nanoengine designs were performed to further characterize the impact of our design choices on mechanical properties (**Fig. 5a**). Notably, our simulations showed a

large influence arising from sequence-dependent secondary structures formed transiently by the ssDNA regions of the hinge. Disallowing formation of these secondary structures by disabling base pairing in those regions ("no structure": all structures labelled as NS) increases both the open structure equilibrium angle and the stiffness of the structures (**Fig. 5b**, **Ext. Data Fig. 6a**). Conversely, allowing the formation of secondary structures reduces the equilibrium angle, independent of variations in the dsDNA-t (NTS, NE, and nNE in **Fig 5b**). This effect is particularly pronounced in the NTS, where the difference in equilibrium angle between NTS and NTS_NS is ~25°, suggesting that the secondary structures in the hinge region are the largest determinant of equilibrium angle and stiffness (**Ext. Data Fig. S6a**).

In the absence of the dsDNA-t and secondary structures in the hinge region, the DNA leaf spring can stretch out and reach a mean equilibrium angle of 80°±9° (S.D.). When dsDNA-t is reintroduced, nanoengine_NS and nicked-nanoengine_NS have comparable equilibrium angles of 71±6° and 74±5°, respectively, both with a negatively skewed distribution caused by the dsDNA-t-imposed upper limit. When secondary structures in the ssDNA hinge regions are permitted NTS, nicked-nanoengine, and nanoengine have highly comparable equilibrium angles of 55°±8°, 58°±7°, and 54°±7°, respectively (**Fig. 5b**), implying that the effect of the dsDNA-t on the equilibrium angle is negligible. The structures in the hinge are predicted to only slightly be affected by temperature changes as increasing the simulation temperature from 23 °C to 37 °C only marginally increases the hinge angles observed in the simulations. (**Ext. Data Fig. S6b**).

We next determined approximate spring constants for the leaf-spring of the various nanoengine-structures (**Suppl. Methods**). For nanoengine, nicked-nanoengine, and NTS, the spring constants lie in the range of 200-300 pN×nm×rad^{-1}. For structures where the dsDNA-t impedes opening, such as nanoengine_NS and nicked-nanoengine_NS, the spring constant range is much higher, 350-550 pN×nm×rad^{-1}. For the freely fluctuating NTS_NS, the estimated spring constant is 190±22.3 (S.D.) pN×nm×rad^{-1} (**Ext. Data Fig. S6c**). These values are similar to those previously measured from TEM imaging of a similar structure (107-367 pN×nm×rad^{-1})[2].

To test whether these predictions are consistent with our experimental results, we used TEM to compare the angle distributions of NTS with that of nicked-nanoengine

(**Ext. Data Fig. S6d,e**). The two populations have a similar distribution (p=0.6), suggesting that the simulation-predicted secondary structures are also relevant experimentally and have an impact on the structures' conformations. Notably, the angle was systematically lower in the simulations compared to the experiment. A possible explanation for this difference is that angles were measured on surface-deposited structures, which may cause a slight deformation of the structure due to adherence to the TEM grid. In both 3D simulations and 2D-class averages of top-views obtained from TEM micrographs, we observed out-of-plane twisting of the structure (**Ext. Data Fig. S6f**). The averages show one well-defined arm with a width of 12 nm and a length of 60 nm, as per the design, while the second arm is always tilted to one side (**Ext. Data Fig. S6g**), suggesting that deposition of nanoengines on the grid surface may reduce some of this twist by slightly opening the structure.

To sample the behaviour of the nanoengine under tension mimicking the action of HT-T7RNAP, simulations of out-of-equilibrium pulling were performed by applying a constant force comparable to that exerted by the polymerase between the nucleotide covalently linked to the T7RNAP and the first nucleotide of the terminator sequence (16 pN)[12]. Release simulations were then performed by taking the final configurations of (nicked-)nanoengine, (nicked-)nanoengine_NS, NTS, and NTS_NS equilibrated at 16 pN force (**Suppl. Text 4**). While the pull rates were similar for different structures (**Fig 5c**), there were large differences in the re-opening rates when the force was released (**Fig 5d**). For the NS simulations, rates were 1.5 times faster than for the corresponding simulation where the secondary structures were allowed to form, suggesting that the closed state is stabilized by the secondary structures, which impede the transition from the closed to the open state. This finding explains the results from **Ext. Data Fig. 3a**: the advantage gained by the nicked-nanoengine_soft (nNE_{soft}) in the closing phase due to lower resistance is diminished by slower opening, as the nNE_{soft} spring has to counteract the secondary structures. Furthermore, these simulations indicate that the experimentally observed difference between nanoengine and nicked-nanoengine is likely due to the supercoiling and topological stress caused by T7RNAP-activity rather than the mechanical properties of the origami (**Ext. Data Fig. S7a**,**b**, **Suppl. Text 5**).

To better understand the observed reduction in transcription rate when the dsDNA-t is anchored next to HT-T7RNAP only (**Fig. 2d**), we extracted the fraction of

time the promoter region spends next to the polymerase when the dsDNA-t is fully anchored at both ends to the origami versus being attached next to the polymerase only (**Ext. Data Fig. S7c**). Since T7RNAP is not explicitly represented in our simulations, an approximation of accessibility was made based on the dimensions of a T7RNAP crystal structure (PDB ID: 3E2E), in which the enzyme has approximately the geometry of an 8.6 nm diameter sphere[13] (**Ext. Data Fig. S7d**, **Suppl. Text 6**).

Supplementary Methods

Buffer systems

1× TAE buffer: 40 mM Tris·HCl, 20 mM AcOH, 1 mM EDTA at pH 8.3.

1× DNA buffer: 10 mM Tris·HCl, 50 mM NaCl, 10 mM $MgCl_2$ at pH 7.5.

1× origami buffer (NEOB): 5 mM Tris, 5 mM NaCl, 5 mM EDTA, 14 mM $MgCl_2$.

1× ligase buffer: 40 mM Tris·HCl, 10 mM $MgCl_2$, 10 mM dithiothreitol (DTT), 5 mM ATP at pH 7.8.

2× Precipitation Buffer (5% PEG 8000 (wt/vol) 5 mM Tris, 1 mM EDTA and 505 mM NaCl

5× transcription buffer (TB): 200 mM Tris·HCl (pH 7.9 at 25 °C), 50 mM DTT, 50 mM NaCl and 10 mM spermidine.

1× lysis buffer: 50 mM Tris, pH 7.8 at 4 °C, 300 mM NaCl, 10% glycerol, 20 mM imidazole and 1 mM $ZnCl_2$

1× storage buffer: 50 mM Tris, pH 8.0, 0.1 mM EDTA, 300 mM NaCl and 50 % (vol/vol) glycerol

1× wash buffer: 100 mM Tris-HCl, 120 mM NaCl, 14 mM $MgCl_2$ pH 7.5 at 25 °C

ODNs. Oligodeoxynucleotides (ODN) staples used for the assembly of the DNA origami nanoengine structure were purchased from Eurofins Genomics (high-purity salt free). The M13mp18 (type 7249) scaffold used for the origami construction was purchased from Tilibit nanosystems GmbH. Chloroalkane-modified ODN (HaloTag Ligand O2 from the Biomers catalog) was purchased from Biomers as HPLC purified. ODNs with fluorophores (Cy3 and Cy5) or biotin as well as ODNs used for the transcribable DNA strand were obtained from Biomers and Microsynth as HPLC purified in lyophilized state. LNA modified ODNs were purchased from Microsynth as HPLC purified ODNs. Upon arrival all ODNs were dissolved in MQ H_2O to obtain a 100 µM solution by following manufacturer specifications. The 2'-O-Methyl RNA Molecular Beacon was purchased from Microsynth in HPLC purified and lyophilized form, dissolved in MQ H_2O to obtain a 100 µM solution by following manufacturer specifications that then was further diluted into 50 µM aliquots (20 µl) and stored at -20 °C, shielded from light.

DNA origami design. The origami was designed around the 7249 nucleotides long M13mp18 circular ssDNA scaffold using the DNA origami design software cadnano2 (https://cadnano.org). Staples were designed to be approximately 42 nucleotides long and when possible optimized to follow design strategies presented in previous publications describing $MgCl_2$-free DNA origami assemblies[14]. Three-dimensional representation based on the cadnano2 designs was obtained with the online Tool CanDo (https://cando-dna-origami.org/). The provided rmsf 3D maps were used for maximizing the overall origami stability. To ensure a high rigidity, the design of the origami arms included a 60 nm long 18 helix-bundle in a honeycomb lattice arrangement. To permit movement of the origami arms, a compliant region was inserted between the two arms, as inspired by others[2], consisting of a 6 helix DNA “sheet” 84 nucleotides long. The flexible double-stranded region was flanked by 6 single-stranded sequences that are part of the scaffold. To achieve the bend in the origami structure these 6 sequences were designed to be 53 nucleotides long so that they are shorter than the compliant dsDNA sequence, creating a tension in the hinge region that causes the origami to take the desired angulated shape. Atomic models were created from the optimized designs using CanDo, which were used to confirm the shape and dimension of the designed structure, refine

the origami, and identify positions for the introduction of modifications such as fluorophores, dsDNA-t, the HT-T7RNAP anchor point, or biotin. To introduce the chloroalkane modified staple, that serves as attachment point for the HT-T7RNAP, and the dsDNA-t, we selected the more stable part of the origami that corresponds to the stiff origami arms at half of their length. To ensure the overall stability of the origami, we modified its basic structure by selecting only staples that were already nicked and are in the required positions whenever possible. To ensure that the functionalized portions of the staples protruded as perpendicularly as possible to the origami surface, the adjacent staple was designed to protrude from the origami core and form a complementary duplex stem area with the staple carrying the functionalization. The double-stranded sequences that protrude from the origami ensures that the sequence is orthogonal to the origami and extends away from the surface as long as the sequence is much shorter than the persistence length of DNA. Biotinylated ODNs were introduced to the outside face of the origami in a tripod-like conformation to increase the upright stability of the origami when anchored to surfaces via streptavidin (sequences: **Suppl. Dataset S1**, cadnano-design: **Suppl. Dataset S2**).

Assembly of the DNA origami. The origami was assembled by combining 13.3 nM of the M13mp18 scaffold with 10 equivalents of each staple and 5 equivalents of the dsDNA template in 1× NEOB. The mixture was divided into 50 µL aliquots into 500 µL reaction tubes and the origami annealed in a thermocycler. The aliquotation was performed to ensure that the samples were fully immerged into the thermoregulating element of the thermocycler. Driver and Follower origami were assembled by adding twice the amount of the connecting ODN compared to the other staples (for sequences and details for the different structures see **Suppl. Datasets S1-S3** and **Ext. Data Fig. S1a,b**).

DNA Origami purification. Structures were purified by precipitation of the origami structure in PEG containing buffer. The aliquoted origamis were combined in one 1.5 ml reaction tube and mixed 1:1 with the 2× Precipitation buffer. The samples were spun at 16000 rcf for 30 min at 20 °C. The supernatant was removed with a pipette by making sure not to touch the pellet. Excess precipitation buffer was removed with thin strips of Whatman filter paper, always being careful not to disturb the pellet. To the pellet 1×

NEOB is added. The pellet was suspended in 5 µL of 1× NEOB for every 100 µL of assembly mixture. The samples were placed into an Eppendorf ThermoMixer C and shaken at 1500 rpm at 25 °C for at least 2h to fully resuspend the sample. Successful purification was confirmed by running 0.3 µL of resuspended sample on 1% agarose gel.

Expression and purification of HT-T7RNAP fusion protein. The HT-T7RNAP fusion protein was expressed and purified following a similar protocol used previously to express and purify the T7RNAP-Zif fusion protein[6,15] The differences in the procedure is the change of the expression plasmid in the *Escherichia coli* (strain BL21 DE3) with the plasmid pQE80HT-HaloTag-T7 RNAP (H= 6×HisTag, T=TEV site (tobacco etxh virus cleavage site), HaloTag=297 AA HaloTag protein tag, T7RNAP= 883 AA T7 RNA polymerase. In contrast to the already published protocol the TEV cleavage step is omitted. In brief the protein was expressed by starting from an overnight preculture at 37 °C in lysogeny broth medium with 50 µg/ml kanamycin. The preculture was diluted to an optical density at 600 nm (OD600) of 0.4 and when cells reached mid log phase of 0.6 (OD 600) expression was induced with addition of isopropyl β-D-1-thiogalactopyranoside (IPTG) to a final concentration of 0.5 mM and followed by incubation at 37 °C for 4h. Cells were collected by centrifugation at 4,000 rcf. The cell pellet was resuspended in 15 ml lysis buffer (50 mM Tris, pH 7.8 at 4 °C, 300 mM NaCl, 10% glycerol, 20 mM imidazole and 1 mM $ZnCl_2$) and subsequently the cells have been disrupted with a French press (1000 psi max, two rounds), spun for 20 min 48,000 rcf at 4 °C and incubated with 1.5 ml of Ni-NTA agarose equilibrated bead matrix (Macherey Nagel) for 30 min and washed three times with the lysis buffer. The fusion protein was purified by affinity chromatography, then washed twice and eluted with elution buffer (lysis buffer containing 250 mM imidazole). The final purification step involved size exclusion chromatography (SEC) of the sample using a Superdex 200 column (GE Healthcare Life Sciences), fractions were collected, and the buffer was exchanged from dialysis buffer (Slide-A-Lyzer Dialysis Cassettes, 10K molecular weight cutoff) to storage buffer (50 mM Tris, pH 8.0, 0.1 mM EDTA, 300 mM NaCl and 50 % [vol/vol] glycerol).

Assembly of the dsDNA-t. The dsDNA-t sequences were assembled from a subset of shorter 5’ phosphorylated ODNs (sequences can be found in **Suppl. Fig. S3a-d** and

Suppl. Dataset S1) that were designed to be partially complementary and overlapping so that they can be annealed in a temperature gradient and subsequentially ligated. The ODNs for the respective sequence were mixed in 5 µM concentration in 1× ligase buffer with addition of NaCl in a final concentration of 20 mM. The samples were heated to 95 °C for 1 min to denature secondary structures and then annealed in a thermocycler with a temperature gradient from 60 °C to 15 °C during 75 min. After the annealing process Ligase (2 µl/150 µl, 10 U) was added and the sample was ligated overnight at 15 °C. The ligated product was separated from the unligated ODNs and the ligase mixture utilizing Amicon Ultra 100 kDa size exclusion filter by spinning at 5000 rcf for 5 min. The buffer was exchanged to 1× DNA buffer by subsequentially spinning for 5 more times by adding 500 µl of 1× DNA buffer each time. Samples were recovered by inverting the filter tubes into an empty reaction tube and spinning for 10 min at 3000 rcf. Successful assembly and purification were verified via 6% PAGE. Concentration was determined by measuring the absorbance at 260 nm.

Testing Chloroalkane DNA connection to the HT enzyme. The chloroalkane modified ODN was either treated alone or in combination with the HT-T7RNAP fusion protein or the HaloTag enzyme in a ratio of 1:3 of DNA to enzyme. The samples were prepared to have a final DNA concentration of 100 nM in 1× NEOB buffer in 20 µL and incubated at 4 °C overnight. For the EDTA samples, the EDTA solution was added after the incubation at 4 °C overnight and the samples incubated for 30 min at 40 °C before 10 µL of each sample were loaded on native 6% PAGE.

AFM imaging. atomic force microscopy was performed on a JPK NANOWIZARD 3. Scanning was performed with ACTA-50 SPM probes, made out of Si (N-type) with 0.01-0.025 ohm/cm. The used cantilevers are 125 µm long, 30 µm wide and 4 µm tall with a f: 200-400 kHz resonance frequency and a spring constant k: 13-77 N/m and an aluminum (Al) coating on the reflex side. Samples were prepared by diluting the samples to 10 nM concertation in 1× origami buffer. 2 µL of the samples were placed on freshly cleaved mica inside a circle (3 mm in diameter) drawn with a thin tip marker pen to spatially confine the droplet and to easily find the deposition area afterwards. The sample is incubated on the surface for 2 min and then washed three times by slowly dripping 200 µL of MQ H_2O onto the mica surface. The surface was dried with a gentle

airstream and the samples imaged in intermitting contact mode on 1–2 µm squares with a 512×512-pixel resolution.

MB signal calibration. To estimate the amount of the transcript produced during the transcription experiments we calibrated the system using known amounts of an ODN (NLS 2, detailed sequence in **Suppl. Dataset S1**) complementary to the MB sequence. The ODN was combined with the MB in the same buffer composition as for the transcription experiments at different concentrations of the complementary ODN. The sample was then treated as the transcription samples and the fluorescence was recorded over time. The obtained fluorescence signals were then used to generate a linear regression fit that was then used to estimate the amount of transcript generated during the transcription runs.

Chloroalkane ODN competition experiments. To test the competition due to the chloroalkane linker we tested preincubating the HT-T7RNAP equimolarly with the chloroalkane ODN for 1h on ice before adding it to the origami. To test if the chloroalkane ODN can displace the HT-T7RNAP from the origami we assemble the nanoengine as described in the "Transcription experiments" section and after the preincubation on ice of the nanoengine with the HT-T7RNAP we added 1, 2, or 5 equivalents of the chloroalkane ODN to the origami solution and incubated for additionally 1h on ice. We then proceeded to finish the sample preparation for transcription assays as described in the "Transcription experiments" section.

MD trajectory video links. The following links lead to molecular dynamics simulation trajectory videos:
nicked-nanoengine closing/opening:
https://drive.google.com/file/d/1s5jOBrQLVoIsof-mzji9JUFNe6wlepkxl/view?usp=share_link,
nicked-nanoengine_NS closing/opening:
https://drive.google.com/file/d/1wmWeRFPUYUkgFDEPK2YztsVvGz-9WpPf/view?usp=share_link

Evaluation of the yield of Driver-Follower duplex formation. To obtain an estimate of the yield of driver-follower duplex formation, we evaluated 199 TEM micrographs. We counted each correctly formed driver-follower duplex as one unit of a correctly formed D-F duplex, whereas each origami, individually or in larger clusters, that was not part of a duplex was considered as one unit of an incomplete structure. We determined the percentage of correct structures on each micrograph as the ratio of correctly formed duplexes to the total structures on the surface (D-F yield in % = 100× D-F duplex / [D-F duplex + incomplete structure]). We then determined the average and standard deviation of the percentages of D-F duplexes over all 199 micrographs evaluated (**Ext. Data Fig. S8a-c**, **Suppl. Dataset S5**).

Gel electrophoresis. Assembly and purification of the transcribable linker as well as the transcription product of each transcription experiments were analysed on PAGE (6% PAGE gel in 1× TAE buffer 100 V 40 min). Origami assembly and purification was ana-lysed on with agarose gel electrophoresis (1% agarose gel 0.5× TAE buffer, 75 min at 80 V). All were stained with ethidium bromide and imaged with trans UV irradiation.

Statistics and Reproducibility. No statistical method was used to predetermine sample size, no data were excluded from the analyses and experiments were not randomized unless stated otherwise.

Measurements for transcription rate determination were taken from distinct experimental replicates.

For the determination of the angle distribution from TEM micrographs operator bias was excluded during the measurements, by blinding all the observed micrographs with a letter and number code, and only after the measurement of the angle the letter-number code was unblinded for the corresponding sample type. Every origami structure visible on TEM micrographs was measured only once. p values were obtained with two-tailed, heteroscedastic t-test. Error ranges were mean ±S.D.

For single molecule fluorescence data, three biological replicates were collected for each condition and analysed. To identify true single nanoengines, specific cut-off criteria were applied to each fluorescence time trace, requiring an average intensity of over

200 (a.u.; with noise at ~50 a.u.) for a duration of at least 10 seconds, with either the donor or acceptor (or both) showing single-step photobleaching. Only fluorescence trajectories meeting these criteria underwent individual background correction and further analysis. For Gaussian fits (**Ext. Data Fig. S5**) the error ranges were mean ±S.D.

For the equilibrium oxDNA simulations of the nanoengine the autocorrelation of the distributions was checked to ensure that the angle distribution was decorrelated. For the non-equilibrium simulations, the sampling frequency was increased by 100× as we do not expect ergodicity and wanted to extract rate parameters from the angle trajectories. In the other presented experiments, the investigators were not blinded to allocation during experiments and outcome assessment.

Supplementary Figures S1-S6

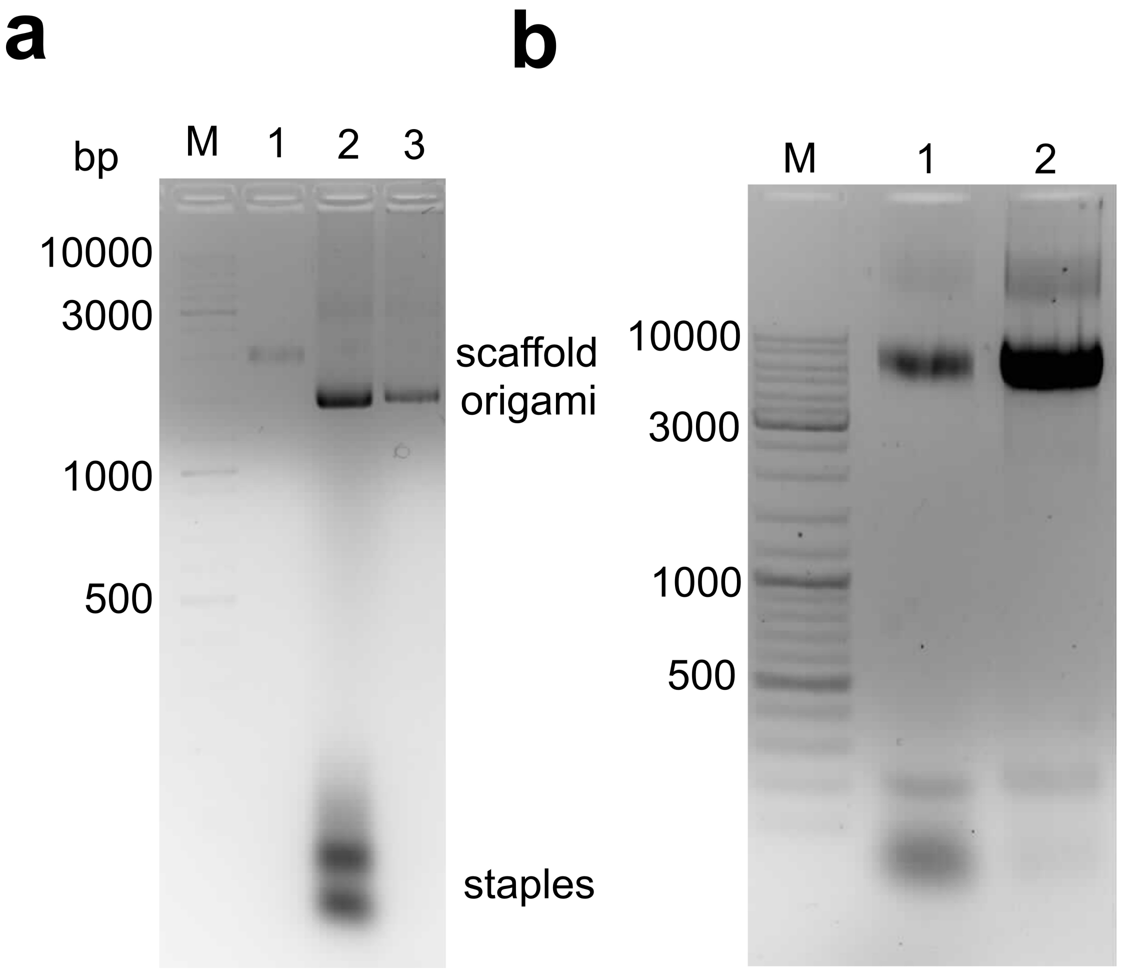

Suppl. Fig. S1. Gel electrophoresis of purified origami (**a**) 1% Agarose gel of filter-purified origami. Lane 1: M13MP18 scaffold, lane 2: assembled origami unpurified, lane 3: 100 kDa filter-purified origami. (**b**) 1% Agarose gel of PEG-purified origami. Lane 1: Assembled unpurified origami, lane 2: PEG-purified origami. The gels shown are uncropped.

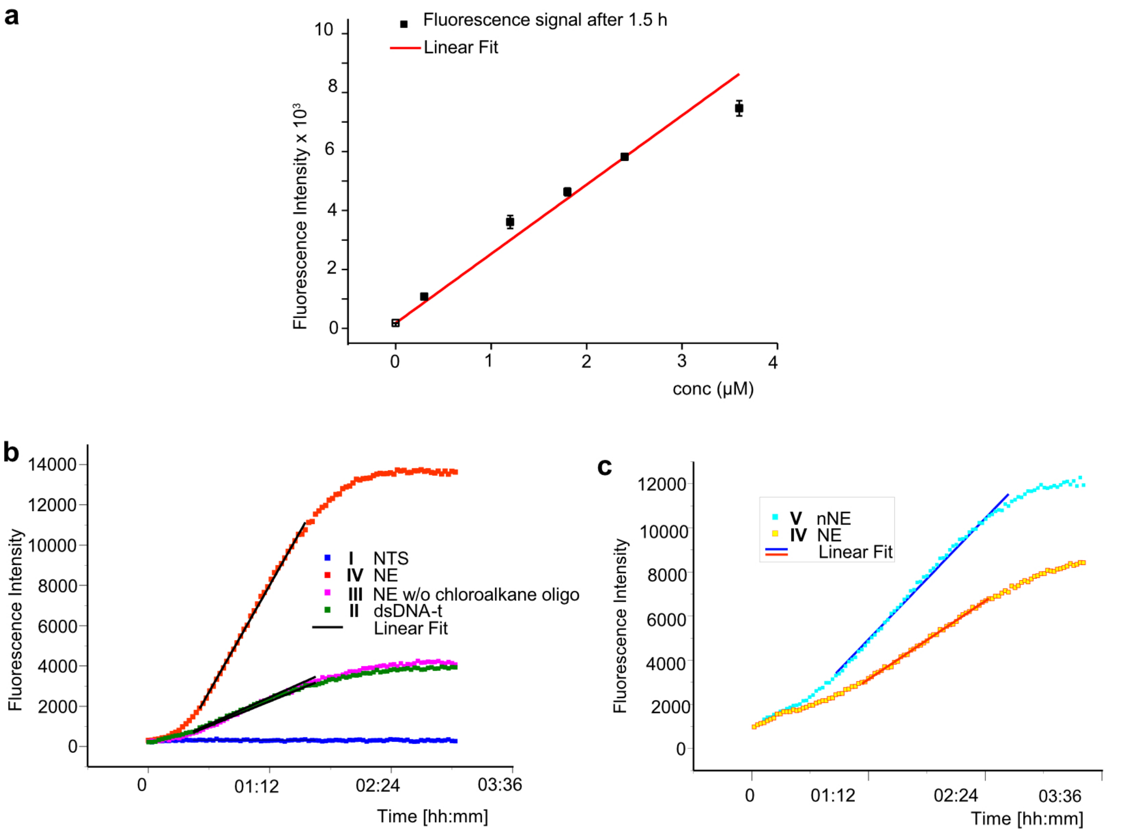

Suppl. Fig. S2. Bulk transcription characterization of the nanoengine by MB fluorescence (**a**) Calibration curve of the fluorescence intensity (F.I.) of the molecular beacon (MB) as a function of the concentration of the added complementary oligonucleotide. The measuring time was 1.5 h; after that time the F.I. stabilized without significant further photobleaching. (**b**) Exemplary transcription curves of constructs **I** (blue), **II** (green), **III** (magenta), and **IV** (red) shown in **Fig. 2a** with linear fit of the linear parts of the curves, (**c**) exemplary transcription curves of constructs **IV** (yellow) and **V** (cyan) shown in **Fig. 2b** with linear fit of the linear parts of the curves.

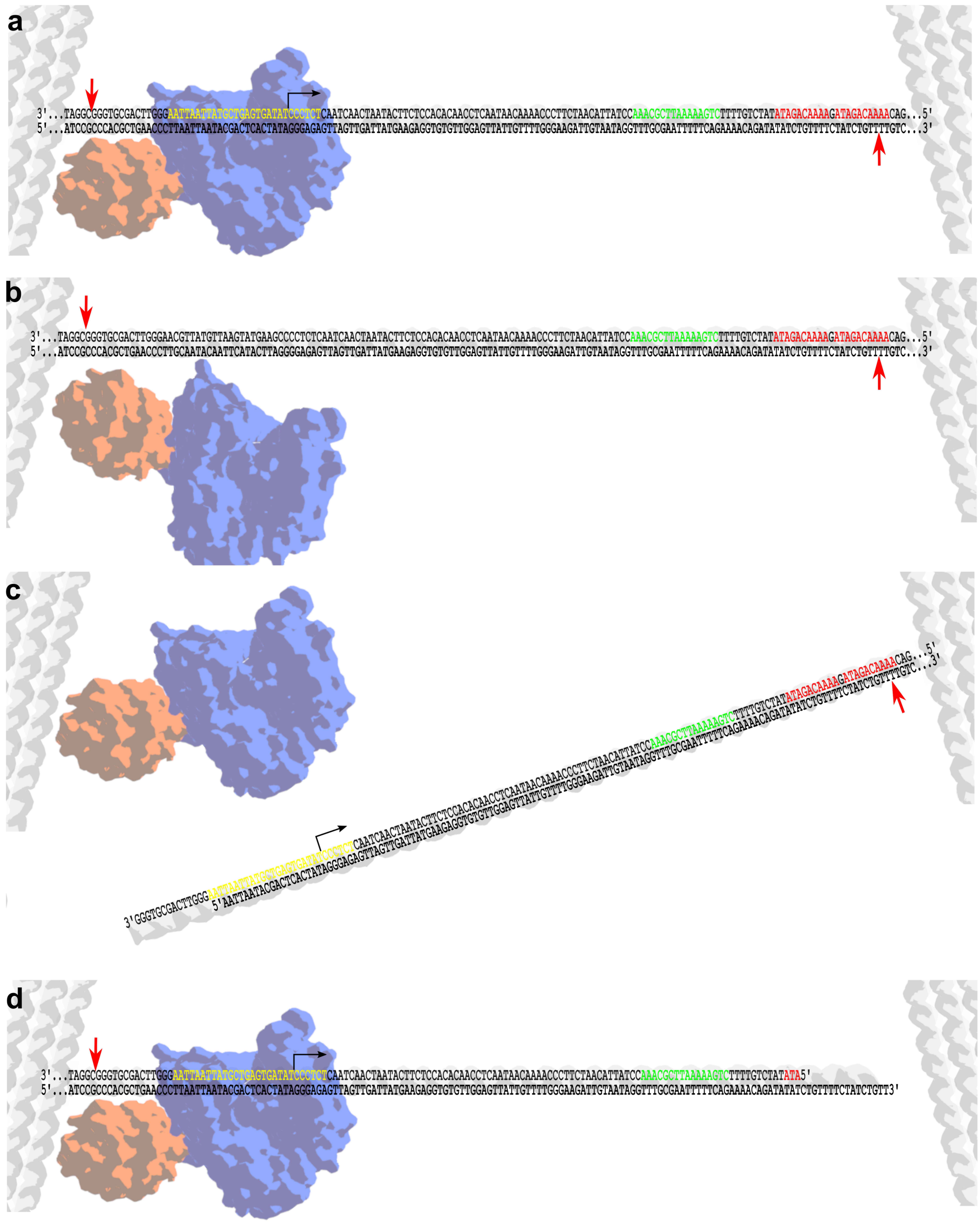

Suppl. Fig. S3. Representation of the various dsDNA-t. (**a**) nicked-nanoengine/dsDNA-t attachment site. Red arrows: position of the two nicks in dsDNA-t. (**b**) Red arrows: the same without the promoter region. (**c**) dsDNA-t, attached only on the opposite side of the HT-T7RNAP; red arrow: nick-position. (**d**) dsDNA-t, attached only next to the HT-T7RNAP; red arrow: nick position. Importantly, to avoid that the single stranded nicks weaken the stability of the dsDNA-t and cause its complete or partial detachment from the origami by forces generated during the pulling by the polymerase and/or during the formation of the transcription bubble, the template strand has no single nicks upstream of the promoter region while the nick is placed in the coding strand. Downstream of the promoter region the single stranded nick has been placed into the template strand while the coding strand is fully attached into the origami. This design prevents the two ss-nicks in the coding strand from leading to loss of the DNA-DNA duplex by forming a DNA-RNA heteroduplex as the amount of RNA increases over time. Both the leading and coding strands are anchored to the origami; their hybridization is still possible even when RNA transcription increases (HaloTag PDB: 5UXZ, T7RNAP PDB: 1CEZ)[16,17].

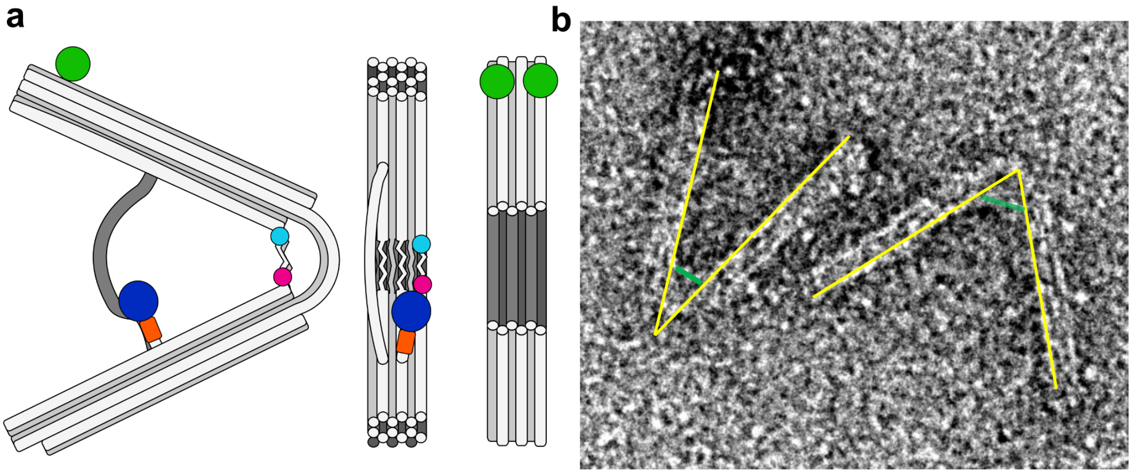

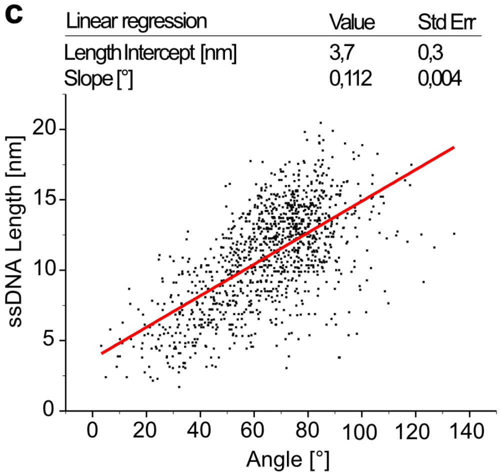

d

	Angle	Estimated distance
Median of angle in absence of transcription	67 °	7.1 nm
First quartile in presence of transcription	41 °	3.6 nm

Suppl. Fig. S4. Graphical representation of the nicked-nanoengine with the introduced FRET pair for intra origami FRET distance estimation. (**a**) The Cyanine dye pair, Cy3 (cyan) and Cy5 (magenta), was introduced in the hinge region adjacent to the single-stranded scaffold regions that give the origami its bent shape. The FRET pair was placed at the edge of the origami structure, and the two dyes were directed towards each other by extending the two modified staples and hybridizing over 7 nt on the nearest single-stranded sequence in the hinge. (**b**) To estimate the distance of the fluorophores in this area we measured the angle of several origami structures (NE and nNE with and without fluorophores, n=1146) by measuring the length of the single-stranded area (green line) together with the corresponding angle using imageJ. (**c**) The angles and distances were measured manually and therefore have high variability, but over a large set of data it was possible to fit the data with a linear correlation fit. Linear regression was used to estimate the spacing of FRET pairs in the hinge as a function of angle. (**d**) From the linear fit (Y= 3.7+0.112x) an estimate of FRET pair spacing in the open or closed conformation could be extrapolated. As a representative angle for the open conformation, the median of the distribution for the non-transcription nanoengine samples of 67° was chosen, resulting in an estimated dye spacing of ~7.1 nm. For the closed position angle, the value corresponding to the first quartile of the distribution of transcription active sample of 41° was used, resulting in an estimated dye spacing of 3.6 nm.

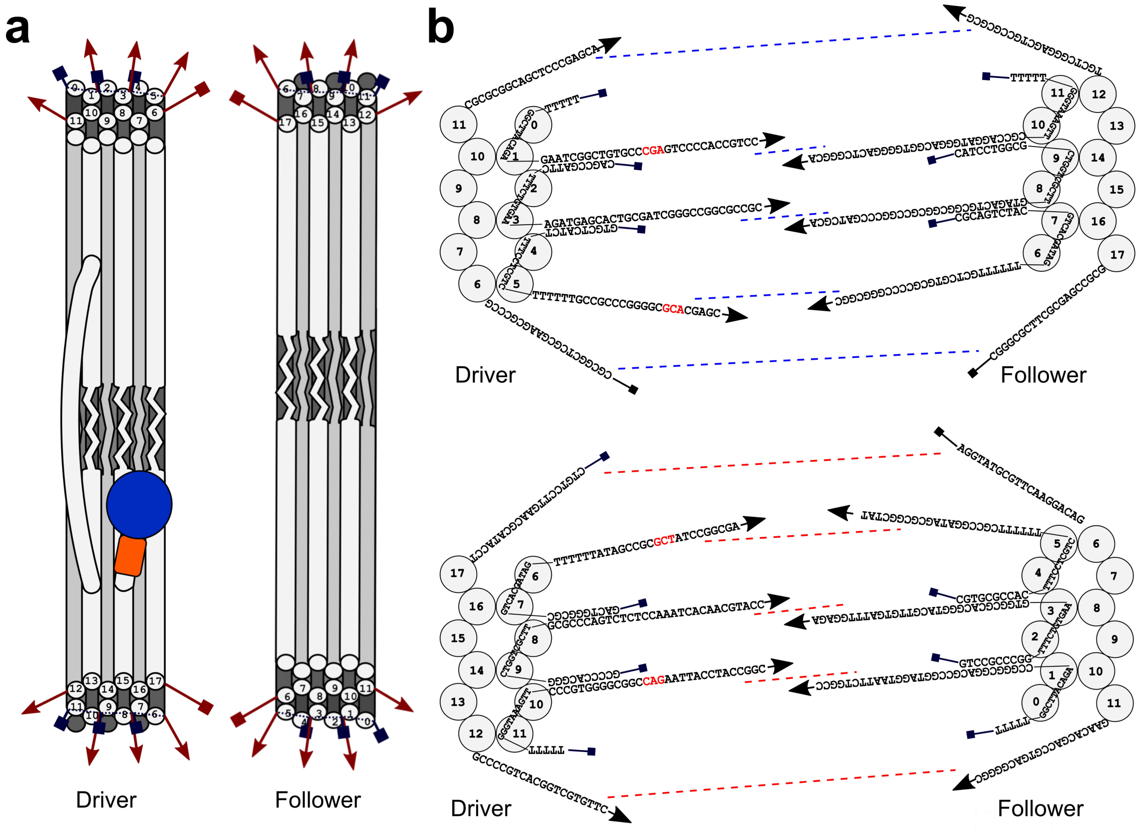

Suppl. Fig. S5. Graphical representation of the Driver Follower connecting sequences and structural characterization. (**a**) Schematic front view of the Driver and Follower units. Helices are numbered from 0 to 17 as a guide to better follow the positioning of the single-strand overhangs. The numbering of the helices is equivalent in both origami structures, please note how it is necessary to flip one of the two units upside down to be able to join the structures. (**b**) Detailed DNA sequences of the overhangs for the Driver (left) and for the Follower (right). Bases labelled in red in the Driver overhang sequences indicate LNA.

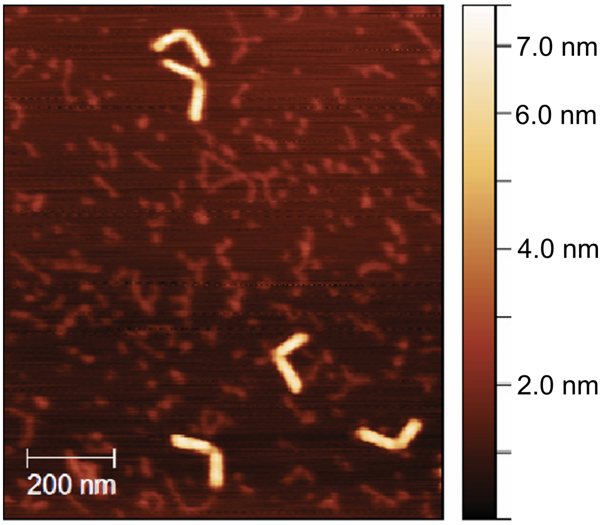

Suppl. Fig. S6. AFM image of the nicked-nanoengines with single Cy3, Cy5 and biotin staple strands (no HT-T7RNAP)

Supplementary Table S1

nicked-nanoengine no transcription			nicked-nanoengine transcription		
Angle (°)	particles	[%]	Angle (°)	particles	[%]
63	80	3.2	17	5	0.3
65	77	3.1	24	11	0.6
66	175	7.0	48	51	2.7
67	53	2.1	49	63	3.3
72	299	12.0	55	27	1.4
75	119	4.8	65	62	3.3
78	79	3.2	70	23	1.2
82	179	7.2	71	78	4.1
90	55	2.2	74	206	10.8
99	28	1.1	75	143	7.5
			77	180	9.5
undefined	1358	54.3	undefined	1054	55.4

Table S1 Distribution of particles from 2D class averages from negatively stained TEM micrographs grouped by aperture angles for nicked-nanoengine without transcription (nicked-nanoengine-no-transcription) and for nicked-nanoengine with transcription (nicked-nanoengine-transcription). Exemplary 2D classes and exemplary angle measurements without transcription and with transcription.

Supplementary Text

Supplementary Text 1. The following structural features of the DNA origami are confirmed by the 2D averaging: First, the two arms on the 2D average have a length of ~60 nm and a width of ~10 nm, consistent with the origami design. Second, three brighter strands interspaced by darker strands run along both of the origami arms. This pattern is probably caused by different coverage with staining agent on the specific honeycomb lattice structure of the origami. The central brighter strand connects both arms through the bent compliant hinge region. The brighter area that is on the inside of both arms of the V shape is connected by a bright but fuzzier region that corresponds to the ssDNA area in the hinge region. Third, on the outside of one of the arms three evenly spaced bright dots are visible. These dots are 21-22 nm apart and correlate with the designed position of the SA on the origami. Fourth, the opposing arm is nicked on the outer most extremity as expected from the design. Fifth, between the arms, on the inside the V-shaped structures, a faint cloud is visible that spans from arm to arm ~30 nm from each end. It is also noticeable how the darker shading that surround the origami is interrupted by thin, lighter shading where the dsDNA-t contacts the origami body. This cloudy density is consistent with the position of the highly flexible dsDNA-t and HT-T7RNAP.

Supplementary Text 2. dsDNA-t attachment only to the origami arm located opposite the HT-T7RNAP anchored arm produced virtually no transcripts (**Fig. 2d**, column 1). A plausible explanation is that the free end of the dsDNA-t has a higher degree of freedom, making it more difficult for HT-T7RNAP to find the promoter region (**Ext. Data Fig. S3b**). Conversely, attaching the dsDNA-t only to the site next to the HT-T7RNAP in the corresponding origami arm (constructs IX and X) resulted in a relative transcription rate of 1.4 (**Fig. 2d**, column 2), which is higher than that observed for the nanoengine (**Fig. 2d**, column 3), but considerably lower than that of the nicked-nanoengine (**Fig. 2d**, column 4). The lower rate of construct X relative to the nicked-nanoengine is consistent with the notion that the higher degrees of freedom of dsDNA-t make it less available for the immobilized protein also as indicated by the molecular dynamics simulations (**Suppl. Text 6**). Furthermore, we hypothesize that the active opening of the origami in the nicked-nanoengine results in a straightened dsDNA-t, which places the promoter region in an appropriate position for T7RNAP to start a new transcription cycle. The higher rate of X compared to the nanoengine can be explained by the lack of torsional stress that accumulates in the dsDNA-t during transcription.

Supplementary Text 3. Note on 2D-class averaging. Importantly, the flexible and highly dynamic nature of the nicked-nanoengine inevitably leads to the 2D classification algorithm averaging the most frequently occurring angles, resulting in an overrepresentation of these angles and an underrepresentation of the less populated and more dynamic states. Reference-free 2D classification in RELION is best suited to produce clear images of rigid features, while leading to blurring of flexible features or their underrepresentation over the more represented rigid elements.

Supplementary Text 4. Because the timescales of physical processes in coarse-grained simulations scale differently and the implicit solvent used in the simulations neglects fluid dynamic effects, we report the closure and opening rates relative to nicked-nanoengine rather than absolute numbers to capture the relative effect of structure modification on dynamics.

Supplementary Text 5. In contrast to the smFRET data, the closure rates observed in the oxDNA simulations were 2-3 times faster than the opening rates. This need not be a contradiction, as smFRET measures the actual movement of the structure, where T7RNAP moves step by step from one nucleotide to the next, with a dwell time that depends on NTP diffusion to the active site. MD, on the other hand, represents an ideal case where the polymerase pulls on the DNA with a continuous force of 16 pN.

The effect of temperature on the pull/release simulations was similar to that of the equilibrium simulations. The rates of opening and closing were slightly increased and the variance of replicates of closing and opening simulations increased at 37 °C, as would be expected due to the increase in the accessible phase space (**Ext. Data Fig. S7a,b**).

Supplementary Text 6. To a first order approximation, transcription can only be initiated when the distance be-tween the polymerase attachment site and the promoter sequence is within 8.6 nm (ignor-ing the specific orientation required to initiate transcription). For nicked-nanoengine the promoter region spends 70.06±0.01% of the time within the 8.6 nm distance, but only 61.1±0.01% when dsDNA-t is anchored only next to the polymerase. This reduction suggests that the higher number of degrees of freedom in the latter case reduces the probability that the promoter region is in a favourable position for transcription initiation (**Ext. Data Fig. S7c**). The polymerase is much smaller than the arm-to-arm distance in the nanoengine. In the average open form of the nicked-nanoengine, the end-to-end length of the dsDNA-t is 37.5 nm (**Ext. Data Fig. S7d**), indicating that there is sufficient space between the two arms of the nicked-nanoengine in the open state to properly accommodate the polymerase.

Supplementary Text 7. In case of D-F we hypothesize that the addition of a complete hinge to the dimeric complex adds further resistance to the closing, which is compensated just enough by the reopening spring effect to result in no significant change in the overall transcription speed. In contrast, in the case of the F-ss-hinge and the F-soft-hinge the closing speed is not significantly affected compared to the D alone due to less resistant springs, while the reopening is slightly enhanced by the higher entropic degree of the hinge areas resulting in a slightly increased transcription rate.

Supplementary Text 8. The nanoengine provides an actively moving nanoengine that operates fully autonomously; once the NTP fuel is added to the system, the structure sets itself in motion, and continues pulsation without further input until the fuel is consumed. The use of NTPs as fuel is likely to be advantageous if in future applications the nanoengine is to be operated in a biologically compatible manner in living cells, where NTPs are abundantly available. For application in simple in vitro systems, we see the advantage that NTPs are readily available, cheap, and, like all other components of the nanomachine, water soluble. Finally, changing the NTP concentration in vitro can be exploited purposefully to reversibly stop and start the nanoengine, as we show in this study, or to modulate its pulsing frequency. Such control may enable, for example, future applications of the nanomotor to drive chemokinetic nano-swimmers that automatically change their swimming behaviour as a function of NTP concentration.

Supplementary Text 9. Achieving autonomous active motion in a bio-hybrid DNA-nanostructure constitutes an advance, significantly enhanced here by the demonstration that nicked-nanoengine can transfer its movement and force to another device. We view the applicability of the nanomachine as essentially analogous to its macroscopic counterpart, the internal combustion engine or electric motor, where it can be used as part of a more complex system that converts the simple motion generated by the engine into more complex functional outputs. In this context, a particular challenge is likely to be increasing the mechanical performance of the machine, especially towards the precise synchronization of multiple nanomotors.

These advances, and the prospect of expanding architectural complexity by attaching single-stranded overhangs for coupling with other origami, or by incorporating chemical or biochemical modifications, place few limits on the scope, nature, or range of the motions - linear, rotating, pulsating, or contractile. In principle, our nanomachine can now be introduced as an active core component, or "beating heart", into larger passive nanomachines whose functions manifest themselves through designed downstream sequences of motion.

Supplementary References

1 Poppleton, E., Mallya, A., Dey, S., Joseph, J. & Sulc, P. Nanobase.org: a repository for DNA and RNA nanostructures. *Nucleic Acids Res* **50**, D246-D252 (2022). https://doi.org:10.1093/nar/gkab1000

2 Zhou, L., Marras, A. E., Su, H. J. & Castro, C. E. DNA origami compliant nanostructures with tunable mechanical properties. *ACS Nano* **8**, 27-34 (2014). https://doi.org:10.1021/nn405408g

3 Shi, Z., Castro, C. E. & Arya, G. Conformational Dynamics of Mechanically Compliant DNA Nanostructures from Coarse-Grained Molecular Dynamics

Simulations. *ACS Nano* **11**, 4617-4630 (2017). https://doi.org:10.1021/acsnano.7b00242

4 Los, G. V. *et al.* HaloTag: a novel protein labeling technology for cell imaging and protein analysis. *ACS Chem Biol* **3**, 373-382 (2008). https://doi.org:10.1021/cb800025k

5 Valero, J. & Famulok, M. Regeneration of Burnt Bridges on a DNA Catenane Walker. *Angew Chem Int Ed Engl* **59**, 16366-16370 (2020). https://doi.org:10.1002/anie.202004447

6 Valero, J., Pal, N., Dhakal, S., Walter, N. G. & Famulok, M. A bio-hybrid DNA rotor-stator nanoengine that moves along predefined tracks. *Nat Nanotechnol* **13**, 496-503 (2018). https://doi.org:10.1038/s41565-018-0109-z

7 Yu, Z. *et al.* A Self-Regulating DNA Rotaxane Linear Actuator Driven by Chemical Energy. *J. Am. Chem. Soc.* **143**, 13292-13298 (2021). https://doi.org:10.1021/jacs.1c06226

8 Ouldridge, T. E., Louis, A. A. & Doye, J. P. K. Structural, mechanical, and thermodynamic properties of a coarse-grained DNA model. *J Chem Phys* **134** (2011). https://doi.org:10.1063/1.3552946

9 Rovigatti, L., Sulc, P., Reguly, I. Z. & Romano, F. A comparison between parallelization approaches in molecular dynamics simulations on GPUs. *J Comput Chem* **36**, 1-8 (2015). https://doi.org:10.1002/jcc.23763

10 Snodin, B. E. *et al.* Introducing improved structural properties and salt dependence into a coarse-grained model of DNA. *J Chem Phys* **142**, 234901 (2015). https://doi.org:10.1063/1.4921957

11 Sulc, P. *et al.* Sequence-dependent thermodynamics of a coarse-grained DNA model. *J Chem Phys* **137** (2012). https://doi.org:10.1063/1.4754132

12 Thomen, P. *et al.* T7 RNA polymerase studied by force measurements varying cofactor concentration. *Biophys J* **95**, 2423-2433 (2008). https://doi.org:10.1529/biophysj.107.125096

13 Durniak, K. J., Bailey, S. & Steitz, T. A. The structure of a transcribing T7 RNA polymerase in transition from initiation to elongation. *Science* **322**, 553 (2008). https://doi.org:10.1126/science.1163433

14 Martin, T. G. & Dietz, H. Magnesium-free self-assembly of multi-layer DNA objects. *Nat Commun* **3**, 1103 (2012). https://doi.org:10.1038/ncomms2095

15 Valero, J. *et al.* Design, assembly, characterization, and operation of double-stranded interlocked DNA nanostructures. *Nat Protoc* **14**, 2818-2855 (2019). https://doi.org:10.1038/s41596-019-0198-7

16 Liu, Y. *et al.* The Cation-pi Interaction Enables a Halo-Tag Fluorogenic Probe for Fast No-Wash Live Cell Imaging and Gel-Free Protein Quantification. *Biochemistry* **56**, 1585-1595 (2017). https://doi.org:10.1021/acs.biochem.7b00056

17 Cheetham, G. M., Jeruzalmi, D. & Steitz, T. A. Structural basis for initiation of transcription from an RNA polymerase-promoter complex. *Nature* **399**, 80-83 (1999). https://doi.org:10.1038/19999
