# Supplementary material for: A rhythmically pulsing leaf-spring DNA-origami nanoengine that drives a passive follower
Source: Nat Nanotechnol. 2023 Oct 19;19(2):226–36. doi: 10.1038/s41565-023-01516-x (PMC10873200; doi:10.1038/s41565-023-01516-x)
Supplement: Supplementary file 4 — Detailed protocol and procedure for the assembly and purification of the DNA nanoengine origamis and complete list of all ODNs used for the DNA nanoengine origamis. [file 41565_2023_1516_MOESM4_ESM.pdf]

Detailed protocol and procedure for the assembly and purification of the DNA nanoengine origamis.

Complete list of all ODNs used for the DNA nanoengine origamis

All ODNs are listed from 5' → 3'

Complete list of all ODNs used for the DNA nanoengine origamis	
Name	Sequence
Hi-MC-1	AAATTCAAAAATAATTCGCAACCCGTGAGGGGTTGC
Hi-MC-2	GAAAGGCTATCAGGTCATTGCCTGGCAAACACGGTTGAAGCCCCATGT
Hi-MC-3	TTCAACCGCCTGGCCCTGAGAGAGTCCAGGGTTGCATCTTCAG
Hi-MC-4	ATTAGCCAGCTATAAAAAATATCTACATTTAACAATTTCTG
Hi-MC-4-dsDNA	ATTAGCCAGCTATAAAAAATATCTACATTTAGACAAAACAGATAGAAAACAG
Hi-MC-5	TTTATAAAACAGAGGTGTTGAAAGATAAGTTTCCGGTAAAAG
Hi-MC-6	GATATGCGCGAACTGATAGCCGTCCAAACGTCTTAAATCGCA
Hi-MC-7	GGGTCAAATCACCATCAATATGATACCCTGTTAGTAGCAGTTTCATTTT
Hi-MC-8	TGGCAACTAATGCAGATACATATATTATGCGATTTTAAAGAT
Hi-MC-9	AGCGTAAAACGACGGCCATTACGCGCTGCGCATCGGCCGCC
Hi-MC-10	GAATTGAATGGCTATTATTGAGGACCTTATTATTTTGCTGTT
Hi-MC-11	TAAAAAGGAGCGGGCGCTAGGGCGTAGCGGTCCCGGAAACCG
Hi-MC-12	TACTGCAACAGGAAAAAATATAATGAGGGTATAACTGACTAA
Hi-MC-13	CTGATTACGAGGCATAGCCACATTCTCATTATACCAGTACGA
Hi-MC-14	AGCCCCCGATTTAGAGCTTGACGGACTATGGTTAGCGGTCAC
Hi-MC-15	CATATTTACATTGGCAGAAACCACAGAGCAAGAAAATACAAC
Hi-MC-16	CGGACTACAACGCCTGTCCCTCAGCTCAGTAGCTA
Hi-MC-17	CCTAACACTATCATAACATCAGTTTTGGGAAGAAAAATGCCC
Hi-MC-18	AAACCGAGAAAAACCGGATATTCATTGCTCCAACCCAGCCGC
Hi-MC-18-bio(-3')	biotin GCTCA TATTCATTGCTCCAACCCAGCCGC
Hi-MC-18-bic(-5')	AAACCGAGAAAAACCGGA TGAG
Hi-MC-19	TAACGCCATATTTAACACAAAAATGAAACAAACCGGAATAA
Hi-MC-20	TAACCTTCCTGTAGCCAACATTAAGCCGCCGATAT
Hi-MC-21	GAAGAAGATTAATCATATGTACCCAGAGAATCGAAAGGCGA
Hi-MC-22	AAGGCTCCAACCATCGCCCACGCACGGGATCATATAGATCA
Hi-MC-23	GCAGGTGAGAAAGGCCGGAGACAGAGAAGCCTGGCATCTGATTCCCTCC
Hi-MC-24	TCGAATCGGAACCCTAAAGGGAGCACGTATATCCTCAAGAAT
Hi-MC-25	GTCGATCCCCGGGTACCGGAAGGGTGTTTGGAGGGCCATCGC
Hi-MC-26	ATACAGAGCCCACAAGACATATTCTAAAGAAATTGCGTCGGA
Hi-MC-27	TTTGCGTAACGATCTAACCGTACTAGTATAGCACG
Hi-MC-28	ACTAAACAAAGTACAACGGAGATTCGCGACCTACCCAAAAGGCTTCTAC
Hi-MC-29	AGGACTCTTTATACAAAGGAGAATATTGAGCCTCAGAGTGAC
Hi-MC-30	GGCGTGCAGCAAGCGGTCCACGCTGAGGCGGTACACCACCCAG
Hi-MC-31	CGTGGAAAGCCGGCGAACGTGGCGATGCGCCCCAGGCGATTA
Hi-MC-32	AGGGAAAGAGGCAAAAGAATACACACTGACCGACCAGGCAACTTTAATT
Hi-MC-33	CACAGACACTTTACAGAACTGTTATCCTCGAAATCTGTATCATCGCCTG
Hi-MC-34	TCAAATAATTACTAGAAAACAGAAACTGATTATTTGACGTCAA
Hi-MC-35	ATCTGAGGAAGTTTCCATTAAACGTCAGCAGACAACAAAAGGAGCGCTA
Hi-MC-35-bio	biotin GCACA GTTTCCATTAAACGTCAGCAGACAACAAAAGGAGCGCTA
Hi-MC-36	GAGTCAAATATCGCGTTTATTCATAGAATGGGCAA
Hi-MC-37	GCGAGCGGATTGCATCAACGAGAATTGGCCTGAAG
Hi-MC-38	GAATATAAGAATAAACACAGATTTTAGCGGAAATGGATTCACT
Hi-MC-39	GCGAAACATCGCCATTACACTAACTAAAGGTACTTGCGGAAC
Hi-MC-40	GCTCGAACGAACCACCATATCTAAGAAACGCGGCGTTTAAAG
Hi-MC-41	CAGGATCGCACATTCGCCTCACATGGAAGCATAAAGTGTCAT
Hi-MC-42	TCAATAAATTTTAAAAATAAGAGAGGCATAATGGTTTGAAATCCTT
Hi-MC-43	TTTTAAGAGGAAGCCCGATGCTTTAAATAAAATAA
Hi-MC-44	GCGTCTTTCCAGACGACGACAATAAACAACATGTT
Hi-MC-45	CTCATTCCAGTAGCGACAGAACAGTGCCTTGAGTA
Hi-MC-46	TAGTTAATTTCGCATAGGCTGGCTCATAAGGGGAGGTTATA
Hi-MC-47	ATCGTCAAAATCATAGGTCATTTGAGGAAGGTGCAGAAGGACG

Hi-MC-48	GTGGACGGGCTTTCCAGTCGGGAAGTTG
Hi-MC-49	GTACGGAAACATGTTAGAATAGATAAAGAAGATGATGAATAA
Hi-MC-50	AGCCTGAATCCCCCTCAAAAAGACTAGTACCTTTAATTGCAAT
Hi-MC-51	AGAATGTGAGCGAGTAACGTCTGGACGTTAATATTTTGCAA
Hi-MC-52	TGTAAAGCGCCTCCAGCGCGCATCACGGCGGATTGACCGTAATGGGTTG
Hi-MC-53	CTGCAGTATAAAGCCAAAACAGGGGATAACCGCCACCCCAGA
HI-MC-53-dsDNA-cp	5'-P-CGGATACGTAAAAAGCCTGCAGTATAAAGCCAAAACAGGGGATAACCGCCACCCCAGA
Hi-MC-54	ATATTTAGGTTGGGTTATAACAAACAGATTAGAGCCCTAGGAG
Hi-MC-55	CGATTGAAAATATATTCGGTCGCTGGGAGTTTTCTAAGATA
Hi-MC-56	CCGTTCCAGTAAGCCTGGATAGCGTCCA
Hi-MC-57	ATTTTTCGAGTAAACAGCTTGATAGGGTAGCCATCGTAAGA
Hi-MC-58	CAAGTAGCTTAGATTAAGTATATGTAATATCTCCTGCAAGCGC
Hi-MC-59	GCCTCCAATCGCAAGACACGAATTAGTATTAGAATATTTCGGT
Hi-MC-60	AATGACCATTAGCTATATTTTCATAAATTTTTTAACGTACG
Hi-MC-61	AAGTCCTCAGAACCGCCAAGCATTCAACTTTCAACAGTTAAAA
Hi-MC-62	TTAAGTAACATAATAAAAATTAACTTTGGAACAAGAGT
Hi-MC-62 ExtCy5	TTAAGTAACATAATAAAAATTAACTTTGGAACAAGAGTCCACTAT-Cy5
Hi-MC-63	AGCGCGAACGGCGAGCTGAAAAGGTTTATTTGCAGCCTTGA
Hi-MC-64	TAGGCGAATAATGAACGGTGTACAAACTTTGTAGCGAACGA
Hi-MC-64-bio(-3')	biotin GCGTT GTGTACAAACTTTGTAGCGAACGA
Hi-MC-64-bic(-5')	TAGGCGAATAATGAACG AACG
Hi-MC-65	TTCCGAAGAGTCAATAGTTGGAAACTCAACAGAGGCGGTGGCG
Hi-MC-66	ACGCTTGAGCCCACGGAGAATTGAATTACCTTTTTTAAGAAT
Hi-MC-67	ATCCAGTAGGGCTTAATTTACAGATAAGCCCGGAACCGCAC
Hi-MC-68	AACCACAGACAGCCCTCCCGCCACGCCGTCGACCC
Hi-MC-69	ACAAGAATCCTTGAAAACGCTTCTGTCAAATAGAGCCAGCGCG
Hi-MC-70	GTACGGCCAACGCGCGGTTATACTGGGAAGGCGGGCCTATAA
Hi-MC-71	CGGTCGCCACCGCTAATAATGGCAATATTTGCACGTAAAAAGC
HI-MC-71-dsDNA-p	CGGTCGCCACCGCTAATAATGGCAATATTTGCATCCGCCCACGCTGAACCCTT
Hi-MC-72	ACCCATGTAATTTAGGCAAACGATCTATCTTCATCTTTAGTC
Hi-MC-73	AGGCACCCTCATTTTCAGTAACACCTAAAGGAATTGTGAATC
Hi-MC-74	AACAACAAAAAACCAAAATAGCGAGAGGCTTTTGCAGTTTTA
Hi-MC-75	ATGATTCAACCGTTCTAGCTGATAGGTTGTAAATCATAAACTAAATAAT
Hi-MC-76	CTATCGAGGTGCCGTAAAGCACTATTAGAATAAAGTATCCAG
Hi-MC-77	TGCCAGTACAAAAGGTAAAGTAATTCTGTCCAGAGAACCGAGAGCG
Hi-MC-78	CGATAAAACACTCATCTTTGACCCGGTCAATGACCTTCGGGCTTGGAAC
Hi-MC-79	TACAGTCACACGACCAGTTATCATGCAATAGTTTTTGTTAGA
Hi-MC-80	TTTTCGAGCCAGTAATTTATCCCAATCC
Hi-MC-81	CGAAGCTTGCATGCCTGGACGGTGGCGATACGCCGCCACACC
Hi-MC-82	TGGGGAAATACCTACATTCAGATGTCAGAGAAAGCGCACATT
Hi-MC-82-Halo	Halo (ligand O2) AAGCGATCTAGCCCTACATTCAGATGTCAGAGAAAGCGCACATT
Hi-MC-83	CCCGGTAAAATACGTAATGCCACTGCTTTTGTAACCGATCTCCAATCAG
Hi-MC-84	TTGAACCGCCACCCTCAAGTACAAAGTGAGAATAGAAATTTC
Hi-MC-85	TTAAGAAAGGAAGGGAAGAAAGCGCCACCACAGAGGGTAAAT
Hi-MC-86	AAACAAACAGTTCAGAAAAAAAGATTGATAAGAGGTCATTTCC
Hi-MC-87	GAATCACTGCATTCCACACAACATAATTGTTGGTTTTCCCAGTCAGGGA
Hi-MC-88	GACCCAGCGATTATACCAAGCGCGTAGCCGGTGACAAGCACCAGACAGG
Hi-MC-89	GCTGATTGCCCTTCCCAGTGATAGATGGCAGCTTTCCGGCACGCCG
Hi-MC-89-con-drive	CTGTCCTTGAACGCATACCTCAGCTGATTGCCCTTCCCAGTGATAGATGGCAGCTTTCCGGCACGCCG
Hi-MC-89-con-replica	CGGGCGCTTCGCGAGCCGCGCAGCTGATTGCCCTTCCCAGTGATAGATGGCAGCTTTCCGGCACGCCG
Hi-MC-90	AAGGTGAATTTCACAATTGGCAAAAGTACATAAATCAAACGC
Hi-MC-91	AGTTTCGGATTCTCCGTGAACGCCATCGCATTAAATTTTAAAA
Hi-MC-92	AACCTCAATCGTCTGAATTATCATATTGAGTGAGAATATTGC
Hi-MC-93	TCGTTGATGAGGTAATAGTAAAATGTTTAGAGTCATAC
Hi-MC-94	ACCGTGTAGGTAAAGATTCAAAAGAGGATAATTGGGGCAGTAGATATTA
Hi-MC-95	GCAGAGGAAGAACGCAGCTAATGCAGAATACAATTGCATGATAGCA
Hi-MC-96	AATGTTTATCAACAATAAGTTGCTACGCAGTGTCACCACCTG
Hi-MC-97	CACAATATGCCAGGCAAGGCAAAGCATAAAGACACCCTCAG
Hi-MC-98	ATCAATTAATGCCGGAGAGGGTAGCTCAGAGAATTAGCGTCAACATAGC
Hi-MC-99	TTTCAAAATCTGAAATATTTGCGGATACAGTAACAGTAACCG

Hi-MC-100	GCCACGAAGGCACCAACCTAAAACACTTGCAGAGGCAGATAATTTGGAA
Hi-MC-101	GGGCCCAAATCAAGTTTTTTGGGGAACAGGAAACCTATGCAA
Hi-MC-102	GCCCAGTGCCACGCTGATCAAACCTACCAGCTTACCGCCAGC
Hi-MC-102-biC35	GCCCAGTGCCACGCTGATCAAACCTACCAGCTTACCGCCAGCATCTGAGGAA TGTGC
Hi-MC-103	AGCCTATTTTTGAGAGATCTACAACGGTAATGCATGTCGTATAAGTTA
Hi-MC-104	TCTCACCACCAATAATACAGAAGGCAGGTTTAACGTCAGCGT
Hi-MC-105	ACCGGAACCCATGTACCGGGATAGCTGAGACACGT
Hi-MC-106	ATATCGACAGTAACTGTTTCTGCCTTGGGGTGAATAATGAGCT
Hi-MC-107	GGCGTAAATTATCAAGAGTAATCTAACGAGGCAAGATTGAT
Hi-MC-108	ACGGGCCCACTACGTGAACCATCAATTTTAGAATGCCCATG
Hi-MC-109	TTTGTGCCAGCTGCATTGAGCTAACATTCAGCAGCTGGCGAT
Hi-MC-110	AACGTCTGGAATTAACATCCAATACCAAAAATTAGACGTTC
Hi-MC-111	CTGCAGCAAATGAAAAACACCTTGGGCGACATTATTTTAACG
Hi-MC-112	AGAGGTAGAAACCAATCAAACGCGAAAAGACACATTTGGGAGA
HI-MC-112-dsDNA-c	5'-P-TTGTCACAATTTCTGAGAGGTAGAAACCAATCAAACGCGAAAAGACACATTTGGGAGA
Hi-MC-113	GCCAATTCGTAATCATGGTAAAGCCAATGAGTAATGAATTTG
Hi-MC-114	TCAGGTAATAAAAAGAAGTTTTGCCAGAGGGTACAGGA
Hi-MC-115	TTACCTCAGAAAAGTCACCTGATTTAGAACCTACCATACGAG
Hi-MC-116	TTAGCTGTCTTTCCTTAAGGCTTATATTTTGATCACCGGGTT
Hi-MC-117	ATGGCTTGCATTTTCGGTCATAGTAAGCAGATAGCTAAACAG
Hi-MC-118	AAGTTATGTAAATGCTGACTGAGCAAATACATGTCTTTATAAA
Hi-MC-119	TTACTTTAGTATCATATGTCAAAATTTCATCACGCTCATTAAT
Hi-MC-120	CGGCCCCATCCTAATTTACCTCCCGGGCAACAAAAATCATAAG
Hi-MC-121	TGAAAGAACGGGTATTAAGCAAGCAAATTCAATTATTCGAT
Hi-MC-122	GCACCGTATATACCAGACGACGATTTATTACAATAAAACGAACTA
Hi-MC-123	GACCGGATTTCGCACGTTTGCACCGAAGAAGTTTGCATCGGG
Hi-MC-124	CTACCTGAACAAGAAAATTGAAGCAGAAAATACCATTATATT
Hi-MC-125	GTGTACTGACTGTAGCGCGTTAGTTACCAGAAGGACCTAATT
Hi-MC-126	GGTCACCATTACATACAAACTAATATCAAGAAAACAAACTC
Hi-MC-127	AATGCCTGAGTAATCTCATATCCTGTTTAGATACAAGC
Hi-MC-128	CCAAGTGTGATAAATAAGGATGAATAACAAAGATTCACCTTCT
Hi-MC-129	TGCAATCAAAAGAATAGCCCGAGAATTAGTATCCTCATCCAG
Hi-MC-130	CGATGACCATAAATCAAGAAGCAAGATGGCTTAGAGCTGTA
Hi-MC-131	CAATGAGTTTCGTCACCGAGCCACATTAGGATTGC
Hi-MC-132	ACGGGGTTCAAGTTTGCCTTTGAAACGCAATAATACTAACGA
Hi-MC-133	AGGGTCTTTACCCATCAGCTTTCTTGAATATAATGCTGTGTT
Hi-MC-134	ATCTCCTGTTTGATGGTGGTTCCGATCGGCCGGCAGGTTCAG
Hi-MC-135	ATACAGGAGGTTTAGTAATAGTTACTGTATGGGATTTTCTTT
Hi-MC-135-biC147	ATACAGGAGGTTTAGTAATAGTTACTGTATG AGGAG
Hi-MC-136	CCCTTTTTAAGAAAAGCCCCCTTATTAG
Hi-MC-137	GGGCGATCCAGAATACAGTGCCCGTAATAGTGAATATCAACGTAACAAA
Hi-MC-137 Ext Cy3	Cy3-TCTATCAGGGCGATCCAGAATACAGTGCCCGTAATAGTGAATATCAACGTAACAAA
Hi-MC-138	ATTTGTATCGTTGCGCCGACAATGCGAAAGAGCCCAATAAC
Hi-MC-139	AAACGAGCTTCAAAGCGAATACTGCGAATTTATCACGCAAGGGACA
Hi-MC-140	CCTTAACAGTAATTCTACTAATAGAATACTTACATAAACGC
Hi-MC-141	AGGCAAGCCCAAACGCCAAAAGGAAAACATGCAGA
Hi-MC-142	CCATATTATAAGAGAATATAAAGTACCGTACAAAACGAACAATTCA
Hi-MC-143	GCTTGAGGACTAAAGACTTTTTCAGGAACGACCGATAGGTTTATCGAAT
Hi-MC-144	TTGTAGGGTTGAGTGTTGTTCCAGCGTTGTAAAAGCGCTCAT
Hi-MC-145	AGATTAAATTAAAATTAAGAACTACGTAACACTTCGCTAGT
Hi-MC-146	CGATACCGCACTCATCGGGAATCAGCCAAAGGGAAGGTTGAT
Hi-MC-147	AATGACGGAATATGGTTCTCAATCGAGTGAATAACCTTATAG
Hi-MC-147-bio	biotin CTCCT GGATTTTCTTTAATGACGGAATATGGTTCTCAATCGAGTGAATAACCTTATAG
Hi-MC-148	TGACATCGATTAAGACTTTTAGAATTCATTTCAATTACTGCA
Hi-MC-149	TGCGAGGGAGACAAAAGCTGAACCTAAATCGTCGCTATTCCCTTAGCAAGCC
Hi-MC-149-con-drive	TGCGAGGGAGACAAAAGCTGAACCTAAATCGTCGCTATTCCCTTAGCAAGCCGTCGCGCGGCAGCTCCC GAGCA
Hi-MC-149-con-replica	TGCGAGGGAGACAAAAGCTGAACCTAAATCGTCGCTATTCCCTTAGCAAGCCGTGAACACGACCGTGACG GGGC
Hi-MC-150	TGCGACTATTATAGTCAAAATCAGAGGTTGATTGC
Hi-MC-150-comp	TGCGACTATTATAGTCAAAATCAGAGGTTGATTGCTGGGGAAATA GCTAGATCGC

Hi-MC-151	CGCACCAGGCGCTGCAAGGCGATTACGCCAGATCCGCT
Hi-MC-151-con-drive	CGCACCAGGCGCTGCAAGGCGATTACGCCAGATCCGCTCAGCCCCGTCACGGTCGTGTTC
Hi-MC-151-con-replica	CGCACCAGGCGCTGCAAGGCGATTACGCCAGATCCGCTCATGCTCGGGAGCTGCCGCGCG
Hi-MC-152	TCTGGAATCGTCATAAATTAATTCTCCAACAGGTCAGGTTAG
Hi-MC-153	TGTATCAAGTTTTGAGACGTTAGTAAATAGCT
Hi-MC-153-con-drive	CGCGGCTCGCGAAGCGCCCGGGTGTATCAAGTTTTGAGACGTTAGTAAATAGCT
Hi-MC-153-con-replica	AGGTATGCGTTCAAGGACAGGGTGTATCAAGTTTTGAGACGTTAGTAAATAGCT
Hi-MC-154	ACGGAGATTTAGGAATATAAGAGCATTTCGGGGCC
Hi-MC-155	ATTGTGTGAATCTTACCAACGACGGAATACCCAAA
Hi-MC-156	GCATACTACCTTTTTAACATTAATTTTAGGAGAAAATACTTCC
Hi-MC-157	CGTTTGTTTCCTGTGTGAACGAGCCTAATTGCACCTGTCCTT
Hi-MC-158	ACTAAATCGGCAAAATCCCTTATACTGAGTATGATATTCCTC
Hi-MC-159	TAAATTTTAACCAATAGGGGAACAAGTAACCGGGTT
Hi-MC-160	GTTAGGTAGAAAGATTCCCTCGTTAACAGTTACAG
Hi-MC-161	CAGCAGTATTAACACCGGGTCAGTCAATAGAAAATCAGGTCA
Hi-MC-162	CAAGGTTTGCCCCAGCAGGCGAAAACAGAACACCAGCATCCAC
HI-MC-163-T	TTT GGGTAAAGTT TTT
HI-MC-163-con-D-LNA	TTTTTGGGTAAAGTTCCCGTGGGGCGGC(LC)(LA)(LG)AATTACCTACCGGC
HI-MC-163-con-replica	TTTTTGGGTAAAGTTCGCCAGGATGGGACGGTGGGGACTCGGGCA
Hi-MC-164-T	TTT CTGGTCGCTT TTT
Hi-MC-164-con-driver	GCCCCACGGGCTGGTCGCTTGCGCCCAGTCTCTCCAAATCACAACGTACC
Hi-MC-164-con-replica	CATCCTGGCGCTGGTCGCTTGTAGACTGCGGCGGCGCCGGCCCGATCGCA
Hi-MC-165-T	TTT GTCACGATAG TTT
Hi-MC-165-con-D-LNA	GACTGGGCGCGTCACGATAGTTTTTTATAGCCGC(LG)(LC)(LT)ATCCGGCGA
Hi-MC-165-con-replica	CGCAGTCTACGTCACGATAGTTTTTTGCTCGTGCGCCCCGGGCGGC
HI-MC-166-T	TTT AAGTGCTGGC TTT
Hi-MC-167-T	TTT AATTTTAATT TTT
Hi-MC-168-T	TTT CTGGAAGAGT TTT
HI-MC-169-T	TTT TCATTTCAGC TTT
Hi-MC-170-T	TTT AGCATTCTAA TTT
Hi-MC-171-T	TTT AGAAATAATC TTT
HI-MC-172-T	TTT TTTCTGTGAA TTT
HI-MC-172-con-driver	CAGCCGATTCTTTCTGTGAAAGATGAGCACTGCGATCGGGCCGGCGCCGC
HI-MC-172-con-replica	GTCCGCCCGGTTTCTGTGAAGTGGCGCACGGGTACGTTGTGATTTGGAGA
Hi-MC-173-T	TTT GGCTTACAGA TTT
Hi-MC-173-con-D-LNA	TTTTTGGCTTACAGAGAATCGGCTGTGCC(LC)(LG)(LA)GTCCCCACCGTCC
Hi-MC-173-con-replica	TTTTTGGCTTACAGACCGGGCGGACGCCGGTAGGTAATTCTGGCC
Hi-MC-174-T	TTT TTTCCTCGTC TTT
Hi-MC-174-con-D-LNA	GTGCTCATCTTTTCCTCGTCTTTTTTGCCGCCCGGGGC(LG)(LC)(LA)CGAGC
Hi-MC-174-con-replica	CGTGCGCCACTTTCCTCGTCTTTTTTTCGCCGGATAGCGCGGCTAT
Nls-1	5'-P- AATTAATACGACTCACTATAGGGAGAGTTAGTTGATTATGAAGAGGTGTGTTGGAGTTATTGTTTTGG
Nls-2	5'-P-GAAGATTGTAATAGGTTTGCGAATTTTTCAGAAAACAGATATATCTGTTTTCTATCTGTT
Ls-1	5'-P-ATATATCTGTTTTCTGAAAAATTCGCAAACCTATTACAATCTTCCCAAAACAATAACTCCAACACACC
Ls-2	5'-P-TCTTCATAATCAACTAACTCTCCCTATAGTGAGTCGTATTAATTAAGGGTTCAGCGTGGG
Nls1-wopro	5'-P-GCAATACAATTCATACTTAGGGGAGAGTTAGTTGATTATGAAGAGGTGTGTTGGAGTTATTGTTTTGG
Ls2-wopro	5'-P-TCTTCATAATCAACTAACTCTCCCCTAAGTATGAATTGTATTGCAAGGGTTCAGCGTGGG
Molecular Beacon (MB)	FAM-UUUGCCUGAAAAAUUCGCAAA-BABCYL

Minimal staple mix

The minimal staple mix contains all the ODNs that are common to every origami used in this study. The ODNs, shipped as desalted and freeze-dried, are resuspended in MQ H_2O to a 100 µM concentration according to the specification provided by the synthesis company. For the minimal mix each staple is mixed in 1:1 ratio in order to obtain a pre-mix used as a starting point in the assembly of each origami to avoid the pipetting of each single staple over and over again for each origami. Usually, we prepare the mix with 4 µl of each staple to obtain a minimal mix that can be used to assemble maximally 10 origamis. The minimal staple mix can be prepared in advance and stored at 4 °C for up to 3 months or frozen at -20 °C up to one year.

Minimal staple mix	
Name	Sequence
Hi-MC-1	AAATTCAAAAATAATTCGCAACCCGTGAGGGGTTGC
Hi-MC-2	GAAAGGCTATCAGGTCATTGCCTGGCAAACACGGTTGAAGCCCCATGT
Hi-MC-3	TTCAACCGCCTGGCCCTGAGAGAGTCCAGGGTTGCATCTTCAG
Hi-MC-5	TTTATAAAACAGAGGTGTTGAAAGATAAGTTTCCGGTAAAAG
Hi-MC-6	GATATGCGCGAACTGATAGCCGTCCAAACGTCTTAAATCGCA
Hi-MC-7	GGGTCAAATCACCATCAATATGATACCCTGTTAGTAGCAGTTTCATTTT
Hi-MC-8	TGGCAACTAATGCAGATACATATATTATGCGATTTTAAAGAT
Hi-MC-9	AGCGTAAAACGACGGCCATTACGCGCTGCGCATCGGCCGCC
Hi-MC-10	GAATTGAATGGCTATTATTGAGGACCTTATTATTTTGCTGTT
Hi-MC-11	TAAAAAGGAGCGGGCGCTAGGGCGTAGCGGTCCCGGAAACCG
Hi-MC-12	TACTGCAACAGGAAAAAAATATAATGAGGGTATAACTGACTAA
Hi-MC-13	CTGATTACGAGGCATAGCCACATTCTCATTATACCAGTACGA
Hi-MC-14	AGCCCCCGATTTAGAGCTTGACGGACTATGGTTAGCGGTCAC
Hi-MC-15	CATATTTACATTGGCAGAAACCACAGAGCAAGAAAATACAAC
Hi-MC-16	CGGACTACAACGCCTGTCCCTCAGCTCAGTAGCTA
Hi-MC-17	CCTAACACTATCATAACATCAGTTTTGGGAAGAAAAATGCCC
Hi-MC-19	TAACGCCATATTTAACACAAAAATGAAACAAACCGGAATAA
Hi-MC-20	TAACCTTCCTGTAGCCAACATTAAGCCGCCGATAT
Hi-MC-21	GAAGAAGATTAATCATATGTACCCAGAGAATCGAAAGGCGA
Hi-MC-22	AAGGCTCCAACCATCGCCCACGCACGGGATCATATAGATCA
Hi-MC-23	GCAGGTGAGAAAGGCCGGAGACAGAGAAGCCTGGCATCTGATTCCCTCC
Hi-MC-24	TCGAATCGGAACCCTAAAGGGAGCACGTATATCCTCAAGAAT
Hi-MC-25	GTCGATCCCCGGGTACCGGAAGGGTGTTTGGAGGGCCATCGC
Hi-MC-26	ATACAGAGCCCACAAGACATATTCTAAAGAAATTGCGTCGGA
Hi-MC-27	TTTGCGTAACGATCTAACCGTACTAGTATAGCACG
Hi-MC-28	ACTAAACAAAGTACAACGGAGATTCGCGACCTACCCAAAAGGCTTCTAC
Hi-MC-29	AGGACTCTTTATACAAAGGAGAATATTGAGCCTCAGAGTGAC
Hi-MC-30	GGCGTGCAGCAAGCGGTCCACGCTGAGGCGGTACACCACCCAG
Hi-MC-31	CGTGGAAAGCCGGCGAACGTGGCGATGCGCCCCAGGCGATTA
Hi-MC-32	AGGGAAAGAGGCAAAAGAATACACACTGACCGACCAGGCAACTTTAATT
Hi-MC-33	CACAGACACTTTACAGAACTGTTATCCTCGAAATCTGTATCATCGCCTG
Hi-MC-34	TCAAATAATTACTAGAAAACAGAAACTGATTATTTGACGTCAA
Hi-MC-36	GAGTCAAATATCGCGTTTATTCATAGAATGGGCAA
Hi-MC-37	GCGAGCGGATTGCATCAACGAGAATTGGCCTGAAG
Hi-MC-38	GAATATAAGAATAAACACAGATTTTAGCGGAAATGGATTCACT
Hi-MC-39	GCGAAACATCGCCATTACACTAACTAAAGGTACTTGCGGAAC
Hi-MC-40	GCTCGAACGAACCACCATATCTAAGAAACGCGGCGTTTAAAG
Hi-MC-41	CAGGATCGCACATTCGCCTCACATGGAAGCATAAAGTGTCAT
Hi-MC-42	TCAATAAATTTTAAAAATAAGAGAGGCATAATGGTTTGAAATCCTT
Hi-MC-43	TTTTAAGAGGAAGCCCGATGCTTTAAATAAAATAA
Hi-MC-44	GCGTCTTTCCAGACGACGACAATAAACAACATGTT
Hi-MC-45	CTCATTCCAGTAGCGACAGAACAGTGCCTTGAGTA
Hi-MC-46	TAGTTAATTTCGCATAGGCTGGCTCATAAGGGGAGGTTATA
Hi-MC-47	ATCGTCAAAATCATAGGTCATTTGAGGAAGGTGCAGAAGGACG
Hi-MC-48	GTGGACGGGCTTTCCAGTCGGGAAGTTG
Hi-MC-49	GTACGGAAACATGTTAGAATAGATAAAGAAGATGATGAATAA

Hi-MC-50	AGCCTGAATCCCCCTCAAAAAGACTAGTACCTTTAATTGCAAT
Hi-MC-51	AGAATGTGAGCGAGTAACGTCTGGACGTTAATATTTTGCAA
Hi-MC-52	TGTAAAGCGCCTCCAGCGCGCATCACGGCGGATTGACCGTAATGGGTTG
Hi-MC-54	ATATTTAGGTTGGGTTATAACAAACAGATTAGAGCCCTAGGAG
Hi-MC-55	CGATTGAAAATATATTCGGTCGCTGGGAGTTTTCTAAGATA
Hi-MC-57	ATTTTTCGAGTAAACAGCTTGATAGGGTAGCCATCGTAAGA
Hi-MC-58	CAAGTAGCTTAGATTAAGTATATGTAATATCTCCTGCAAGCGC
Hi-MC-59	GCCTCCAATCGCAAGACACGAATTAGTATTAGAATATTTCGGT
Hi-MC-60	AATGACCATTAGCTATATTTTCATAAATTTTTTAACGTACG
Hi-MC-61	AAGTCCTCAGAACCGCCAAGCATTCAACTTTCAACAGTTAAAA
Hi-MC-63	AGCGCGAACGGCGAGCTGAAAAGGTTTATTTGCAGCCTTGA
Hi-MC-65	TTCCGAAGAGTCAATAGTTGGAAACTCAACAGAGGCGGTGGCG
Hi-MC-66	ACGCTTGAGCCCACGGAGAATTGAATTACCTTTTTTAAGAAT
Hi-MC-67	ATCCAGTAGGGCTTAATTTACAGATAAGCCCGGAACCGCAC
Hi-MC-68	AACCACAGACAGCCCTCCCGCCACGCCGTCGACCC
Hi-MC-69	ACAAGAATCCTTGAAAACGCTTCTGTCAAATAGAGCCAGCGCG
Hi-MC-70	GTACGGCCAACGCGCGGTTATACTGGGAAGGCGGGCCTATAA
Hi-MC-72	ACCCATGTAATTTAGGCAAACGATCTATCTTCATCTTTAGTC
Hi-MC-73	AGGCACCCTCATTTTCAGTAACACCTAAAGGAATTGTGAATC
Hi-MC-74	AACAACAAAAAACCAAAATAGCGAGAGGCTTTTGCAGTTTTA
Hi-MC-75	ATGATTCAACCGTTCTAGCTGATAGGTTGTAAATCATAAACTAAATAAT
Hi-MC-76	CTATCGAGGTGCCGTAAAGCACTATTAGAATAAAGTATCCAG
Hi-MC-78	CGATAAAACACTCATCTTTGACCCGGTCAATGACCTTCGGGCTTGGAAC
Hi-MC-79	TACAGTCACACGACCAGTTATCATGCAATAGTTTTTGTTAGA
Hi-MC-81	CGAAGCTTGCATGCCTGGACGGTGGCGATACGCCGCCACACC
Hi-MC-83	CCCGGTAAAATACGTAATGCCACTGCTTTTGTAACCGATCTCCAATCAG
Hi-MC-84	TTGAACCGCCACCCTCAAGTACAAAGTGAGAATAGAAATTTC
Hi-MC-85	TTAAGAAAGGAAGGGAAGAAAGCGCCACCACAGAGGGTAAAT
Hi-MC-86	AAACAAACAGTTCAGAAAAAAAGATTGATAAGAGGTCATTTCC
Hi-MC-87	GAATCACTGCATTCCACACAACATAATTGTTGGTTTTCCCAGTCAGGGA
Hi-MC-88	GACCCAGCGATTATACCAAGCGCGTAGCCGGTGACAAGCACCAGACAGG
Hi-MC-90	AAGGTGAATTTCACAATTGGCAAAAGTACATAAATCAAACGC
Hi-MC-91	AGTTTCGGATTCTCCGTGAACGCCATCGCATTAAATTTTAAAA
Hi-MC-92	AACCTCAATCGTCTGAATTATCATATTGAGTGAGAATATTGC
Hi-MC-94	ACCGTGTAGGTAAAGATTCAAAAGAGGATAATTGGGGCAGTAGATATTA
Hi-MC-95	GCAGAGGAAGAACGCAGCTAATGCAGAATACAATTGCATGATAGCA
Hi-MC-96	AATGTTTATCAACAATAAGTTGCTACGCAGTGTCACCACCTG
Hi-MC-97	CACAATATGCCAGGCAAGGCAAAGCATAAAGACACCCTCAG
Hi-MC-98	ATCAATTAATGCCGGAGAGGGTAGCTCAGAGAATTAGCGTCAACATAGC
Hi-MC-99	TTTCAAAATCTGAAATATTTGCGGATACAGTAACAGTAACCG
Hi-MC-100	GCCACGAAGGCACCAACCTAAAACACTTGCAGAGGCAGATAATTTGGAA
Hi-MC-101	GGGCCCAAATCAAGTTTTTTGGGGAACAGGAAACCTATGCAA
Hi-MC-103	AGCCTATTTTTGAGAGATCTACAACGGTAATGCATGTCGTATAAGTTA
Hi-MC-104	TCTCACCACCAATAATACAGAAGGCAGGTTTAACGTCAGCGT
Hi-MC-105	ACCGGAACCCATGTACCGGGATAGCTGAGACACGT
Hi-MC-106	ATATCGACAGTAACTGTTTCTGCCTTGGGGTGAATAATGAGCT
Hi-MC-107	GGCGTAAATTATCAAGAGTAATCTAACGAGGCAAGATTGAT
Hi-MC-108	ACGGGCCCACTACGTGAACCATCAATTTTAGAATGCCCATG
Hi-MC-109	TTTGTGCCAGCTGCATTGAGCTAACATTCAGCAGCTGGCGAT
Hi-MC-110	AACGTCTGGAATTAACATCCAATACCAAAAATTAGACGTTC
Hi-MC-111	CTGCAGCAAATGAAAAACACCTTGGGCGACATTATTTTAACG
Hi-MC-113	GCCAATTCGTAATCATGGTAAAGCCAATGAGTAATGAATTTG
Hi-MC-115	TTACCTCAGAAAAGTCACCTGATTTAGAACCTACCATACGAG
Hi-MC-116	TTAGCTGTCTTTCCTTAAGGCTTATATTTTGATCACCGGGTT
Hi-MC-118	AAGTTATGTAAATGCTGACTGAGCAAATACATGTCTTTATAAA
Hi-MC-119	TTACTTTAGTATCATATGTCAAAATTTCATCACGCTCATTAAT
Hi-MC-120	CGGCCCCATCCTAATTTACCTCCCGGGCAACAAAAATCATAAG
Hi-MC-121	TGAAAGAACGGGTATTAAGCAAGCAAATTCAATTATTCGAT
Hi-MC-122	GCACCGTATATACCAGACGACGATTTATTACAATAAAACGAACTA
Hi-MC-123	GACCGGATTTCGCACGTTTGCACCGAAGAAGTTTGCATCGGG

Hi-MC-124	CTACCTGAACAAGAAAATTGAAGCAGAAAATACCATTATATT
Hi-MC-126	GGTCACCATTACATACAAACTAATATCAAGAAAACAAACTC
Hi-MC-127	AATGCCTGAGTAATCTCATATCCTGTTTAGATACAAGC
Hi-MC-128	CCAAGTGTGATAAATAAGGATGAATAACAAAGATTCACCTTCT
Hi-MC-129	TGCAATCAAAAGAATAGCCCGAGAATTAGTATCCTCATCCAG
Hi-MC-130	CGATGACCATAAATCAAGAAGCAAGATGGCTTAGAGCTGTA
Hi-MC-131	CAATGAGTTTCGTCACCGAGCCACATTAGGATTGC
Hi-MC-132	ACGGGGTTCAAGTTTGCCTTTGAAACGCAATAATACTAACGA
Hi-MC-133	AGGGTCTTTACCCATCAGCTTTCTTGAATATAATGCTGTGTT
Hi-MC-134	ATCTCCTGTTTGATGGTGGTTCCGATCGGCCGGCAGGTTCAG
Hi-MC-138	ATTTGTATCGTTGCGCCGACAATGCGAAAGAGCCCAATAAC
Hi-MC-139	AAACGAGCTTCAAAGCGAATACTGCGAATTTATCACGCAAGGGACA
Hi-MC-140	CCTTAACAGTAATTCTACTAATAGAATACTTACATAAACGC
Hi-MC-141	AGGCAAGCCCAAACGCCAAAAGGAAAACATGCAGA
Hi-MC-143	GCTTGAGGACTAAAGACTTTTTCAGGAACGACCGATAGGTTTATCGAAT
Hi-MC-144	TTGTAGGGTTGAGTGTTGTTCCAGCGTTGTAAAAGCGCTCAT
Hi-MC-145	AGATTAAATTAAAATTAAGAACTACGTAACACTTCGCTAGT
Hi-MC-146	CGATACCGCACTCATCGGGAATCAGCCAAAGGGAAGGTTGAT
Hi-MC-148	TGACATCGATTAAGACTTTTAGAATTCATTTCAATTACTGCA
Hi-MC-152	TCTGGAATCGTCATAAATTAATTCTCCAACAGGTCAGGTTAG
Hi-MC-154	ACGGAGATTTAGGAATATAAGAGCATTTCGGGGCC
Hi-MC-155	ATTGTGTGAATCTTACCAACGACGGAATACCCAAA
Hi-MC-156	GCATACTACCTTTTTAACATTAATTTTAGGAGAAAATACTTCC
Hi-MC-157	CGTTTGTTTCCTGTGTGAACGAGCCTAATTGCACCTGTCCTT
Hi-MC-158	ACTAAATCGGCAAAATCCCTTATACTGAGTATGATATTCCTC
Hi-MC-159	TAAATTTTAACCAATAGGGGAACAAGTAACCGGGTT
Hi-MC-160	GTTAGGTAGAAAGATTCCCTCGTTAACAGTTACAG
Hi-MC-161	CAGCAGTATTAACACCGGGTCAGTCAATAGAAAATCAGGTCA
Hi-MC-162	CAAGGTTTGCCCCAGCAGGCGAAAACAGAACACCAGCATCCAC
HI-MC-166-T	TTT AAGTGCTGGC TTT
Hi-MC-167-T	TTT AATTTTAATT TTT
Hi-MC-168-T	TTT CTGGAAGAGT TTT
HI-MC-169-T	TTT TCATTTCAGC TTT
Hi-MC-170-T	TTT AGCATTCTAA TTT
Hi-MC-171-T	TTT AGAAATAATC TTT

Transcribable sequences (dsDNA-t)

The transcribable sequences are assembled from a subset of ODNs that need to be prepared before the origami can be assembled. All the required strands are ordered and shipped as HPLC purified and freeze-dried, and are resuspended in MQ H_2O according to the manufacturer data sheet to obtain a final concentration of 100 µM. The concentration of each ODN is than measured again to ensure the right concentration for each sequence. To prepare the transcribable linker it is necessary to combine the required ODNs for each specific transcribable DNA sequence in a 1:1 ratio in a final concentration of 5 µM in 1X T4 ligase buffer (40 mM Tris-HCl, 10 mM $MgCl_2$, 10 mM DTT, 0.5 mM ATP, pH 7.8) with addition of NaCl to 20 mM final concentration. The samples are annealed with a controlled temperature gradient in the thermocycler and subsequentially ligated by addition of T4 ligase (1µl of ligase [Thermo Scientific™ T4 DNA Ligase, 5 Weiss U/µL] on 100 µl ligation volume) at 15 °C for at least 2h. The samples are purified using 100 K Amicon® Ultra 0.5 ml 100 kDa size exclusion filter by spinning at 5000 rcf for 5 min to remove the not ligated ODNs. The buffer is exchanged to 1 x DNA buffer (10 mM Tris·HCl, 50 mM NaCl, 10 mM $MgCl_2$ at pH 7.5) by subsequentially spinning the same filter for 5 more times by adding 500 µl of buffer each time. The sample is recovered by inverting the filter in a clean tube and spinning at 3000 rcf for 5 min. Usually, we started with a total assembly mixture of 150 µl volume with 5 µM mixture of each ODN and we obtain 80-100 µl of the ligated and purified strand in 2 µM concentration.

Thermocycler program for the transcribable DNA strand		
Lid temperature 98 °C		
Initial T (°C)	End T (°C)	Rate (°C/min)
95		Hold (5 min)
95	60	35
60	15	0.6
15		Hold

Transcribable sequence for the Nanoengine (NE)

Transcribable sequence for the Nanoengine (NE)	
Name	Sequence
HI-MC-71-dsDNA-p	5'-CGGTCGCCACCGCTAATAATGGCAATATTTGCATCCGCCCACGCTGAACCCTT
Nls-1	5'-P- AATTAATACGACTCACTATAGGGAGAGTTAGTTGATTATGAAGAGGTGTGTTGGAGTTATTGTTTTGG
Nls-2	5'-P-GAAGATTGTAATAGGTTTGCGAATTTTTCAGAAAACAGATATATCTGTTTTCTATCTGTT
HI-MC-112-dsDNA-c	5'-P-TTGTCACAATTTCTGAGAGGTAGAAACCAATCAAACGCGAAAAGACACATTTGGGAGA
Hi-MC-4-dsDNA	5'-ATTAGCCAGCTATAAAAAATATCTACATTTAGACAAAACAGATAGAAAACAG
Ls-1	5'-P-ATATATCTGTTTTCTGAAAAATTCGCAAACCTATTACAATCTTCCCAAAACAATAACTCCAACACACC
Ls-2	5'-P-TCTTCATAATCAACTAACTCTCCCTATAGTGAGTCGTATTAATTAAGGGTTCAGCGTGGG
HI-MC-53-dsDNA-cp	5'-P-CGGATACGTAAAAAGCCTGCAGTATAAAGCCAAAACAGGGGATAACCGCCACCCCAGA

Transcribable sequence for the Nicked-nanoengine (nNE)

Transcribable sequence for the Nicked-nanoengine (nNE)	
Name	Sequence
HI-MC-71-dsDNA-p	5'-CGGTCGCCACCGCTAATAATGGCAATATTTGCATCCGCCCACGCTGAACCCTT
Nls-1	5'-P- AATTAATACGACTCACTATAGGGAGAGTTAGTTGATTATGAAGAGGTGTGTTGGAGTTATTGTTTTGG
Nls-2	5'-P-GAAGATTGTAATAGGTTTGCGAATTTTTCAGAAAACAGATATATCTGTTTTCTATCTGTT
Hi-MC-4-dsDNA	5'-ATTAGCCAGCTATAAAAAATATCTACATTTAGACAAAACAGATAGAAAACAG
Ls-1	5'-P-ATATATCTGTTTTCTGAAAAATTCGCAAACCTATTACAATCTTCCCAAAACAATAACTCCAACACACC
Ls-2	5'-P-TCTTCATAATCAACTAACTCTCCCTATAGTGAGTCGTATTAATTAAGGGTTCAGCGTGGG

Transcribable sequence for the Nicked-nanoengine (nNE) lacking the T7 promoter region

Transcribable sequence for the Nicked-nanoengine (nNE) lacking the T7 promoter region	
Name	Sequence
HI-MC-71-dsDNA-p	5'-CGGTCGCCACCGCTAATAATGGCAATATTTGCATCCGCCCACGCTGAACCCTT
Nls-1	5'-P- AATTAATACGACTCACTATAGGGAGAGTTAGTTGATTATGAAGAGGTGTGTTGGAGTTATTGTTTTGG
Nls1-wopro	5'-P-GCAATACAATTCATACTTAGGGGAGAGTTAGTTGATTATGAAGAGGTGTGTTGGAGTTATTGTTTTGG
Hi-MC-4-dsDNA	5'-ATTAGCCAGCTATAAAAAATATCTACATTTAGACAAAACAGATAGAAAACAG
Ls-1	5'-P-ATATATCTGTTTTCTGAAAAATTCGCAAACCTATTACAATCTTCCCAAAACAATAACTCCAACACACC
Ls2-wopro	5'-P-TCTTCATAATCAACTAACTCTCCCCTAAGTATGAATTGTATTGCAAGGGTTCAGCGTGGG

Transcribable sequence anchored to the origami only on the opposite side to where the HT-T7RNAP is attached

Transcribable sequence not attached next to the HT-T7RNAP	
Name	Sequence
Nls-1	5'-P- AATTAATACGACTCACTATAGGGAGAGTTAGTTGATTATGAAGAGGTGTGTTGGAGTTATTGTTTTGG
Nls-2	5'-P-GAAGATTGTAATAGGTTTGCGAATTTTTCAGAAAACAGATATATCTGTTTTCTATCTGTT
Hi-MC-4-dsDNA	5'-ATTAGCCAGCTATAAAAAATATCTACATTTAGACAAAACAGATAGAAAACAG
Ls-1	5'-P-ATATATCTGTTTTCTGAAAAATTCGCAAACCTATTACAATCTTCCCAAAACAATAACTCCAACACACC
Ls-2	5'-P-TCTTCATAATCAACTAACTCTCCCTATAGTGAGTCGTATTAATTAAGGGTTCAGCGTGGG

Transcribable sequence anchored to the origami only next to the HT-T7RNAP

Transcribable sequence attached only next to the HT-T7RNAP	
Name	Sequence
Nls-1	5'-P- AATTAATACGACTCACTATAGGGAGAGTTAGTTGATTATGAAGAGGTGTGTTGGAGTTATTGTTTTGG
Nls-2	5'-P-GAAGATTGTAATAGGTTTGCGAATTTTTCAGAAAACAGATATATCTGTTTTCTATCTGTT
HI-MC-71-dsDNA-p	5'-CGGTCGCCACCGCTAATAATGGCAATATTTGCATCCGCCCACGCTGAACCCTT
Ls-1	5'-P-ATATATCTGTTTTCTGAAAAATTCGCAAACCTATTACAATCTTCCCAAAACAATAACTCCAACACACC
Ls-2	5'-P-TCTTCATAATCAACTAACTCTCCCTATAGTGAGTCGTATTAATTAAGGGTTCAGCGTGGG

DNA origami scaffold

The circular, 7249 bases long, single-stranded DNA scaffold was purchased from Tilibit Nanosystems GmbH, type p7249 (M13mp18) 2 ml at 400 nM.

M13mp18, circular single stranded DNA type p7249
AATGCTACTACTATTAGTAGAATTGATGCCACCTTTTCAGCTCGCGCCCCAAATGAAAATATAGCTAAACAGGTTATTGACCATTTGCGAAATGTATC<br>TAATGGTCAAACTAAATCTACTCGTTCGCAGAATTGGGAATCAACTGTTATATGGAATGAAACTTCCAGACACCGTACTTTAGTTGCATATTTAAAACA<br>TGTTGAGCTACAGCATTATATTCAGCAATTAAGCTCTAAGCCATCCGCAAAAATGACCTCTTATCAAAAGGAGCAATTAAAGGTACTCTCTAATCCTG<br>ACCTGTTGGAGTTTGCTTCCGGTCTGGTTCGCTTTGAAGCTCGAATTAAAACGCGATATTTGAAGTCTTTCGGGCTTCCTCTTAATCTTTTTGATGCA<br>ATCCGCTTTGCTTCTGACTATAATAGTCAGGGTAAAGACCTGATTTTTGATTTATGGTCATTCTCGTTTTCTGAACTGTTTAAAGCATTTGAGGGGGAT<br>TCAATGAATATTTATGACGATTCCGCAGTATTGGACGCTATCCAGTCTAAACATTTTACTATTACCCCCTCTGGCAAAACTTCTTTTGCAAAAGCCTCT<br>CGCTATTTTGGTTTTTATCGTCGTCTGGTAAACGAGGGTTATGATAGTGTTGCTCTTACTATGCCTCGTAATTCCTTTTGGCGTTATGTATCTGCATTA<br>GTTGAATGTGGTATTCCTAAATCTCAACTGATGAATCTTTCTACCTGTAATAATGTTGTTCCGTTAGTTCGTTTTATTAACGTAGATTTTTCTTCCCAAC<br>GTCCTGACTGGTATAATGAGCCAGTTCTTAAAATCGCATAAGGTAATTCACAATGATTAAAGTTGAAATTAAACCATCTCAAGCCCAATTTACTACTCG<br>TTCTGGTGTTTCTCGTCAGGGCAAGCCTTATTCACTGAATGAGCAGCTTTGTTACGTTGATTTGGGTAATGAATATCCGGTTCTTGTCAAGATTACTC<br>TTGATGAAGGTCAGCCAGCCTATGCGCCTGGTCTGTACACCGTTCATCTGTCCTCTTTCAAAGTTGGTCAGTTCGGTTCCCTTATGATTGACCGTCT<br>GCGCCTCGTTCCGGCTAAGTAACATGGAGCAGGTCGCGGATTTCGACACAATTTATCAGGCGATGATACAAATCTCCGTTGTACTTTGTTTCGCGCT<br>TGGTATAATCGCTGGGGGTCAAAGATGAGTGTTTTAGTGTATTCTTTTGCCTCTTTCGTTTTAGGTTGGTGCCTTCGTAGTGGCATTACGTATTTTAC<br>CCGTTTAATGGAAACTTCCTCATGAAAAAGTCTTTAGTCCTCAAAGCCTCTGTAGCCGTTGCTACCCTCGTTCCGATGCTGTCTTTCGCTGCTGAGG<br>GTGACGATCCCGCAAAAGCGGCCTTTAACTCCCTGCAAGCCTCAGCGACCGAATATATCGGTTATGCGTGGGCGATGGTTGTTGTCATTGTCGGCG<br>CAACTATCGGTATCAAGCTGTTTAAGAAATTCACCTCGAAAGCAAGCTGATAAACCGATACAATTAAAGGCTCCTTTTGGAGCCTTTTTTTTGGAGAT<br>TTTCAACGTGAAAAAATTATTATTCGCAATTCCTTTAGTTGTTCCTTTCTATTCTCACTCCGCTGAAACTGTTGAAAGTTGTTTAGCAAAATCCCATACA<br>GAAAATTCATTTACTAACGTCTGGAAAGACGACAAAACTTTAGATCGTTACGCTAACTATGAGGGCTGTCTGTGGAATGCTACAGGCGTTGTAGTTT<br>GTACTGGTGACGAAACTCAGTGTTACGGTACATGGGTTCCTATTGGGCTTGCTATCCCTGAAAATGAGGGTGGTGGCTCTGAGGGTGGCGGTTCTG<br>AGGGTGGCGGTTCTGAGGGTGGCGGTACTAAACCTCCTGAGTACGGTGATACACCTATTCCGGGCTATACTTATATCAACCCTCTCGACGGCACTT<br>ATCCGCCTGGTACTGAGCAAAACCCCGCTAATCCTAATCCTTCTCTTGAGGAGTCTCAGCCTCTTAATACTTTCATGTTTCAGAATAATAGGTTCCGA<br>AATAGGCAGGGGGCATTAACTGTTTATACGGGCACTGTTACTCAAGGCACTGACCCCGTTAAAACTTATTACCAGTACACTCCTGTATCATCAAAAG<br>CCATGTATGACGCTTACTGGAACGGTAAATTCAGAGACTGCGCTTTCCATTCTGGCTTTAATGAGGATTTATTTGTTTGTGAATATCAAGGCCAATCG<br>TCTGACCTGCCTCAACCTCCTGTCAATGCTGGCGGCGGCTCTGGTGGTGGTTCTGGTGGCGGCTCTGAGGGTGGTGGCTCTGAGGGTGGCGGTT<br>CTGAGGGTGGCGGCTCTGAGGGAGGCGGTTCCGGTGGTGGCTCTGGTTCCGGTGATTTTGATTATGAAAAGATGGCAAACGCTAATAAGGGGGCT<br>ATGACCGAAAATGCCGATGAAAACGCGCTACAGTCTGACGCTAAAGGCAAACTTGATTCTGTCGCTACTGATTACGGTGCTGCTATCGATGGTTTCA<br>TTGGTGACGTTTCCGGCCTTGCTAATGGTAATGGTGCTACTGGTGATTTTGCTGGCTCTAATTCCCAAATGGCTCAAGTCGGTGACGGTGATAATTC<br>ACCTTTAATGAATAATTTCCGTCAATATTTACCTTCCCTCCCTCAATCGGTTGAATGTCGCCCTTTTGTCTTTGGCGCTGGTAAACCATATGAATTTTC<br>TATTGATTGTGACAAAATAAACTTATTCCGTGGTGTCTTTGCGTTTCTTTTATATGTTGCCACCTTTATGTATGTATTTTCTACGTTTGCTAACATACTG<br>CGTAATAAGGAGTCTTAATCATGCCAGTTCTTTTGGGTATTCCGTTATTATTGCGTTTCCTCGGTTTCCTTCTGGTAACTTTGTTCGGCTATCTGCTTA<br>CTTTTCTTAAAAAGGGCTTCGGTAAGATAGCTATTGCTATTTCATTGTTTCTTGCTCTTATTATTGGGCTTAACTCAATTCTTGTGGGTTATCTCTCTGA<br>TATTAGCGCTCAATTACCCTCTGACTTTGTTCAGGGTGTTCAGTTAATTCTCCCGTCTAATGCGCTTCCCTGTTTTTATGTTATTCTCTCTGTAAAGGC<br>TGCTATTTTCATTTTTGACGTTAAACAAAAAATCGTTTCTTATTTGGATTGGGATAAATAATATGGCTGTTTATTTTGTAACTGGCAAATTAGGCTCTGG<br>AAAGACGCTCGTTAGCGTTGGTAAGATTCAGGATAAAATTGTAGCTGGGTGCAAAATAGCAACTAATCTTGATTTAAGGCTTCAAAACCTCCCGCAA<br>GTCGGGAGGTTCGCTAAAACGCCTCGCGTTCTTAGAATACCGGATAAGCCTTCTATATCTGATTTGCTTGCTATTGGGCGCGGTAATGATTCCTACG<br>ATGAAAATAAAAACGGCTTGCTTGTTCTCGATGAGTGCGGTACTTGGTTTAATACCCGTTCTTGGAATGATAAGGAAAGACAGCCGATTATTGATTGG<br>TTTCTACATGCTCGTAAATTAGGATGGGATATTATTTTTCTTGTTCAGGACTTATCTATTGTTGATAAACAGGCGCGTTCTGCATTAGCTGAACATGTT<br>GTTTATTGTCGTCGTCTGGACAGAATTACTTTACCTTTTGTCGGTACTTTATATTCTCTTATTACTGGCTCGAAAATGCCTCTGCCTAAATTACATGTT<br>GGCGTTGTTAAATATGGCGATTCTCAATTAAGCCCTACTGTTGAGCGTTGGCTTTATACTGGTAAGAATTTGTATAACGCATATGATACTAAACAGGC<br>TTTTTCTAGTAATTATGATTCCGGTGTTTATTCTTATTTAACGCCTTATTTATCACACGGTCGGTATTTCAAACCATTAAATTTAGGTCAGAAGATGAAA<br>TTAACTAAAATATATTTGAAAAAGTTTTCTCGCGTTCTTTGTCTTGCGATTGGATTTGCATCAGCATTTACATATAGTTATATAACCCAACCTAAGCCG<br>GAGGTTAAAAAGGTAGTCTCTCAGACCTATGATTTTGATAAATTCACTATTGACTCTTCTCAGCGTCTTAATCTAAGCTATCGCTATGTTTTCAAGGAT<br>TCTAAGGGAAAATTAATTAATAGCGACGATTTACAGAAGCAAGGTTATTCACTCACATATATTGATTTATGTACTGTTTCCATTAAAAAAGGTAATTCAA<br>ATGAAATTGTTAAATGTAATTAATTTTGTTTTCTTGATGTTTGTTTCATCATCTTCTTTTGCTCAGGTAATTGAAATGAATAATTCGCCTCTGCGCGATTT<br>TGTAACTTGGTATTCAAAGCAATCAGGCGAATCCGTTATTGTTTCTCCCGATGTAAAAGGTACTGTTACTGTATATTCATCTGACGTTAAACCTGAAAA<br>TCTACGCAATTTCTTTATTTCTGTTTTACGTGCAAATAATTTTGATATGGTAGGTTCTAACCCTTCCATTATTCAGAAGTATAATCCAAACAATCAGGAT<br>TATATTGATGAATTGCCATCATCTGATAATCAGGAATATGATGATAATTCCGCTCCTTCTGGTGGTTTCTTTGTTCCGCAAAATGATAATGTTACTCAA<br>ACTTTTAAAATTAATAACGTTCGGGCAAAGGATTTAATACGAGTTGTCGAATTGTTTGTAAAGTCTAATACTTCTAAATCCTCAAATGTATTATCTATTG<br>ACGGCTCTAATCTATTAGTTGTTAGTGCTCCTAAAGATATTTTAGATAACCTTCCTCAATTCCTTTCAACTGTTGATTTGCCAACTGACCAGATATTGA<br>TTGAGGGTTTGATATTTGAGGTTCAGCAAGGTGATGCTTTAGATTTTTCATTTGCTGCTGGCTCTCAGCGTGGCACTGTTGCAGGCGGTGTTAATAC<br>TGACCGCCTCACCTCTGTTTTATCTTCTGCTGGTGGTTCGTTCGGTATTTTTAATGGCGATGTTTTAGGGCTATCAGTTCGCGCATTAAAGACTAATA<br>GCCATTCAAAAATATTGTCTGTGCCACGTATTCTTACGCTTTCAGGTCAGAAGGGTTCTATCTCTGTTGGCCAGAATGTCCCTTTTATTACTGGTCGT<br>GTGACTGGTGAATCTGCCAATGTAAATAATCCATTTCAGACGATTGAGCGTCAAAATGTAGGTATTTCCATGAGCGTTTTTCCTGTTGCAATGGCTGG<br>CGGTAATATTGTTCTGGATATTACCAGCAAGGCCGATAGTTTGAGTTCTTCTACTCAGGCAAGTGATGTTATTACTAATCAAAGAAGTATTGCTACAA<br>CGGTTAATTTGCGTGATGGACAGACTCTTTTACTCGGTGGCCTCACTGATTATAAAAACACTTCTCAGGATTCTGGCGTACCGTTCCTGTCTAAAATC<br>CCTTTAATCGGCCTCCTGTTTAGCTCCCGCTCTGATTCTAACGAGGAAAGCACGTTATACGTGCTCGTCAAAGCAACCATAGTACGCGCCCTGTAGC<br>GGCGCATTAAGCGCGGCGGGTGTGGTGGTTACGCGCAGCGTGACCGCTACACTTGCCAGCGCCCTAGCGCCCGCTCCTTTCGCTTTCTTCCCTTC<br>CTTTCTCGCCACGTTCGCCGGCTTTCCCCGTCAAGCTCTAAATCGGGGGCTCCCTTTAGGGTTCCGATTTAGTGCTTTACGGCACCTCGACCCCAA<br>AAAACTTGATTTGGGTGATGGTTCACGTAGTGGGCCATCGCCCTGATAGACGGTTTTTCGCCCTTTGACGTTGGAGTCCACGTTCTTTAATAGTGGA<br>CTCTTGTTCCAAACTGGAACAACACTCAACCCTATCTCGGGCTATTCTTTTGATTTATAAGGGATTTTGCCGATTTCGGAACCACCATCAAACAGGAT<br>TTTCGCCTGCTGGGGCAAACCAGCGTGGACCGCTTGCTGCAACTCTCTCAGGGCCAGGCGGTGAAGGGCAATCAGCTGTTGCCCGTCTCACTGGT<br>GAAAAGAAAAACCACCCTGGCGCCCAATACGCAAACCGCCTCTCCCCGCGCGTTGGCCGATTCATTAATGCAGCTGGCACGACAGGTTTCCCGAC<br>TGGAAAGCGGGCAGTGAGCGCAACGCAATTAATGTGAGTTAGCTCACTCATTAGGCACCCCAGGCTTTACACTTTATGCTTCCGGCTCGTATGTTGT<br>GTGGAATTGTGAGCGGATAACAATTTCACACAGGAAACAGCTATGACCATGATTACGAATTCGAGCTCGGTACCCGGGGATCCTCTAGAGTCGACC<br>TGCAGGCATGCAAGCTTGGCACTGGCCGTCGTTTTACAACGTCGTGACTGGGAAAACCCTGGCGTTACCCAACTTAATCGCCTTGCAGCACATCCC<br>CCTTTCGCCAGCTGGCGTAATAGCGAAGAGGCCCGCACCGATCGCCCTTCCCAACAGTTGCGCAGCCTGAATGGCGAATGGCGCTTTGCCTGGTT<br>TCCGGCACCAGAAGCGGTGCCGGAAAGCTGGCTGGAGTGCGATCTTCCTGAGGCCGATACTGTCGTCGTCCCCTCAAACTGGCAGATGCACGGTT<br>ACGATGCGCCCATCTACACCAACGTGACCTATCCCATTACGGTCAATCCGCCGTTTGTTCCCACGGAGAATCCGACGGGTTGTTACTCGCTCACAT<br>TTAATGTTGATGAAAGCTGGCTACAGGAAGGCCAGACGCGAATTATTTTTGATGGCGTTCCTATTGGTTAAAAAATGAGCTGATTTAACAAAAATTTA<br>ATGCGAATTTTAACAAAATATTAACGTTTACAATTTAAATATTTGCTTATACAATCTTCCTGTTTTTGGGGCTTTTCTGATTATCAACCGGGGTACATAT<br>GATTGACATGCTAGTTTTACGATTACCGTTCATCGATTCTCTTGTTTGCTCCAGACTCTCAGGCAATGACCTGATAGCCTTTGTAGATCTCTCAAAAAT<br>AGCTACCCTCTCCGGCATTAATTTATCAGCTAGAACGGTTGAATATCATATTGATGGTGATTTGACTGTCTCCGGCCTTTCTCACCCTTTTGAATCTTT<br>ACCTACACATTACTCAGGCATTGCATTTAAAATATATGAGGGTTCTAAAAATTTTTATCCTTGCGTTGAAATAAAGGCTTCTCCCGCAAAAGTATTACA<br>GGGTCATAATGTTTTTGGTACAACCGATTTAGCTTTATGCTCTGAGGCTTTATTGCTTAATTTTGCTAATTCTTTGCCTTGCCTGTATGATTTATTGGAT<br>GTT

General procedure for Origami assembly

Assembly of nano engine starts with the preparation of the minimal staple mix. The staple mix can be used for all the origami structures presented in this study and is advantageous because it spares the operator to pipet all the staples each time for each structure. It is recommended to prepare enough master mix for 5 to 10 origami assemblies each time a new master mix is prepared. To prepare the minimal staple mix all the ODNs listed in the table "minimal master mix" are combined 1:1 in one reaction tube. Usually, we prepared minimal mixes with 2.5 to 4.0 µl of each staple. The master mix can be stored at 4 °C for up to 3 months.

The second step in the origami assembly requires to have the structure specific transcribable sequence assembled, ligated and purified and in sufficient amount for the origami preparation. Usually, we obtained transcribable sequences in 2 µM concentrations and add about 10 µl for a typical origami assembly so to obtain a 5-fold excess of the sequence compared to the origami scaffold. Note that the volume of transcribable sequence that needs to be added depends from the concentration of the sequence itself and the addition of 10 µl described here should not be taken as a standard amount that can be used for each assembly.

The third step requires the preparation and sorting of the structure specific staples. Unmodified Staples have been purchased as desalted and freeze dried while the modified staples, like the biotinylated sequences have all been ordered as HPLC purified and freeze dried. All the ODNs are dissolved in MQ H_2O to a final concentration of 100 µM. The stock concentration of the ODNs is not crucial at this stage since the staples are added in 10-fold excess to the scaffold to ensure proper origami formation. Make sure that all the staples are available and in sufficient amount for the origami assembly. For a typica nano engine assembly 0.4 µl of each staple is required.

The fourth step requires the addition of the scaffold to the prepared origami assembly mix. The scaffold is always added in a 10-fold lower amount compared to each staple and 5-fold less than the transcribable sequence. Since the staples are mainly desalted, the addition of a large excess of those ODN to the scaffold, assures that the origami is properly formed with the staples of the correct length. In a typical assembly 10 µl of the 400 nM scaffold are added to obtain a final scaffold concentration of 13.3 nM.

The assembly mix is completed by addition of nano engine origami buffer (NEOB, final concentration: 5 mM Tris, 5 mM NaCl, 5 mM EDTA, 14 mM $MgCl_2$) and bringing the assembly mix to volume with MQ H_2O.

To thermally fold the origami into its shape the samples are placed in a thermocycler that heats the sample to 80 °C to remove all secondary structure that could potentially form in the scaffold or the DNA staples. From 80 °C the sample is relatively quickly cooled to 65 °C with a rate of 1 °C/min. Once the sample reached 65 °C the sample cools to 20 °C with a shallow thermal gradient of 0.05 °C/min (this step takes a17 h so it is advisable to run it overnight). From 20 °C the sample is quickly brought to 10 °C with 2 °C/s gradient and the thermocycler is paused at that temperature.

As a reference sample 1 µl of the origami sample is withdraw after the thermal annealing process and set aside to be used as a reference for an agarose gel after the purification step. To purify the structures from the excess staples the origamis are precipitated due to centrifugation in PEG buffer. The thermally annealed origamis are mixed 1:1 with a 2X Precipitation Buffer (5% PEG 8000 (wt/vol) 5 mM Tris, 1 mM EDTA and 505 mM NaCl), placed in a 1.5 ml reaction tube and spun at 16000 rcf at 20 °C for 30 min. The supernatant is removed by making sure not to touch the pellet with the pipet tip. The supernatant can be stored by freezing since it can potentially be used to recover the excess staples to use in a second round of origami assembly. To remove all the excess precipitation solution from the tube containing the pellet the walls of the reaction tube can be dried from the buffer by using small strips cut from whatman filter paper by being careful not to touch the pellet in the tube. The pellet is re-suspended with addition of NEOB, 10 µl for a typical origami assembly, and incubation at 25 °C with 1000 rpm shaking in an Eppendorf ThermoMixerC for at least 3 h to insure complete dissolvement

of the origami. After resuspension of the pellet the purification is confirmed by running the precipitated sample (usually 0.3 µl) against the not purified reference, 1 µl aliquot withdrawn after the thermal annealing on 1% agarose.

Standard assembly protocol used to prepare the nano-engine origamis for this study

- 57.2 µl of the minimal staple mix (0.4 µl of each staple, finale concentration 133 nM of each staple)
- ~10 µl of the transcribable sequence (estimate for 2 µM, final concentration 65 nM)
- 0.4 µl of each additional staple (100 µM stock, finale concentration 133 nM of each staple)
- 10 µl m13mp18 scaffold (400 nM stock, finale concentration 13 nM)
- 30 µl 10X NEOB
- Bring to final volume of 300 µl with MQ H_2O

Split the sample into 50 µl aliquots into 200 µl reaction tube so that the mixture is fully submerged into the thermoregulating element of the thermocycler. In our experience, the yield of origami was reduced when the sample was not completely covered by the thermoregulating metal block in the thermocycler.

The origami is folded with the following temperature gradient in the thermocycler:

Initial T (°C)	End T (°C)	Rate (°C/min)
80	65	1
65	20	0.05
20	10	120
10		Hold

After the thermal folding of the origami, the aliquots are combined in a 1.5 ml reaction tube and 1 µl of the mixture set aside as a reference. The origami sample is mixed with 300 µl 2X Precipitation Buffer and centrifugated at 16000 rcf for 30 min at 20 °C. Supernatant is removed but not discarded. The walls of the tube are further cleaned from buffer leftovers with strips cut from whatman filter paper to remove as much precipitation buffer as possible. The pellet is then resuspended by adding 10 µl of NEOB followed by incubation at 25 °C with shaking at 1000 rpm in an Eppendorf ThermomixerC for at least 3h.

After resuspension the purification is controlled by running 0.3 µl of the purified sample against 1 µl of the not purified reference sample probe on 1% agarose in 0.5x TAE buffer at 80V for 1 h and 15 min.

The purified sample usually shows a well-defined high intensity low mobility band that indicates the proper formation of the origami while the reference sample usually shows a broad, not defined high mobility smear due to the excess staples with just a faint, low mobility band that corresponds to the origami band.

The concentration of the structures is determined with measuring the absorbance at 260 nm with a Eppendorf BioPhotometer Plus. To obtain a reasonable absorbance value on the Photometer, 0.5 µl the sample are diluted 1:140 times in a total of 70 µl of NEOB. The usual final concentration of the origami is in the range of 200 – 300 nM.

All the Nanoengine assemblies follow the same assembly procedure as described; For convenience we report below a list of all the necessary components for each of the origami structures reported in this publication. (nanoengine lacking the dsDNA-t, nanoengine lacking chloroalkane linker, nanoengine, nicked-nanoengine, nicked-nanoengine lacking the T7 promoter region, nicked-nanoengine with dsDNA-t not connected next to HT-T7RNAP, nicked-nanoengine with dsDNA-t connected only next to HT-T7RNAP, nicked-nanoengine_soft, nicked-nanoengine for single molecule FRET, Driver, Single-strand-hinge Follower, Soft-hinge Follower, Follower)

Origami structure lacking the dsDNA-t, no transcribable sequence (NTS)

- Minimal staple mix
- Additional staples:

Biotinylated ODNs	
Name	Sequence
Hi-MC-18-bio(-3')	biotin GCTCA TATTCATTGCTCCAACCCAGCCGC
Hi-MC-64-bio(-3')	biotin GCGTT GTGTACAAACTTTGTAGCGAACGA
Hi-MC-18-bic(-5')	AAACCGAGAAAAACCGGA TGAG
Hi-MC-64-bic(-5')	TAGGCGAATAATGAACG AACG
Hi-MC-147-bio	biotin CTCCT GGATTTTCTTTAATGACGGAATATGGTTCTCAATCGAGTGAATAACCTTATAG
Hi-MC-135-biC147	ATACAGGAGGTTTAGTAATAGTTACTGTATG AGGAG
Hi-MC-35-bio	biotin GCACA GTTTCCATTAAACGTCAGCAGACAACAAAAGGAGCGCTA
Hi-MC-102-biC35	GCCCAGTGCCACGCTGATCAAACCTACCAGCTTACCGCCAGCATCTGAGGAA TGTGC

ODNs for the attachment of the Halo T7 RNAP	
Name	Sequence
Hi-MC-82-Halo	Halo (ligand O2) AAGCGATCTAGCCCTACATTCAGATGTCAGAGAAAGCGCACATT
Hi-MC-150-comp	TGCGACTATTATAGTCAAAATCAGAGGTTGATTGCTGGGGAAATA GCTAGATCGC

ODNs for the compliant hinge	
Name	Sequence
Hi-MC-56	CCGTTCCAGTAAGCCTGGATAGCGTCCA
Hi-MC-77	TGCCAGTACAAAAGGTAAAGTAATTCTGTCCAGAGAACCGAGAGCG
Hi-MC-80	TTTTCGAGCCAGTAATTTATCCCAATCC
Hi-MC-93	TCGTTGATGAGGTAATAGTAAAATGTTTAGAGTCATAC
Hi-MC-114	TCAGGTAATAAAAAGAAGTTTTGCCAGAGGGTACAGGA
Hi-MC-117	ATGGCTTGCATTTTCGGTCATAGTAAGCAGATAGCTAAACAG
Hi-MC-125	GTGTACTGACTGTAGCGCGTTAGTTACCAGAAGGACCTAATT
Hi-MC-136	CCCTTTTTAAGAAAAGCCCCCTTATTAG
Hi-MC-142	CCATATTATAAGAGAATATAAAGTACCGTACAAAACGAACAATTCA

Auxiliary structural ODNs	
Name	Sequence
Hi-MC-4	ATTAGCCAGCTATAAAAAATATCTACATTTAACAATTTCTG
Hi-MC-53	CTGCAGTATAAAGCCAAAACAGGGGATAACCGCCACCCCAGA
Hi-MC-71	CGGTCGCCACCGCTAATAATGGCAATATTTGCACGTAAAAAGC
Hi-MC-112	AGAGGTAGAAACCAATCAAACGCGAAAAGACACATTTGGGAGA
HI-MC-163-T	TTT GGGTAAAGTT TTT
Hi-MC-164-T	TTT CTGGTCGCTT TTT
Hi-MC-165-T	TTT GTCACGATAG TTT
HI-MC-172-T	TTT TTTCTGTGAA TTT
Hi-MC-173-T	TTT GGCTTACAGA TTT
Hi-MC-174-T	TTT TTTCCTCGTC TTT
Hi-MC-151	CGCACCAGGCGCTGCAAGGCGATTACGCCAGATCCGCT
Hi-MC-153	TGTATCAAGTTTTGAGACGTTAGTAAATAGCT
Hi-MC-89	GCTGATTGCCCTTCCCAGTGATAGATGGCAGCTTTCCGGCACGCCG
Hi-MC-149	TGCGAGGGAGACAAAAGCTGAACCTAAATCGTCGCTATTCCCTTAGCAAGCC
Hi-MC-62	TTAAGTAACATAATAAAAATTAACTTTGGAACAAGAGT
Hi-MC-137	GGGCGATCCAGAATACAGTGCCCGTAATAGTGAATATCAACGTAACAAA

- M13mp18 scaffold
- Buffer
- H_2O

Nanoengine lacking chloroalkane linker

- Minimal staple mix
- Transcribable sequence for the nanoengine
- Additional staples:

Biotinylated ODNs	
Name	Sequence
Hi-MC-18-bio(-3')	biotin GCTCA TATTCATTGCTCCAACCCAGCCGC
Hi-MC-64-bio(-3')	biotin GCGTT GTGTACAAACTTTGTAGCGAACGA
Hi-MC-18-bic(-5')	AAACCGAGAAAAACCGGA TGAG
Hi-MC-64-bic(-5')	TAGGCGAATAATGAACG AACG
Hi-MC-147-bio	biotin CTCCT GGATTTTCTTTAATGACGGAATATGGTTCTCAATCGAGTGAATAACCTTATAG
Hi-MC-135-biC147	ATACAGGAGGTTTAGTAATAGTTACTGTATG AGGAG
Hi-MC-35-bio	biotin GCACA GTTTCCATTAAACGTCAGCAGACAACAAAAGGAGCGCTA
Hi-MC-102-biC35	GCCCAGTGCCACGCTGATCAAACCTACCAGCTTACCGCCAGCATCTGAGGAA TGTGC

ODNs for the compliant hinge	
Name	Sequence
Hi-MC-56	CCGTTCCAGTAAGCCTGGATAGCGTCCA
Hi-MC-77	TGCCAGTACAAAAGGTAAAGTAATTCTGTCCAGAGAACCGAGAGCG
Hi-MC-80	TTTTCGAGCCAGTAATTTATCCCAATCC
Hi-MC-93	TCGTTGATGAGGTAATAGTAAAATGTTTAGAGTCATAC
Hi-MC-114	TCAGGTAATAAAAAGAAGTTTTGCCAGAGGGTACAGGA
Hi-MC-117	ATGGCTTGCATTTTCGGTCATAGTAAGCAGATAGCTAAACAG
Hi-MC-125	GTGTACTGACTGTAGCGCGTTAGTTACCAGAAGGACCTAATT
Hi-MC-136	CCCTTTTTAAGAAAAGCCCCCTTATTAG
Hi-MC-142	CCATATTATAAGAGAATATAAAGTACCGTACAAAACGAACAATTCA

Auxiliary structural ODNs	
Name	Sequence
HI-MC-163-T	TTT GGGTAAAGTT TTT
Hi-MC-164-T	TTT CTGGTCGCTT TTT
Hi-MC-165-T	TTT GTCACGATAG TTT
HI-MC-172-T	TTT TTTCTGTGAA TTT
Hi-MC-173-T	TTT GGCTTACAGA TTT
Hi-MC-174-T	TTT TTTCCTCGTC TTT
Hi-MC-151	CGCACCAGGCGCTGCAAGGCGATTACGCCAGATCCGCT
Hi-MC-153	TGTATCAAGTTTTGAGACGTTAGTAAATAGCT
Hi-MC-89	GCTGATTGCCCTTCCCAGTGATAGATGGCAGCTTTCCGGCACGCCG
Hi-MC-149	TGCGAGGGAGACAAAAGCTGAACCTAAATCGTCGCTATTCCCTTAGCAAGCC
Hi-MC-62	TTAAGTAACATAATAAAAATTAACTTTGGAACAAGAGT
Hi-MC-137	GGGCGATCCAGAATACAGTGCCCGTAATAGTGAATATCAACGTAACAAA

ODN for no chloroalkane modification	
Name	Sequence
Hi-MC-82	TGGGGAAATACCTACATTCAGATGTCAGAGAAAGCGCACATT
Hi-MC-150	TGCGACTATTATAGTCAAAATCAGAGGTTGATTGC

- M13mp18 scaffold
- Buffer
- H_2O

Nanoengine (NE)

- Minimal staple mix
- Transcribable sequence for the nanoengine
- Additional staples:

Biotinylated ODNs	
Name	Sequence
Hi-MC-18-bio(-3')	biotin GCTCA TATTCATTGCTCCAACCCAGCCGC
Hi-MC-64-bio(-3')	biotin GCGTT GTGTACAAACTTTGTAGCGAACGA
Hi-MC-18-bic(-5')	AAACCGAGAAAAACCGGA TGAG
Hi-MC-64-bic(-5')	TAGGCGAATAATGAACG AACG
Hi-MC-147-bio	biotin CTCCT GGATTTTCTTTAATGACGGAATATGGTTCTCAATCGAGTGAATAACCTTATAG
Hi-MC-135-biC147	ATACAGGAGGTTTAGTAATAGTTACTGTATG AGGAG
Hi-MC-35-bio	biotin GCACA GTTTCCATTAAACGTCAGCAGACAACAAAAGGAGCGCTA
Hi-MC-102-biC35	GCCCAGTGCCACGCTGATCAAACCTACCAGCTTACCGCCAGCATCTGAGGAA TGTGC

ODNs for the attachment of the Halo T7 RNAP	
Name	Sequence
Hi-MC-82-Halo	Halo (ligand O2) AAGCGATCTAGCCCTACATTCAGATGTCAGAGAAAGCGCACATT
Hi-MC-150-comp	TGCGACTATTATAGTCAAAATCAGAGGTTGATTGCTGGGGAAATA GCTAGATCGC

ODNs for the compliant hinge	
Name	Sequence
Hi-MC-56	CCGTTCCAGTAAGCCTGGATAGCGTCCA
Hi-MC-77	TGCCAGTACAAAAGGTAAAGTAATTCTGTCCAGAGAACCGAGAGCG
Hi-MC-80	TTTTCGAGCCAGTAATTTATCCCAATCC
Hi-MC-93	TCGTTGATGAGGTAATAGTAAAATGTTTAGAGTCATAC
Hi-MC-114	TCAGGTAATAAAAAGAAGTTTTGCCAGAGGGTACAGGA
Hi-MC-117	ATGGCTTGCATTTTCGGTCATAGTAAGCAGATAGCTAAACAG
Hi-MC-125	GTGTACTGACTGTAGCGCGTTAGTTACCAGAAGGACCTAATT
Hi-MC-136	CCCTTTTTAAGAAAAGCCCCCTTATTAG
Hi-MC-142	CCATATTATAAGAGAATATAAAGTACCGTACAAAACGAACAATTCA

Auxiliary structural ODNs	
Name	Sequence
HI-MC-163-T	TTT GGGTAAAGTT TTT
Hi-MC-164-T	TTT CTGGTCGCTT TTT
Hi-MC-165-T	TTT GTCACGATAG TTT
HI-MC-172-T	TTT TTTCTGTGAA TTT
Hi-MC-173-T	TTT GGCTTACAGA TTT
Hi-MC-174-T	TTT TTTCCTCGTC TTT
Hi-MC-151	CGCACCAGGCGCTGCAAGGCGATTACGCCAGATCCGCT
Hi-MC-153	TGTATCAAGTTTTGAGACGTTAGTAAATAGCT
Hi-MC-89	GCTGATTGCCCTTCCCAGTGATAGATGGCAGCTTTCCGGCACGCCG
Hi-MC-149	TGCGAGGGAGACAAAAGCTGAACCTAAATCGTCGCTATTCCCTTAGCAAGCC
Hi-MC-62	TTAAGTAACATAATAAAAATTAACTTTGGAACAAGAGT
Hi-MC-137	GGGCGATCCAGAATACAGTGCCCGTAATAGTGAATATCAACGTAACAAA

- M13mp18 scaffold
- Buffer
- H_2O

Nicked-nanoengine (nNE)

- Minimal staple mix
- Transcribable sequence for the nicked-nanoengine
- Additional staples:

ODNs for the attachment of the transcribable nicked DNA strand

Name	Sequence
HI-MC-112-dsDNA-c	5'-P-TTGTCACAATTTCTGAGAGGTAGAAACCAATCAAACGCGAAAAGACACATTTGGGAGA
HI-MC-53-dsDNA-cp	5'-P-CGGATACGTAAAAAGCCTGCAGTATAAAGCCAAAACAGGGGATAACCGCCACCCCAGA

Biotinylated ODNs

Name	Sequence
Hi-MC-18-bio(-3')	biotin GCTCA TATTCATTGCTCCAACCCAGCCGC
Hi-MC-64-bio(-3')	biotin GCGTT GTGTACAAACTTTGTAGCGAACGA
Hi-MC-18-bic(-5')	AAACCGAGAAAAACCGGA TGAG
Hi-MC-64-bic(-5')	TAGGCGAATAATGAACG AACG
Hi-MC-147-bio	biotin CTCCT GGATTTTCTTTAATGACGGAATATGGTTCTCAATCGAGTGAATAACCTTATAG
Hi-MC-135-biC147	ATACAGGAGGTTTAGTAATAGTTACTGTATG AGGAG
Hi-MC-35-bio	biotin GCACA GTTTCCATTAAACGTCAGCAGACAACAAAAGGAGCGCTA
Hi-MC-102-biC35	GCCCAGTGCCACGCTGATCAAACCTACCAGCTTACCGCCAGCATCTGAGGAA TGTGC

ODNs for the compliant hinge

Name	Sequence
Hi-MC-56	CCGTTCCAGTAAGCCTGGATAGCGTCCA
Hi-MC-77	TGCCAGTACAAAAGGTAAAGTAATTCTGTCCAGAGAACCGAGAGCG
Hi-MC-80	TTTTCGAGCCAGTAATTTATCCCAATCC
Hi-MC-93	TCGTTGATGAGGTAATAGTAAAATGTTTAGAGTCATAC
Hi-MC-114	TCAGGTAATAAAAAGAAGTTTTGCCAGAGGGTACAGGA
Hi-MC-117	ATGGCTTGCATTTTCGGTCATAGTAAGCAGATAGCTAAACAG
Hi-MC-125	GTGTACTGACTGTAGCGCGTTAGTTACCAGAAGGACCTAATT
Hi-MC-136	CCCTTTTTAAGAAAAGCCCCCTTATTAG
Hi-MC-142	CCATATTATAAGAGAATATAAAGTACCGTACAAAACGAACAATTCA

Auxiliary structural ODNs

Name	Sequence
HI-MC-163-T	TTT GGGTAAAGTT TTT
Hi-MC-164-T	TTT CTGGTCGCTT TTT
Hi-MC-165-T	TTT GTCACGATAG TTT
HI-MC-172-T	TTT TTTCTGTGAA TTT
Hi-MC-173-T	TTT GGCTTACAGA TTT
Hi-MC-174-T	TTT TTTCCTCGTC TTT
Hi-MC-151	CGCACCAGGCGCTGCAAGGCGATTACGCCAGATCCGCT
Hi-MC-153	TGTATCAAGTTTTGAGACGTTAGTAAATAGCT
Hi-MC-89	GCTGATTGCCCTTCCCAGTGATAGATGGCAGCTTTCCGGCACGCCG
Hi-MC-149	TGCGAGGGAGACAAAAGCTGAACCTAAATCGTCGCTATTCCCTTAGCAAGCC
Hi-MC-62	TTAAGTAACATAATAAAAATTAACTTTGGAACAAGAGT
Hi-MC-137	GGGCGATCCAGAATACAGTGCCCGTAATAGTGAATATCAACGTAACAAA

ODNs for the attachment of the Halo T7 RNAP

Name	Sequence
Hi-MC-82-Halo	Halo (ligand O2) AAGCGATCTAGCCCTACATTCAGATGTCAGAGAAAGCGCACATT
Hi-MC-150-comp	TGCGACTATTATAGTCAAAATCAGAGGTTGATTGCTGGGGAAATA GCTAGATCGC

ODNs for the compliant hinge	
Name	Sequence
Hi-MC-56	CCGTTCCAGTAAGCCTGGATAGCGTCCA
Hi-MC-77	TGCCAGTACAAAAGGTAAAGTAATTCTGTCCAGAGAACCGAGAGCG
Hi-MC-80	TTTTCGAGCCAGTAATTTATCCCAATCC
Hi-MC-93	TCGTTGATGAGGTAATAGTAAAATGTTTAGAGTCATAC
Hi-MC-114	TCAGGTAATAAAAAGAAGTTTTGCCAGAGGGTACAGGA
Hi-MC-117	ATGGCTTGCATTTTCGGTCATAGTAAGCAGATAGCTAAACAG
Hi-MC-125	GTGTACTGACTGTAGCGCGTTAGTTACCAGAAGGACCTAATT
Hi-MC-136	CCCTTTTTAAGAAAAGCCCCCTTATTAG
Hi-MC-142	CCATATTATAAGAGAATATAAAGTACCGTACAAAACGAACAATTCA

- M13mp18 scaffold
- Buffer
- H_2O

Nicked-nanoengine lacking the T7 promoter region

- Minimal staple mix
- Transcribable sequence for the nicked-nanoengine lacking the T7 promoter region
- Additional staples:

ODNs for the attachment of the transcribable nicked DNA strand	
Name	Sequence
HI-MC-112-dsDNA-c	5'-P-TTGTCACAATTTCTGAGAGGTAGAAACCAATCAAACGCGAAAAGACACATTTGGGAGA
HI-MC-53-dsDNA-cp	5'-P-CGGATACGTAAAAAGCCTGCAGTATAAAGCCAAAACAGGGGATAACCGCCACCCCAGA

Biotinylated ODNs	
Name	Sequence
Hi-MC-18-bio(-3')	biotin GCTCA TATTCATTGCTCCAACCCAGCCGC
Hi-MC-64-bio(-3')	biotin GCGTT GTGTACAAACTTTGTAGCGAACGA
Hi-MC-18-bic(-5')	AAACCGAGAAAAACCGGA TGAG
Hi-MC-64-bic(-5')	TAGGCGAATAATGAACG AACG
Hi-MC-147-bio	biotin CTCCT GGATTTTCTTTAATGACGGAATATGGTTCTCAATCGAGTGAATAACCTTATAG
Hi-MC-135-biC147	ATACAGGAGGTTTAGTAATAGTTACTGTATG AGGAG
Hi-MC-35-bio	biotin GCACA GTTTCCATTAAACGTCAGCAGACAACAAAAGGAGCGCTA
Hi-MC-102-biC35	GCCCAGTGCCACGCTGATCAAACCTACCAGCTTACCGCCAGCATCTGAGGAA TGTGC

Auxiliary structural ODNs	
Name	Sequence
HI-MC-163-T	TTT GGGTAAAGTT TTT
Hi-MC-164-T	TTT CTGGTCGCTT TTT
Hi-MC-165-T	TTT GTCACGATAG TTT
HI-MC-172-T	TTT TTTCTGTGAA TTT
Hi-MC-173-T	TTT GGCTTACAGA TTT
Hi-MC-174-T	TTT TTTCCTCGTC TTT
Hi-MC-151	CGCACCAGGCGCTGCAAGGCGATTACGCCAGATCCGCT
Hi-MC-153	TGTATCAAGTTTTGAGACGTTAGTAAATAGCT
Hi-MC-89	GCTGATTGCCCTTCCCAGTGATAGATGGCAGCTTTCCGGCACGCCG
Hi-MC-149	TGCGAGGGAGACAAAAGCTGAACCTAAATCGTCGCTATTCCCTTAGCAAGCC
Hi-MC-62	TTAAGTAACATAATAAAAATTAACTTTGGAACAAGAGT
Hi-MC-137	GGGCGATCCAGAATACAGTGCCCGTAATAGTGAATATCAACGTAACAAA

ODNs for the attachment of the Halo T7 RNAP	
Name	Sequence
Hi-MC-82-Halo	Halo (ligand O2) AAGCGATCTAGCC CTACATTCAGATGTCAGAGAAAGCGCACATT
Hi-MC-150-comp	TGCGACTATTATAGTCAAAATCAGAGGTTGATTGCTGGGGAAATA GCTAGATCGC

ODNs for the compliant hinge	
Name	Sequence
Hi-MC-56	CCGTTCCAGTAAGCCTGGATAGCGTCCA
Hi-MC-77	TGCCAGTACAAAAGGTAAAGTAATTCTGTCCAGAGAACCGAGAGCG
Hi-MC-80	TTTTCGAGCCAGTAATTTATCCCAATCC
Hi-MC-93	TCGTTGATGAGGTAATAGTAAAATGTTTAGAGTCATAC
Hi-MC-114	TCAGGTAATAAAAAGAAGTTTTGCCAGAGGGTACAGGA
Hi-MC-117	ATGGCTTGCATTTTCGGTCATAGTAAGCAGATAGCTAAACAG
Hi-MC-125	GTGTACTGACTGTAGCGCGTTAGTTACCAGAAGGACCTAATT
Hi-MC-136	CCCTTTTTAAGAAAAGCCCCCTTATTAG
Hi-MC-142	CCATATTATAAGAGAATATAAAGTACCGTACAAAACGAACAATTCA

- M13mp18 scaffold
- Buffer
- H_2O

Nicked-nanoengine with dsDNA-t not connected next to HT-T7RNAP

- Minimal staple mix
- Transcribable sequence not attached next to the HT-T7RNAP
- Additional staples:

ODNs to complete the attachment of the transcribable DNA sequence	
Name	Sequence
HI-MC-112-dsDNA-c	5'-P-TTGTCACAATTTCTGAGAGGTAGAAACCAATCAAACGCGAAAAGACACATTTGGGAGA
Hi-MC-53	CTGCAGTATAAAGCCAAAACAGGGGATAACCGCCACCCCAGA
Hi-MC-71	CGGTCGCCACCGCTAATAATGGCAATATTTGCACGTAAAAAGC

Biotinylated ODNs	
Name	Sequence
Hi-MC-18-bio(-3')	biotin GCTCA TATTCATTGCTCCAACCCAGCCGC
Hi-MC-64-bio(-3')	biotin GCGTT GTGTACAAACTTTGTAGCGAACGA
Hi-MC-18-bic(-5')	AAACCGAGAAAAACCGGA TGAG
Hi-MC-64-bic(-5')	TAGGCGAATAATGAACG AACG
Hi-MC-147-bio	biotin CTCCT GGATTTTCTTTAATGACGGAATATGGTTCTCAATCGAGTGAATAACCTTATAG
Hi-MC-135-biC147	ATACAGGAGGTTTAGTAATAGTTACTGTATG AGGAG
Hi-MC-35-bio	biotin GCACA GTTTCCATTAAACGTCAGCAGACAACAAAAGGAGCGCTA
Hi-MC-102-biC35	GCCCAGTGCCACGCTGATCAAACCTACCAGCTTACCGCCAGCATCTGAGGAA TGTGC

Auxiliary structural ODNs	
Name	Sequence
HI-MC-163-T	TTT GGGTAAAGTT TTT
Hi-MC-164-T	TTT CTGGTCGCTT TTT
Hi-MC-165-T	TTT GTCACGATAG TTT
HI-MC-172-T	TTT TTTCTGTGAA TTT
Hi-MC-173-T	TTT GGCTTACAGA TTT
Hi-MC-174-T	TTT TTTCCTCGTC TTT
Hi-MC-151	CGCACCAGGCGCTGCAAGGCGATTACGCCAGATCCGCT
Hi-MC-153	TGTATCAAGTTTTGAGACGTTAGTAAATAGCT
Hi-MC-89	GCTGATTGCCCTTCCCAGTGATAGATGGCAGCTTTCCGGCACGCCG
Hi-MC-149	TGCGAGGGAGACAAAAGCTGAACCTAAATCGTCGCTATTCCCTTAGCAAGCC
Hi-MC-62	TTAAGTAACATAATAAAAATTAACTTTGGAACAAGAGT
Hi-MC-137	GGGCGATCCAGAATACAGTGCCCGTAATAGTGAATATCAACGTAACAAA

ODNs for the attachment of the Halo T7 RNAP	
Name	Sequence
Hi-MC-82-Halo	Halo (ligand O2) AAGCGATCTAGCC CTACATTCAGATGTCAGAGAAAGCGCACATT
Hi-MC-150-comp	TGCGACTATTATAGTCAAAATCAGAGGTTGATTGCTGGGGAAATA GCTAGATCGC

ODNs for the compliant hinge	
Name	Sequence
Hi-MC-56	CCGTTCCAGTAAGCCTGGATAGCGTCCA
Hi-MC-77	TGCCAGTACAAAAGGTAAAGTAATTCTGTCCAGAGAACCGAGAGCG
Hi-MC-80	TTTTCGAGCCAGTAATTTATCCCAATCC
Hi-MC-93	TCGTTGATGAGGTAATAGTAAAATGTTTAGAGTCATAC
Hi-MC-114	TCAGGTAATAAAAAGAAGTTTTGCCAGAGGGTACAGGA
Hi-MC-117	ATGGCTTGCATTTTCGGTCATAGTAAGCAGATAGCTAAACAG
Hi-MC-125	GTGTACTGACTGTAGCGCGTTAGTTACCAGAAGGACCTAATT
Hi-MC-136	CCCTTTTTAAGAAAAGCCCCCTTATTAG
Hi-MC-142	CCATATTATAAGAGAATATAAAGTACCGTACAAAACGAACAATTCA

- M13mp18 scaffold
- Buffer
- H_2O

Nicked-nanoengine with dsDNA-t connected only next to HT-T7RNAP

- Minimal staple mix
- Transcribable sequence attached only next to the HT-T7RNAP
- Additional staples:

ODNs to complete the attachment of the transcribable DNA sequence	
Name	Sequence
HI-MC-53-dsDNA-cp	5'-P-CGGATACGTAAAAAGCCTGCAGTATAAAGCCAAAACAGGGGATAACCGCCACCCCAGA
Hi-MC-4	ATTAGCCAGCTATAAAAAATATCTACATTTAACAATTTCTG
Hi-MC-112	AGAGGTAGAAACCAATCAAACGCGAAAAGACACATTTGGGAGA

Biotinylated ODNs	
Name	Sequence
Hi-MC-18-bio(-3')	biotin GCTCA TATTCATTGCTCCAACCCAGCCGC
Hi-MC-64-bio(-3')	biotin GCGTT GTGTACAAACTTTGTAGCGAACGA
Hi-MC-18-bic(-5')	AAACCGAGAAAAACCGGA TGAG
Hi-MC-64-bic(-5')	TAGGCGAATAATGAACG AACG
Hi-MC-147-bio	biotin CTCCT GGATTTTCTTTAATGACGGAATATGGTTCTCAATCGAGTGAATAACCTTATAG
Hi-MC-135-biC147	ATACAGGAGGTTTAGTAATAGTTACTGTATG AGGAG
Hi-MC-35-bio	biotin GCACA GTTTCCATTAAACGTCAGCAGACAACAAAAGGAGCGCTA
Hi-MC-102-biC35	GCCCAGTGCCACGCTGATCAAACCTACCAGCTTACCGCCAGCATCTGAGGAA TGTGC

Auxiliary structural ODNs	
Name	Sequence
HI-MC-163-T	TTT GGGTAAAGTT TTT
Hi-MC-164-T	TTT CTGGTCGCTT TTT
Hi-MC-165-T	TTT GTCACGATAG TTT
HI-MC-172-T	TTT TTTCTGTGAA TTT
Hi-MC-173-T	TTT GGCTTACAGA TTT
Hi-MC-174-T	TTT TTTCCTCGTC TTT
Hi-MC-151	CGCACCAGGCGCTGCAAGGCGATTACGCCAGATCCGCT
Hi-MC-153	TGTATCAAGTTTTGAGACGTTAGTAAATAGCT
Hi-MC-89	GCTGATTGCCCTTCCCAGTGATAGATGGCAGCTTTCCGGCACGCCG
Hi-MC-149	TGCGAGGGAGACAAAAGCTGAACCTAAATCGTCGCTATTCCCTTAGCAAGCC
Hi-MC-62	TTAAGTAACATAATAAAAATTAACTTTGGAACAAGAGT
Hi-MC-137	GGGCGATCCAGAATACAGTGCCCGTAATAGTGAATATCAACGTAACAAA

ODNs for the attachment of the Halo T7 RNAP	
Name	Sequence
Hi-MC-82-Halo	Halo (ligand O2) AAGCGATCTAGCC CTACATTCAGATGTCAGAGAAAGCGCACATT
Hi-MC-150-comp	TGCGACTATTATAGTCAAAATCAGAGGTTGATTGCTGGGGAAATA GCTAGATCGC

ODNs for the compliant hinge	
Name	Sequence
Hi-MC-56	CCGTTCCAGTAAGCCTGGATAGCGTCCA
Hi-MC-77	TGCCAGTACAAAAGGTAAAGTAATTCTGTCCAGAGAACCGAGAGCG
Hi-MC-80	TTTTCGAGCCAGTAATTTATCCCAATCC
Hi-MC-93	TCGTTGATGAGGTAATAGTAAAATGTTTAGAGTCATAC
Hi-MC-114	TCAGGTAATAAAAAGAAGTTTTGCCAGAGGGTACAGGA
Hi-MC-117	ATGGCTTGCATTTTCGGTCATAGTAAGCAGATAGCTAAACAG
Hi-MC-125	GTGTACTGACTGTAGCGCGTTAGTTACCAGAAGGACCTAATT
Hi-MC-136	CCCTTTTTAAGAAAAGCCCCCTTATTAG
Hi-MC-142	CCATATTATAAGAGAATATAAAGTACCGTACAAAACGAACAATTCA

- M13mp18 scaffold
- Buffer
- H_2O

Nicked-nanoengine_soft

- Minimal staple mix
- Transcribable sequence for the nicked-nanoengine
- Additional staples:

ODNs for soft-hinge	
Name	Sequence
Hi-MC-56	CCGTTCCAGTAAGCCTGGATAGCGTCCA
Hi-MC-77	TGCCAGTACAAAAGGTAAAGTAATTCTGTCCAGAGAACCGAGAGCG
Hi-MC-80	TTTTCGAGCCAGTAATTTATCCCAATCC
Hi-MC-93	TCGTTGATGAGGTAATAGTAAAATGTTTAGAGTCATAC
Hi-MC-114	TCAGGTAATAAAAAGAAGTTTTGCCAGAGGGTACAGGA
Hi-MC-136	CCCTTTTTAAGAAAAGCCCCCTTATTAG
Hi-MC-142	CCATATTATAAGAGAATATAAAGTACCGTACAAAACGAACAATTCA

ODNs for the attachment of the transcribable nicked DNA strand	
Name	Sequence
HI-MC-112-dsDNA-c	5'-P-TTGTCACAATTTCTGAGAGGTAGAAACCAATCAAACGCGAAAAGACACATTTGGGAGA
HI-MC-53-dsDNA-cp	5'-P-CGGATACGTAAAAAGCCTGCAGTATAAAGCCAAAACAGGGGATAACCGCCACCCCAGA

Biotinylated ODNs	
Name	Sequence
Hi-MC-18-bio(-3')	biotin GCTCA TATTCATTGCTCCAACCCAGCCGC
Hi-MC-64-bio(-3')	biotin GCGTT GTGTACAAACTTTGTAGCGAACGA
Hi-MC-18-bic(-5')	AAACCGAGAAAAACCGGA TGAG
Hi-MC-64-bic(-5')	TAGGCGAATAATGAACG AACG
Hi-MC-147-bio	biotin CTCCT GGATTTTCTTTAATGACGGAATATGGTTCTCAATCGAGTGAATAACCTTATAG
Hi-MC-135-biC147	ATACAGGAGGTTTAGTAATAGTTACTGTATG AGGAG
Hi-MC-35-bio	biotin GCACA GTTTCCATTAAACGTCAGCAGACAACAAAAGGAGCGCTA
Hi-MC-102-biC35	GCCCAGTGCCACGCTGATCAAACCTACCAGCTTACCGCCAGCATCTGAGGAA TGTGC

Auxiliary structural ODNs	
Name	Sequence
HI-MC-163-T	TTT GGGTAAAGTT TTT
Hi-MC-164-T	TTT CTGGTCGCTT TTT
Hi-MC-165-T	TTT GTCACGATAG TTT
HI-MC-172-T	TTT TTTCTGTGAA TTT
Hi-MC-173-T	TTT GGCTTACAGA TTT
Hi-MC-174-T	TTT TTTCCTCGTC TTT
Hi-MC-151	CGCACCAGGCGCTGCAAGGCGATTACGCCAGATCCGCT
Hi-MC-153	TGTATCAAGTTTTGAGACGTTAGTAAATAGCT
Hi-MC-89	GCTGATTGCCCTTCCCAGTGATAGATGGCAGCTTTCCGGCACGCCG
Hi-MC-149	TGCGAGGGAGACAAAAGCTGAACCTAAATCGTCGCTATTCCCTTAGCAAGCC
Hi-MC-62	TTAAGTAACATAATAAAAATTAACTTTGGAACAAGAGT
Hi-MC-137	GGGCGATCCAGAATACAGTGCCCGTAATAGTGAATATCAACGTAACAAA

ODNs for the attachment of the Halo T7 RNAP	
Name	Sequence
Hi-MC-82-Halo	Halo (ligand O2) AAGCGATCTAGCC CTACATTCAGATGTCAGAGAAAGCGCACATT
Hi-MC-150-comp	TGCGACTATTATAGTCAAAATCAGAGGTTGATTGCTGGGGAAATA GCTAGATCGC

- M13mp18 scaffold
- Buffer

- H_2O

Nicked-nanoengine for single molecule FRET

- Minimal staple mix
- Transcribable sequence for the nicked-nanoengine
- Additional staples:

ODNs for the attachment of the transcribable nicked DNA strand	
Name	Sequence
HI-MC-112-dsDNA-c	5'-P-TTGTCACAATTTCTGAGAGGTAGAAACCAATCAAACGCGAAAAGACACATTTGGGAGA
HI-MC-53-dsDNA-cp	5'-P-CGGATACGTAAAAAGCCTGCAGTATAAAGCCAAAACAGGGGATAACCGCCACCCCAGA

Biotinylated ODNs	
Name	Sequence
Hi-MC-147-bio	biotin CTCCT GGATTTTCTTTAATGACGGAATATGGTTCTCAATCGAGTGAATAACCTTATAG
Hi-MC-135-biC147	ATACAGGAGGTTTAGTAATAGTTACTGTATG AGGAG
Hi-MC-35-bio	biotin GCACA GTTTCCATTAAACGTCAGCAGACAACAAAAGGAGCGCTA
Hi-MC-102-biC35	GCCCAGTGCCACGCTGATCAAACCTACCAGCTTACCGCCAGCATCTGAGGAA TGTGC

ODNs for the compliant hinge	
Name	Sequence
Hi-MC-56	CCGTTCCAGTAAGCCTGGATAGCGTCCA
Hi-MC-77	TGCCAGTACAAAAGGTAAAGTAATTCTGTCCAGAGAACCGAGAGCG
Hi-MC-80	TTTTCGAGCCAGTAATTTATCCCAATCC
Hi-MC-93	TCGTTGATGAGGTAATAGTAAAATGTTTAGAGTCATAC
Hi-MC-114	TCAGGTAATAAAAAGAAGTTTTGCCAGAGGGTACAGGA
Hi-MC-117	ATGGCTTGCATTTTCGGTCATAGTAAGCAGATAGCTAAACAG
Hi-MC-125	GTGTACTGACTGTAGCGCGTTAGTTACCAGAAGGACCTAATT
Hi-MC-136	CCCTTTTTAAGAAAAGCCCCCTTATTAG
Hi-MC-142	CCATATTATAAGAGAATATAAAGTACCGTACAAAACGAACAATTCA

Auxiliary structural ODNs	
Name	Sequence
Hi-MC-18	AAACCGAGAAAAACCGGATATTCATTGCTCCAACCCAGCCGC
Hi-MC-64	TAGGCGAATAATGAACGGTGTACAAACTTTGTAGCGAACGA
HI-MC-163-T	TTT GGGTAAAGTT TTT
Hi-MC-164-T	TTT CTGGTCGCTT TTT
Hi-MC-165-T	TTT GTCACGATAG TTT
HI-MC-172-T	TTT TTTCTGTGAA TTT
Hi-MC-173-T	TTT GGCTTACAGA TTT
Hi-MC-174-T	TTT TTTCCTCGTC TTT
Hi-MC-151	CGCACCAGGCGCTGCAAGGCGATTACGCCAGATCCGCT
Hi-MC-153	TGTATCAAGTTTTGAGACGTTAGTAAATAGCT
Hi-MC-89	GCTGATTGCCCTTCCCAGTGATAGATGGCAGCTTTCCGGCACGCCG
Hi-MC-149	TGCGAGGGAGACAAAAGCTGAACCTAAATCGTCGCTATTCCCTTAGCAAGCC

ODNs for the attachment of the Halo T7 RNAP	
Name	Sequence
Hi-MC-82-Halo	Halo (ligand O2) AAGCGATCTAGCCCTACATTCAGATGTCAGAGAAAGCGCACATT
Hi-MC-150-comp	TGCGACTATTATAGTCAAAATCAGAGGTTGATTGCTGGGGAAATA GCTAGATCGC

ODNs for the single molecule FRET measurement	
Name	Sequence
Hi-MC-62 ExtCy5	TTAAGTAACATAATAAAAATTAACTTTGGAACAAGAGTCCACTAT-Cy5
Hi-MC-137 Ext Cy3	Cy3-TCTATCAGGGCGATCCAGAATACAGTGCCCGTAATAGTGAATATCAACGTAACAAA

- M13mp18 scaffold
- Buffer
- H_2O
- Shield from light by wrapping in aluminum foil

Driver

- Minimal staple mix
- Transcribable sequence for the nNE
- Additional staples:

Connecting ODN for the Driver	
Name	Sequence
HI-MC-163-con-D-LNA	TTTTTGGGTAAAGTTCCCGTGGGGCGGC(**LC)(LA)(LG)**AATTACCTACCGGC
Hi-MC-165-con-D-LNA	GACTGGGCGCGTCACGATAGTTTTTATAGCCGC(**LG)(LC)(LT)**ATCCGGCGA
Hi-MC-174-con-D-LNA	GTGCTCATCTTTTCCTCGTCTTTTTTGCCGCCCGGGGC(**LG)(LC)(LA)**CGAGC
Hi-MC-173-con-D-LNA	TTTTTGGCTTACAGAGAATCGGCTGTGCC(**LC)(LG)(LA)**GTCCCCACCGTCC
HI-MC-172-con-driver	CAGCCGATTCTTTCTGTGAAAGATGAGCACTGCGATCGGGCCGGCGCCGC
Hi-MC-151-con-drive	CGCACCAGGCGCTGCAAGGCGATTACGCCAGATCCGCTCAGCCCCGTCACGGTCGTGTTC
Hi-MC-153-con-drive	CGCGGCTCGCGAAGCGCCCGGGTGTATCAAGTTTTGAGACGTTAGTAAATAGCT
Hi-MC-89-con-drive	CTGTCCTTGAACGCATACCTCAGCTGATTGCCCTTCCCAGTGATAGATGGCAGCTTTCCGGCACGCCG
Hi-MC-149-con-drive	TGCGAGGGAGACAAAAGCTGAACCTAAATCGTCGCTATTCCCTTAGCAAGCCGTCGCGCGGCAGCTCCCGAGCA
Hi-MC-164-con-driver	GCCCCACGGGCTGGTCGCTTGCGCCCAGTCTCTCCAAATCACAACGTACC

(**L#** labelled oligos indicate locked nucleic acids)

Auxiliary structural ODNs	
Name	Sequence
Hi-MC-62	TTAAGTAACATAATAAAAATTAACTTTGGAACAAGAGT
Hi-MC-137	GGGCGATCCAGAATACAGTGCCCGTAATAGTGAATATCAACGTAACAAA

ODNs for the attachment of the transcribable nicked DNA strand	
Name	Sequence
HI-MC-112-dsDNA-c	5'-P-TTGTCACAATTTCTGAGAGGTAGAAACCAATCAAACGCGAAAAGACACATTTGGGAGA
HI-MC-53-dsDNA-cp	5'-P-CGGATACGTAAAAAGCCTGCAGTATAAAGCCAAAACAGGGGATAACCGCCACCCCAGA

Biotinylated ODNs	
Name	Sequence
Hi-MC-18-bio(-3')	biotin GCTCA TATTCATTGCTCCAACCCAGCCGC
Hi-MC-64-bio(-3')	biotin GCGTT GTGTACAAACTTTGTAGCGAACGA
Hi-MC-18-bic(-5')	AAACCGAGAAAAACCGGA TGAG
Hi-MC-64-bic(-5')	TAGGCGAATAATGAACG AACG
Hi-MC-147-bio	biotin CTCCT GGATTTTCTTTAATGACGGAATATGGTTCTCAATCGAGTGAATAACCTTATAG
Hi-MC-135-biC147	ATACAGGAGGTTTAGTAATAGTTACTGTATG AGGAG
Hi-MC-35-bio	biotin GCACA GTTTCCATTAAACGTCAGCAGACAACAAAAGGAGCGCTA
Hi-MC-102-biC35	GCCCAGTGCCACGCTGATCAAACCTACCAGCTTACCGCCAGCATCTGAGGAA TGTGC

ODNs for the attachment of the Halo T7 RNAP	
Name	Sequence
Hi-MC-82-Halo	Halo (ligand O2) AAGCGATCTAGCC CTACATTCAGATGTCAGAGAAAGCGCACATT
Hi-MC-150-comp	TGCGACTATTATAGTCAAAATCAGAGGTTGATTGCTGGGGAAATA GCTAGATCGC

ODNs for the compliant hinge	
Name	Sequence
Hi-MC-56	CCGTTCCAGTAAGCCTGGATAGCGTCCA
Hi-MC-77	TGCCAGTACAAAAGGTAAAGTAATTCTGTCCAGAGAACCGAGAGCG

Hi-MC-80	TTTTCGAGCCAGTAATTTATCCCAATCC
Hi-MC-93	TCGTTGATGAGGTAATAGTAAAATGTTTAGAGTCATAC
Hi-MC-114	TCAGGTAATAAAAAGAAGTTTTGCCAGAGGGTACAGGA
Hi-MC-117	ATGGCTTGCATTTTCGGTCATAGTAAGCAGATAGCTAAACAG
Hi-MC-125	GTGTACTGACTGTAGCGCGTTAGTTACCAGAAGGACCTAATT
Hi-MC-136	CCCTTTTTAAGAAAAGCCCCCTTATTAG
Hi-MC-142	CCATATTATAAGAGAATATAAAGTACCGTACAAAACGAACAATTCA

- M13mp18 scaffold
- Buffer
- H_2O

Follower

- Minimal staple mix
- Additional staples:

Double-strand hinge ODNs	
Name	Sequence
Hi-MC-117	ATGGCTTGCATTTTCGGTCATAGTAAGCAGATAGCTAAACAG
Hi-MC-125	GTGTACTGACTGTAGCGCGTTAGTTACCAGAAGGACCTAATT
Hi-MC-56	CCGTTCCAGTAAGCCTGGATAGCGTCCA
Hi-MC-136	CCCTTTTTAAGAAAAGCCCCCTTATTAG
Hi-MC-80	TTTTCGAGCCAGTAATTTATCCCAATCC
Hi-MC-93	TCGTTGATGAGGTAATAGTAAAATGTTTAGAGTCATAC
Hi-MC-142	CCATATTATAAGAGAATATAAAGTACCGTACAAAACGAACAATTCA
Hi-MC-77	TGCCAGTACAAAAGGTAAAGTAATTCTGTCCAGAGAACCGAGAGCG
Hi-MC-114	TCAGGTAATAAAAAGAAGTTTTGCCAGAGGGTACAGGA

Auxiliary structural Follower ODN	
Name	Sequence
Hi-MC-82	TGGGGAAATACCTACATTCAGATGTCAGAGAAAGCGCACATT
Hi-MC-150	TGCGACTATTATAGTCAAAATCAGAGGTTGATTGC
Hi-MC-4	ATTAGCCAGCTATAAAAAATATCTACATTTAACAATTTCTG
Hi-MC-53	CTGCAGTATAAAGCCAAAACAGGGGATAACCGCCACCCCAGA
Hi-MC-71	CGGTCGCCACCGCTAATAATGGCAATATTTGCACGTAAAAAGC
Hi-MC-112	AGAGGTAGAAACCAATCAAACGCGAAAAGACACATTTGGGAGA
Hi-MC-18	AAACCGAGAAAAACCGGATATTCATTGCTCCAACCCAGCCGC
Hi-MC-64	TAGGCGAATAATGAACGGTGTACAAACTTTGTAGCGAACGA
Hi-MC-147	AATGACGGAATATGGTTCTCAATCGAGTGAATAACCTTATAG
Hi-MC-35	ATCTGAGGAAGTTTCCATTAAACGTCAGCAGACAACAAAAGGAGCGCTA
Hi-MC-102	GCCCAGTGCCACGCTGATCAAACCTACCAGCTTACCGCCAGC
Hi-MC-135	ATACAGGAGGTTTAGTAATAGTTACTGTATGGGATTTTCTTT
Hi-MC-62	TTAAGTAACATAATAAAAATTAACTTTGGAACAAGAGT
Hi-MC-137	GGGCGATCCAGAATACAGTGCCCGTAATAGTGAATATCAACGTAACAAA

Connecting ODN for the Follower	
Name	Sequence
Hi-MC-151-con-replica	CGCACCAGGCGCTGCAAGGCGATTACGCCAGATCCGCTCATGCTCGGGAGCTGCCGCGCG
Hi-MC-153-con-replica	AGGTATGCGTTCAAGGACAGGGTGTATCAAGTTTTGAGACGTTAGTAAATAGCT
Hi-MC-89-con-replica	CGGGCGCTTCGCGAGCCGCGCAGCTGATTGCCCTTCCCAGTGATAGATGGCAGCTTTCCGGCACGCCG
Hi-MC-149-con-replica	TGCGAGGGAGACAAAAGCTGAACCTAAATCGTCGCTATTCCCTTAGCAAGCCGTGAACACGACCGTGACGGGGC
Hi-MC-174-con-replica	CGTGCGCCACTTTCCTCGTCTTTTTTTCGCCGGATAGCGCGGCTAT
HI-MC-172-con-replica	GTCCGCCCGGTTTCTGTGAAGTGGCGCACGGGTACGTTGTGATTTGGAGA
Hi-MC-173-con-replica	TTTTTGGCTTACAGACCGGGCGGACGCCGGTAGGTAATTCTGGCC
HI-MC-163-con-replica	TTTTTGGGTAAAGTTCGCCAGGATGGGACGGTGGGGACTCGGGCA
Hi-MC-164-con-replica	CATCCTGGCGCTGGTCGCTTGTAGACTGCGGCGGCGCCGGCCCGATCGCA
Hi-MC-165-con-replica	CGCAGTCTACGTCACGATAGTTTTTTGCTCGTGCGCCCCGGGCGGC

- M13mp18 scaffold
- Buffer
- H_2O

Follower with single-strand-hinge (ss-hinge)

- Minimal staple mix
- Additional staples:

Auxiliary structural Follower ODN	
Name	Sequence
Hi-MC-82	TGGGGAAATACCTACATTCAGATGTCAGAGAAAGCGCACATT
Hi-MC-150	TGCGACTATTATAGTCAAAATCAGAGGTTGATTGC
Hi-MC-4	ATTAGCCAGCTATAAAAAATATCTACATTTAACAATTTCTG
Hi-MC-53	CTGCAGTATAAAGCCAAAACAGGGGATAACCGCCACCCCAGA
Hi-MC-71	CGGTCGCCACCGCTAATAATGGCAATATTTGCACGTAAAAAGC
Hi-MC-112	AGAGGTAGAAACCAATCAAACGCGAAAAGACACATTTGGGAGA
Hi-MC-18	AAACCGAGAAAAACCGGATATTCATTGCTCCAACCCAGCCGC
Hi-MC-64	TAGGCGAATAATGAACGGTGTACAAACTTTGTAGCGAACGA
Hi-MC-147	AATGACGGAATATGGTTCTCAATCGAGTGAATAACCTTATAG
Hi-MC-35	ATCTGAGGAAGTTTCCATTAAACGTCAGCAGACAACAAAAGGAGCGCTA
Hi-MC-102	GCCCAGTGCCACGCTGATCAAACCTACCAGCTTACCGCCAGC
Hi-MC-135	ATACAGGAGGTTTAGTAATAGTTACTGTATGGGATTTTCTTT
Hi-MC-62	TTAAGTAACATAATAAAAATTAACTTTGGAACAAGAGT
Hi-MC-137	GGGCGATCCAGAATACAGTGCCCGTAATAGTGAATATCAACGTAACAAA

Connecting ODN for the Follower	
Name	Sequence
Hi-MC-151-con-replica	CGCACCAGGCGCTGCAAGGCGATTACGCCAGATCCGCTCATGCTCGGGAGCTGCCGCGCG
Hi-MC-153-con-replica	AGGTATGCGTTCAAGGACAGGGTGTATCAAGTTTTGAGACGTTAGTAAATAGCT
Hi-MC-89-con-replica	CGGGCGCTTCGCGAGCCGCGCAGCTGATTGCCCTTCCCAGTGATAGATGGCAGCTTTCCGGCACGCCG
Hi-MC-149-con-replica	TGCGAGGGAGACAAAAGCTGAACCTAAATCGTCGCTATTCCCTTAGCAAGCCGTGAACACGACCGTGACGGGGC
Hi-MC-174-con-replica	CGTGCGCCACTTTCCTCGTCTTTTTTTCGCCGGATAGCGCGGCTAT
HI-MC-172-con-replica	GTCCGCCCGGTTTCTGTGAAGTGGCGCACGGGTACGTTGTGATTTGGAGA
Hi-MC-173-con-replica	TTTTTGGCTTACAGACCGGGCGGACGCCGGTAGGTAATTCTGGCC
HI-MC-163-con-replica	TTTTTGGGTAAAGTTCGCCAGGATGGGACGGTGGGGACTCGGGCA
Hi-MC-164-con-replica	CATCCTGGCGCTGGTCGCTTGTAGACTGCGGCGGCGCCGGCCCGATCGCA
Hi-MC-165-con-replica	CGCAGTCTACGTCACGATAGTTTTTGCTCGTGCGCCCCGGGCGGC

- M13mp18 scaffold
- Buffer
- H_2O

Follower with soft-hinge

- Minimal staple mix
- Additional staples:

Soft-hinge ODNs	
Name	Sequence
Hi-MC-56	CCGTTCCAGTAAGCCTGGATAGCGTCCA
Hi-MC-136	CCCTTTTTAAGAAAAGCCCCCTTATTAG
Hi-MC-80	TTTTCGAGCCAGTAATTTATCCCAATCC
Hi-MC-93	TCGTTGATGAGGTAATAGTAAAATGTTTAGAGTCATAC
Hi-MC-142	CCATATTATAAGAGAATATAAAGTACCGTACAAAACGAACAATTCA
Hi-MC-77	TGCCAGTACAAAAGGTAAAGTAATTCTGTCCAGAGAACCGAGAGCG
Hi-MC-114	TCAGGTAATAAAAAGAAGTTTTGCCAGAGGGTACAGGA

Auxiliary structural Follower ODN	
Name	Sequence
Hi-MC-82	TGGGGAAATACCTACATTCAGATGTCAGAGAAAGCGCACATT
Hi-MC-150	TGCGACTATTATAGTCAAAATCAGAGGTTGATTGC
Hi-MC-4	ATTAGCCAGCTATAAAAAATATCTACATTTAACAATTTCTG
Hi-MC-53	CTGCAGTATAAAGCCAAAACAGGGGATAACCGCCACCCCAGA
Hi-MC-71	CGGTCGCCACCGCTAATAATGGCAATATTTGCACGTAAAAAGC
Hi-MC-112	AGAGGTAGAAACCAATCAAACGCGAAAAGACACATTTGGGAGA
Hi-MC-18	AAACCGAGAAAAACCGGATATTCATTGCTCCAACCCAGCCGC
Hi-MC-64	TAGGCGAATAATGAACGGTGTACAAACTTTGTAGCGAACGA
Hi-MC-147	AATGACGGAATATGGTTCTCAATCGAGTGAATAACCTTATAG
Hi-MC-35	ATCTGAGGAAGTTTCCATTAAACGTCAGCAGACAACAAAAGGAGCGCTA
Hi-MC-102	GCCCAGTGCCACGCTGATCAAACCTACCAGCTTACCGCCAGC
Hi-MC-135	ATACAGGAGGTTTAGTAATAGTTACTGTATGGGATTTTCTTT
Hi-MC-62	TTAAGTAACATAATAAAAATTAACTTTGGAACAAGAGT
Hi-MC-137	GGGCGATCCAGAATACAGTGCCCGTAATAGTGAATATCAACGTAACAAA

Connecting ODN for the Follower	
Name	Sequence
Hi-MC-151-con-replica	CGCACCAGGCGCTGCAAGGCGATTACGCCAGATCCGCTCATGCTCGGGAGCTGCCGCGCG
Hi-MC-153-con-replica	AGGTATGCGTTCAAGGACAGGGTGTATCAAGTTTTGAGACGTTAGTAAATAGCT
Hi-MC-89-con-replica	CGGGCGCTTCGCGAGCCGCGCAGCTGATTGCCCTTCCCAGTGATAGATGGCAGCTTTCCGGCACGCCG
Hi-MC-149-con-replica	TGCGAGGGAGACAAAAGCTGAACCTAAATCGTCGCTATTCCCTTAGCAAGCCGTGAACACGACCGTGACGGGGC
Hi-MC-174-con-replica	CGTGCGCCACTTTCCTCGTCTTTTTTTCGCCGGATAGCGCGGCTAT
HI-MC-172-con-replica	GTCCGCCCGGTTTCTGTGAAGTGGCGCACGGGTACGTTGTGATTTGGAGA
Hi-MC-173-con-replica	TTTTTGGCTTACAGACCGGGCGGACGCCGGTAGGTAATTCTGGCC
HI-MC-163-con-replica	TTTTTGGGTAAAGTTCGCCAGGATGGGACGGTGGGGACTCGGGCA
Hi-MC-164-con-replica	CATCCTGGCGCTGGTCGCTTGTAGACTGCGGCGGCGCCGGCCCGATCGCA
Hi-MC-165-con-replica	CGCAGTCTACGTCACGATAGTTTTTTGCTCGTGCGCCCCGGGCGGC

- M13mp18 scaffold
- Buffer
- H_2O
