# Supplementary material for: A rhythmically pulsing leaf-spring DNA-origami nanoengine that drives a passive follower
Source: Nat Nanotechnol. 2023 Oct 19;19(2):226–36. doi: 10.1038/s41565-023-01516-x (PMC10873200; doi:10.1038/s41565-023-01516-x)
Supplement: Supplementary file 5 — Graphical representation of the CadNano maps of the nanoengine origami. [file 41565_2023_1516_MOESM5_ESM.pdf]

a)

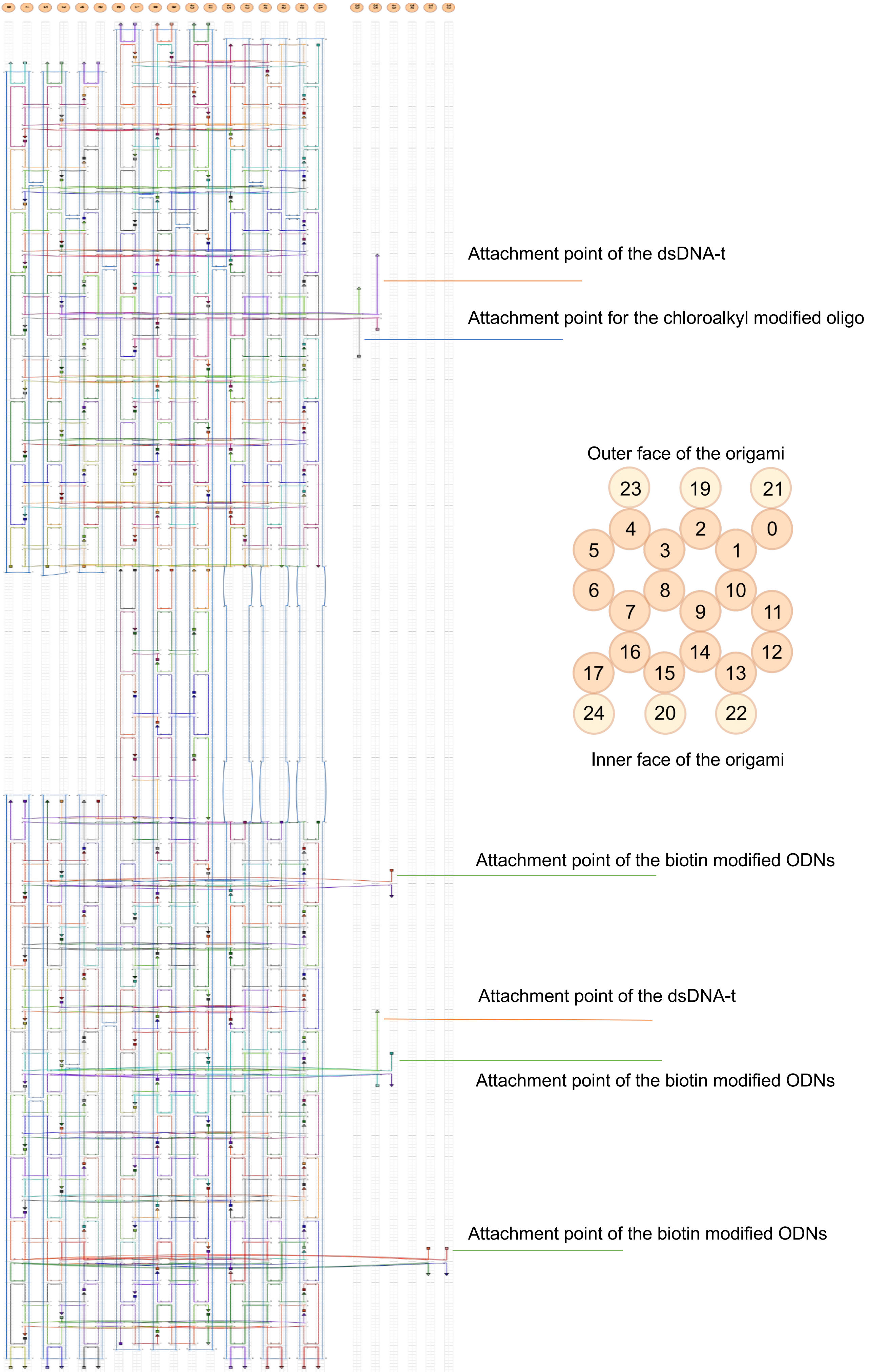
Attachment point of the dsDNA-t
Attachment point for the chloroalkyl modified oligo
Outer face of the origami
23 19 21
4 2 0
5 3 1
6 8 10
7 9 11
16 14 12
17 15 13
24 20 22
Inner face of the origami
Attachment point of the biotin modified ODNs
Attachment point of the dsDNA-t
Attachment point of the biotin modified ODNs
Attachment point of the biotin modified ODNs

b)

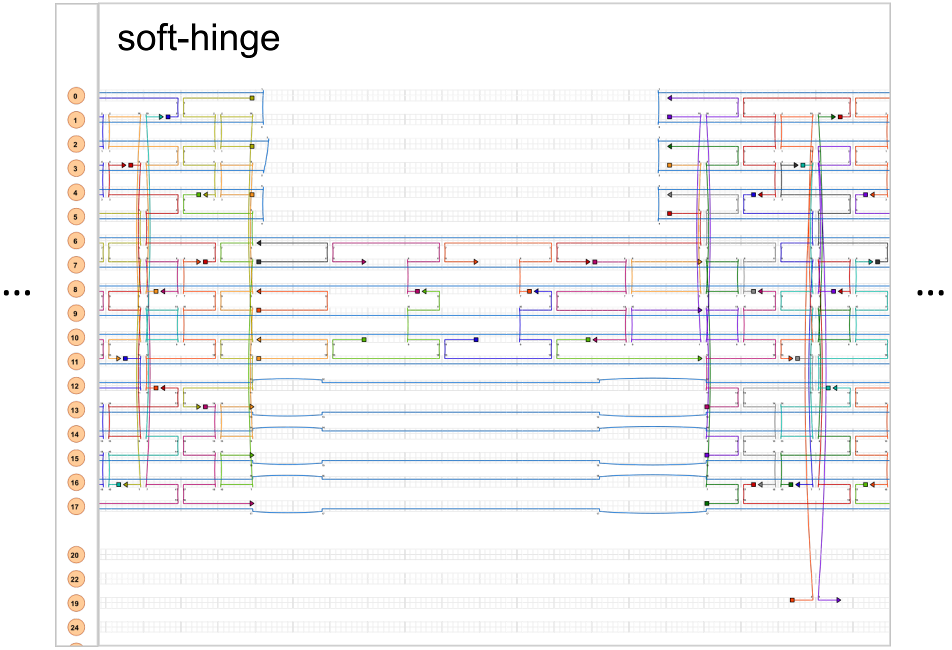

c)

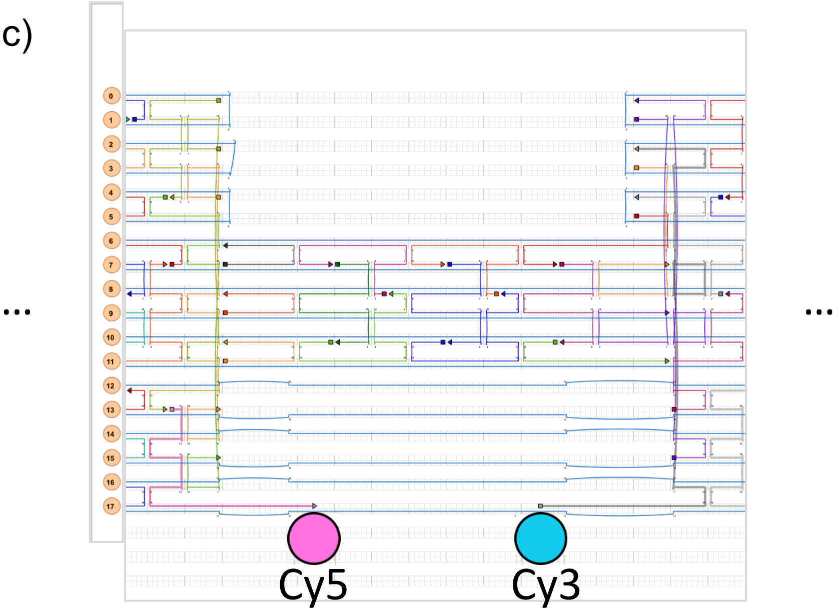

d)

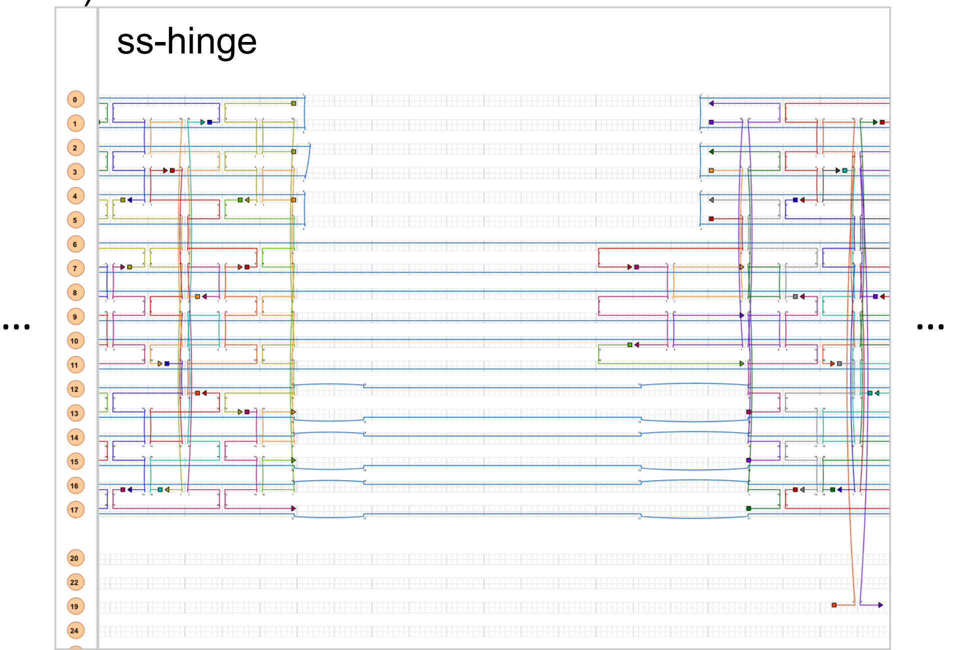

Graphical representation of the CadNano maps of the nanoengine origami (a). Cadnano map on the left nd side view of the 18-helix bundle honeycomb structure one the right. Staples that are represented to hybridize to the blue scaffold strand correspond to the staples that are necessary for the correct folding of the origami, while the sequences that protrude from the origami structure and don't hybridize to the scaffold are introduced as placeholder for the introduction of different functionalities to the origami. The helixes numbered from 0 to 17 are completely double-stranded within the stiff origami arms but not in the compliant hinge region in the centre. The helixes in the compliant hinge are completely double-stranded only for the helixes from 6 to 11 while the helixes 12 to 17 are left as single-stranded scaffold. The length of the helixes 6 – 11 in the hinge region are designed to be longer than the single-stranded scaffold strands in the same region, 84 and 53 nucleotides respectively. The helixes 0 to 5 are present only in the stiff arms and are interrupted in the hinge region. Helixes 19, 20, 21, 22, 23 and 24 don't represent real helixes but are used to facilitated the design of staples that are extended out of the origami. The attachment points for the transcribable DNA sequence extends out of the helix 13 pointing towards the helix 22 on the inside face of the origami. The chloroalkane modified ODN, for the attachment of the Halo T7 RNAP fusion protein extends from helix 15 towards the helix 20 on the inner face of the origami structure next to the transcribable DNA sequence. Biotinylated sequences, protrude towards the outer face of the origami from helix 0 to helix 21, from helix 2 to helix 19 and from helix 4 to helix 23. To change the theoretical stiffness of the hinge region we decided to make a soft hinge version (b, soft-hinge) by removing two staples, so to generate a hinge with only the helix 6 and 11 completely double-stranded while the strands 7, 8, 9 and 10 are interrupted twice by short single-stranded stretches. For single molecule FRET measurement a Version of the nicked-nanoengine with Cy3 as a donor and Cy5 an acceptor was created (c). The fluorophores have been added in the hinge region in correspondence of the single-stranded sequences and the staples that are carrying the dyes have been slightly extended towards the centre of the origami conveniently making it hybridization over 7 nt to one of the single stranded sequences. To remove all structural preorganization from the hinge region and generate a fully single stranded hinge we decided to remove all the 9 staples in the compliant area of the origami to make a structure with a completely single-stranded hinge(d, ss-hinge).
