# Supplementary material for: A rhythmically pulsing leaf-spring DNA-origami nanoengine that drives a passive follower
Source: Nat Nanotechnol. 2023 Oct 19;19(2):226–36. doi: 10.1038/s41565-023-01516-x (PMC10873200; doi:10.1038/s41565-023-01516-x)
Supplement: Supplementary file 6 — Glossary and detailed description of the various constructs I–X used in this study. [file 41565_2023_1516_MOESM6_ESM.pdf]

Glossary and detailed description of the various constructs **I-X** used in this study

I. Origami structure lacking the dsDNA-t, no transcribable sequence (NTS)

a)

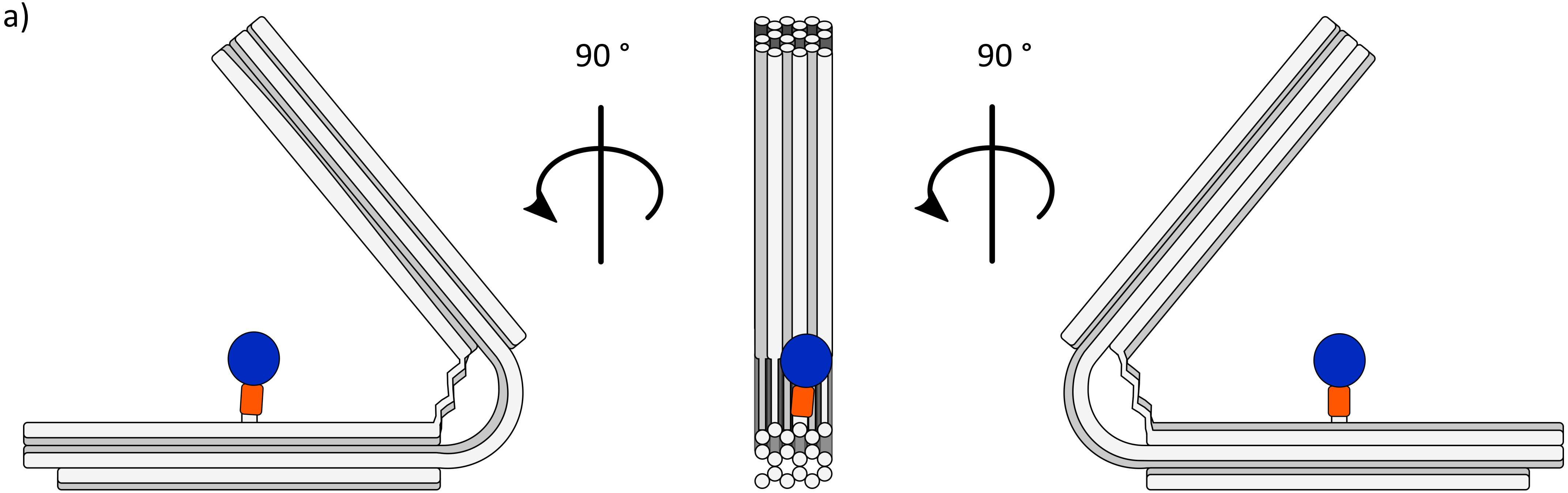

Nanoengine lacking transcribable template: a) Schematic representation of the origami structure. The origami is assembled without the transcribable dsDNA sequence. The chloroalkane modified sequence is present on the origami so that the HT-T7RNAP, represented in blue-organge can covalently bind to the origami. No transcription can take place because of the missing target sequence.

II. Double stranded template (dsDNA-t)

a)

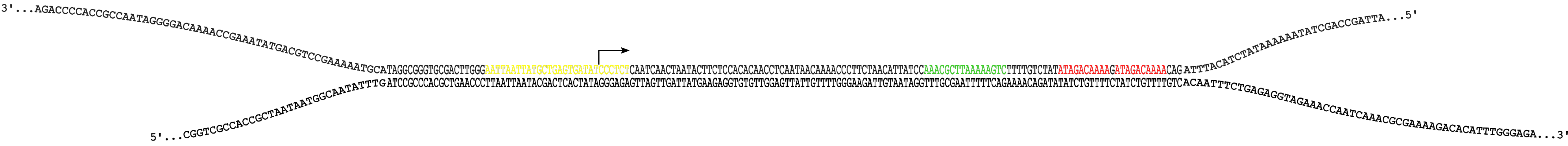

b)

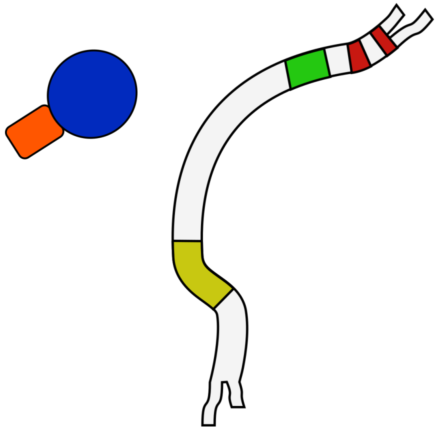

dsDNA: transcribable dsDNA sequence that is not attached to the origami. It has been used to test the intermolecular transcription efficiency. a) detailed sequence representation. The yellow sequence corresponds to the promoter region and the black arrow indicates the point of transcription initiation. The green sequence corresponds to the sequence that once transcribed is complementary to the molecular beacon (MB). In red two terminator sequences. The single stranded overhangs correspond to sequences that will embed into the origami and anchor the trasncribable dsDNA to the complete origami In b) schematic representation of the sequence presented in above with the HT7-RNAP next to it to indicate intermolecular interaction between the two.

III. Nanoengine lacking chloroalkane linker

a)

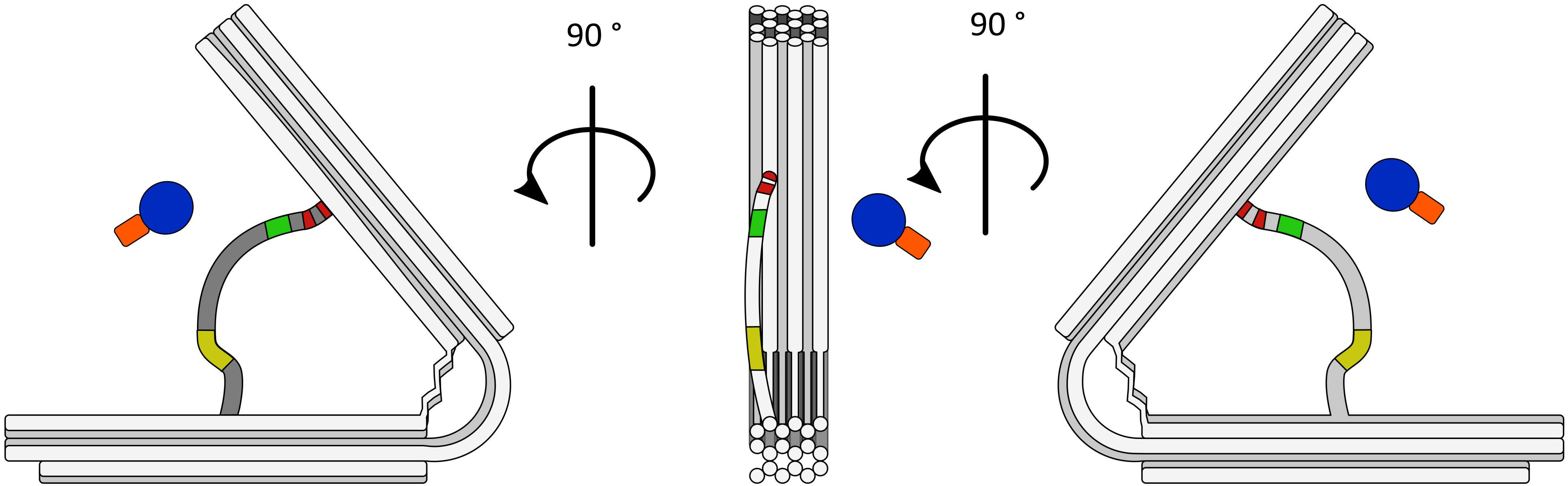

b)

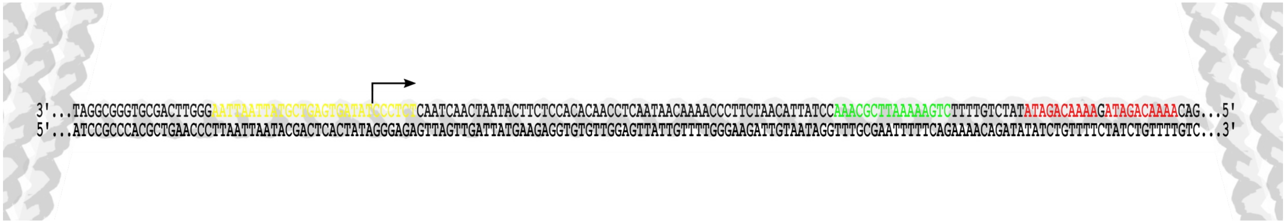

c)

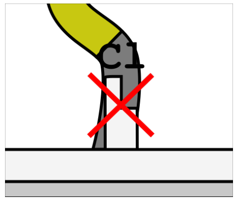

Missing Chloroalkane modified sequence

Nanoengine lacking chloroalkane linker. The structure is missing the chloroalkane modified oligo and does not allow to the covalent bond of the HT-T7RNAP to the origami. The schematic representation a) shows the origami structure complete with the HT-T7RNAP in a intermolecular fashion. b) detailed transcribable dsDNA sequence that is fully dsDNA and anchored at both ends with the origami. (promoter = yellow, MB complementary sequence = green, termitator sequneces =red). c) the chloroalkane modified ODN next to the transcribable sequence is fully absent from the origami

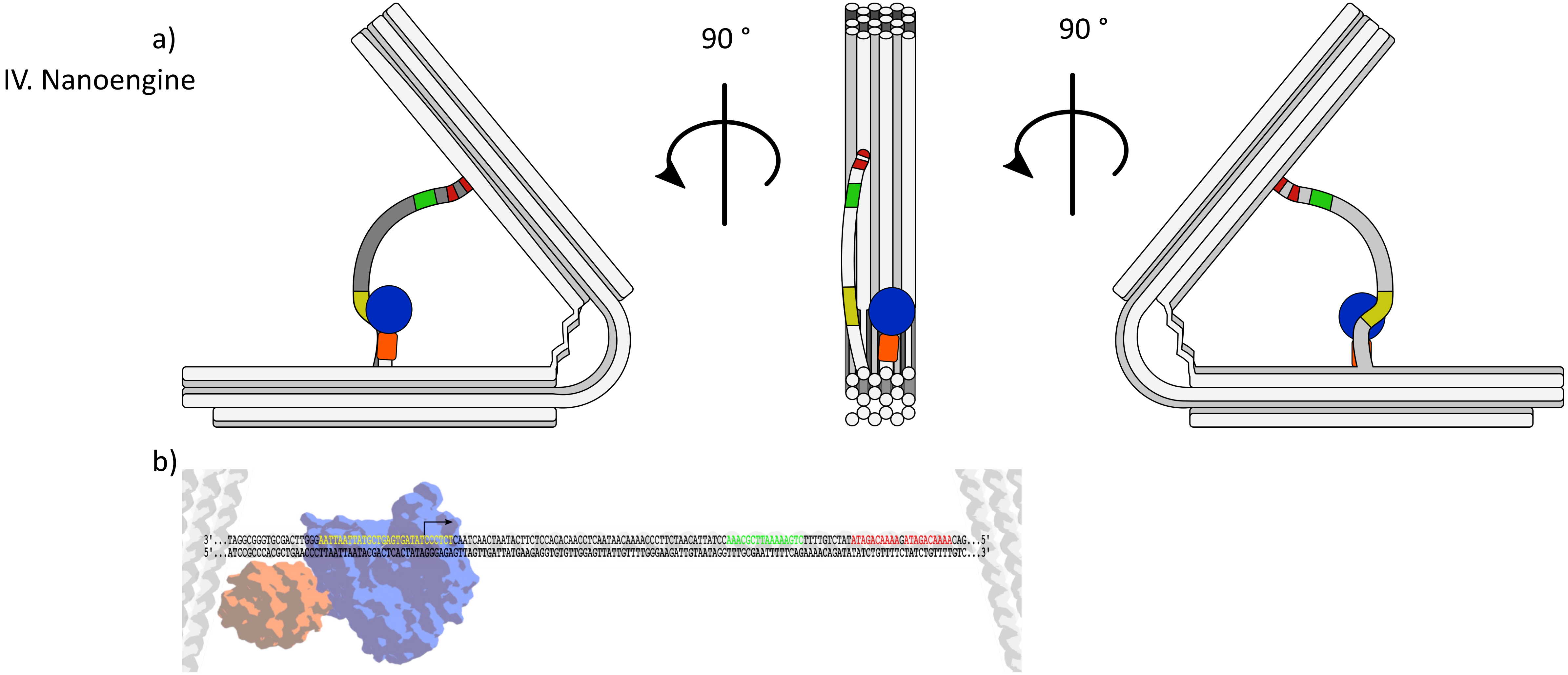

Fully assembled nanoengine (NE). a) schematic representation of the nanoengine complete with the transcribable dsDNA sequence and the HT-T7RNAP. The represented fully functional sequence is able to generate repetitive and autonomous opening and closing movement once fuel in the form of NTP is added. b) shows a detailed dsDNA sequence with a graphical representation of the HT7-RNAP (blue and orange) anchored in proximity of the promoter region depicted in yllow.

V. Nicked-nanoengine

a)

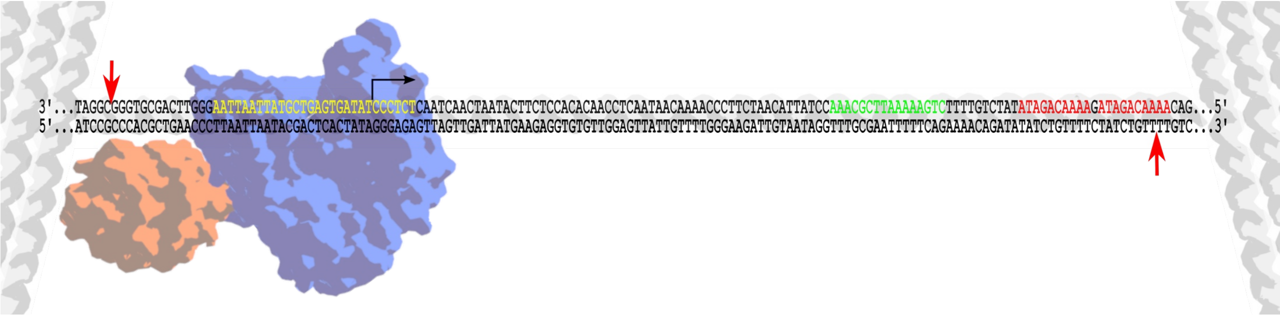

b)

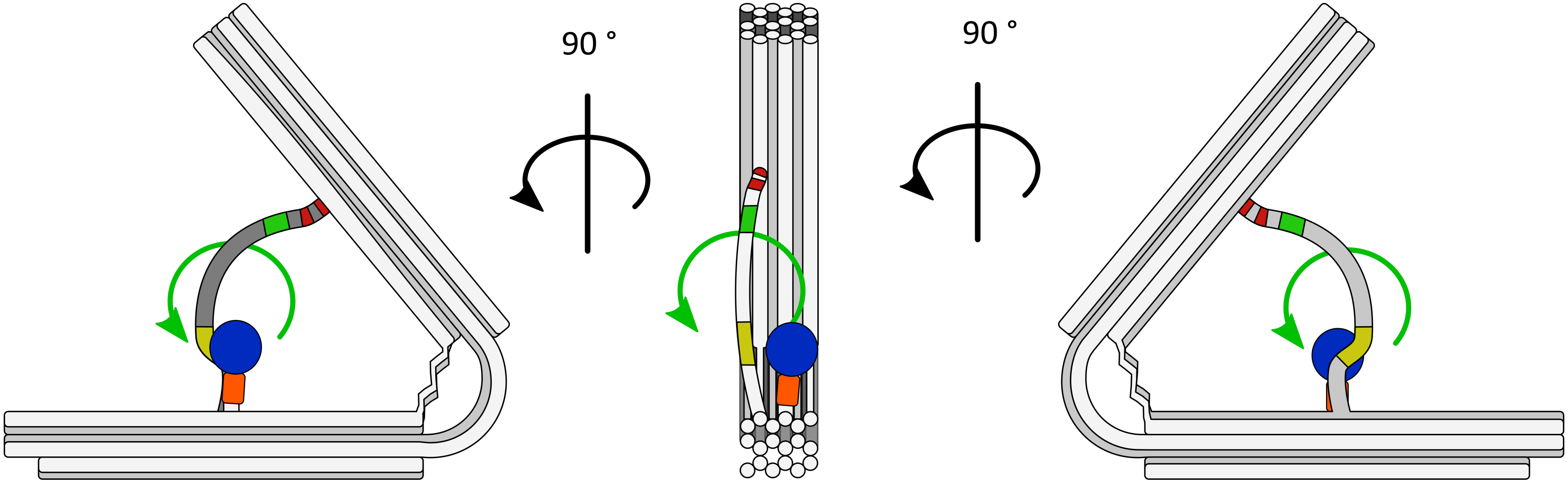

In the nicked nanoengine (nNE) two single stranded nicks are the transcribable dsDNA sequence. The nicks permit the dissipation of supercoiling that can be accumulated during transcription. The nicks are introduced close to where the transcribable sequence meets the Origami indicated with red arrows in a). The green arrow shown in the schematic representation in b) indicate the free spinning of the transcribable dsDNA sequence.

VI. Nicked-nanoengine + HT-T7RNAP preincubated with 1 equiv of the chloroalkane linker

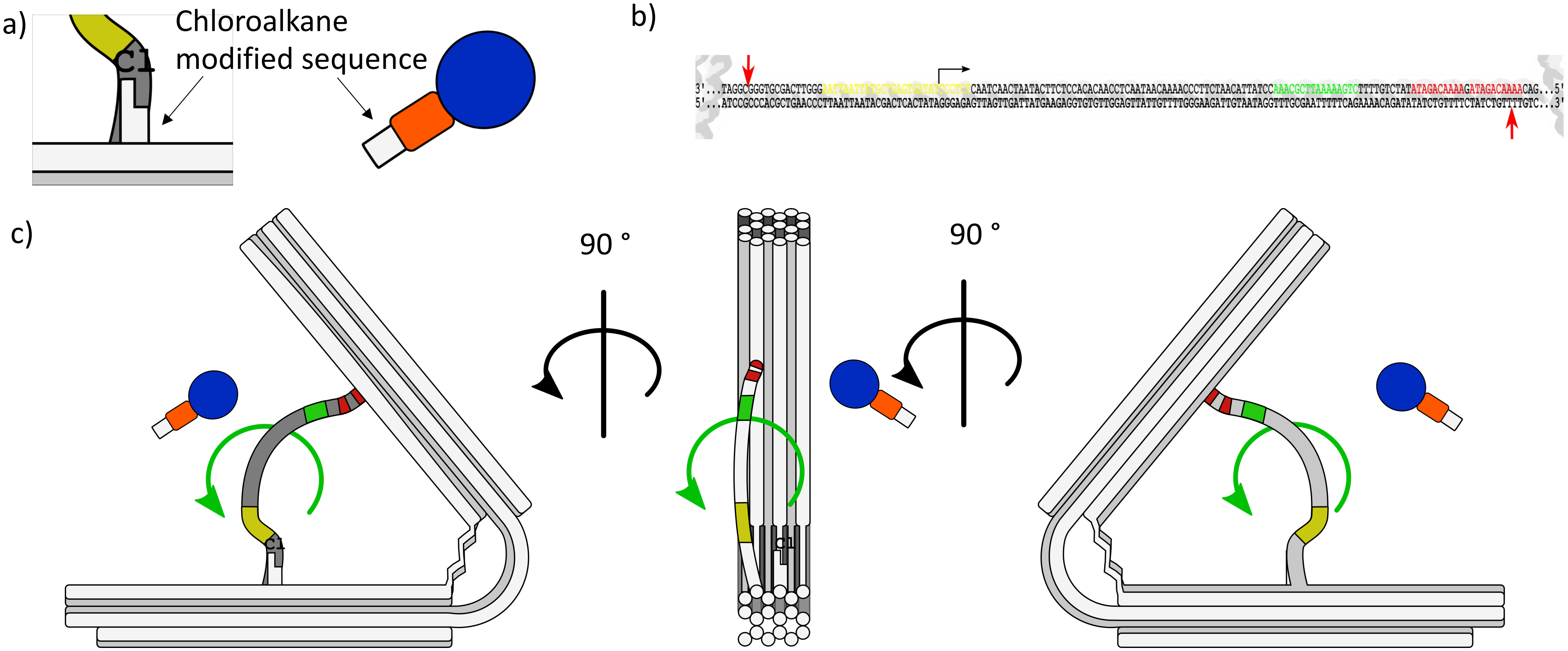

Nicked-nanoengine in presence of the HT-T7RNAP preincubated with 1 equiv of the chloroalkane linker. In this case the HT-T7RNAP was preincubated with the chloroalkane modified ODN. The Enzyme covalently binds to the short ODN and can not bind to the chloroalkane modification on the origami as shown in a). The transcribable dsDNA sequence stays the same as in the nicked-nanoengine b). In c) a graphical representation that shows how the HT-T7RNAP can not bind to the origami and intercats with the origami only intermolecularely.

VII. Nicked-nanoengine + intermolecular chloroalkane linker

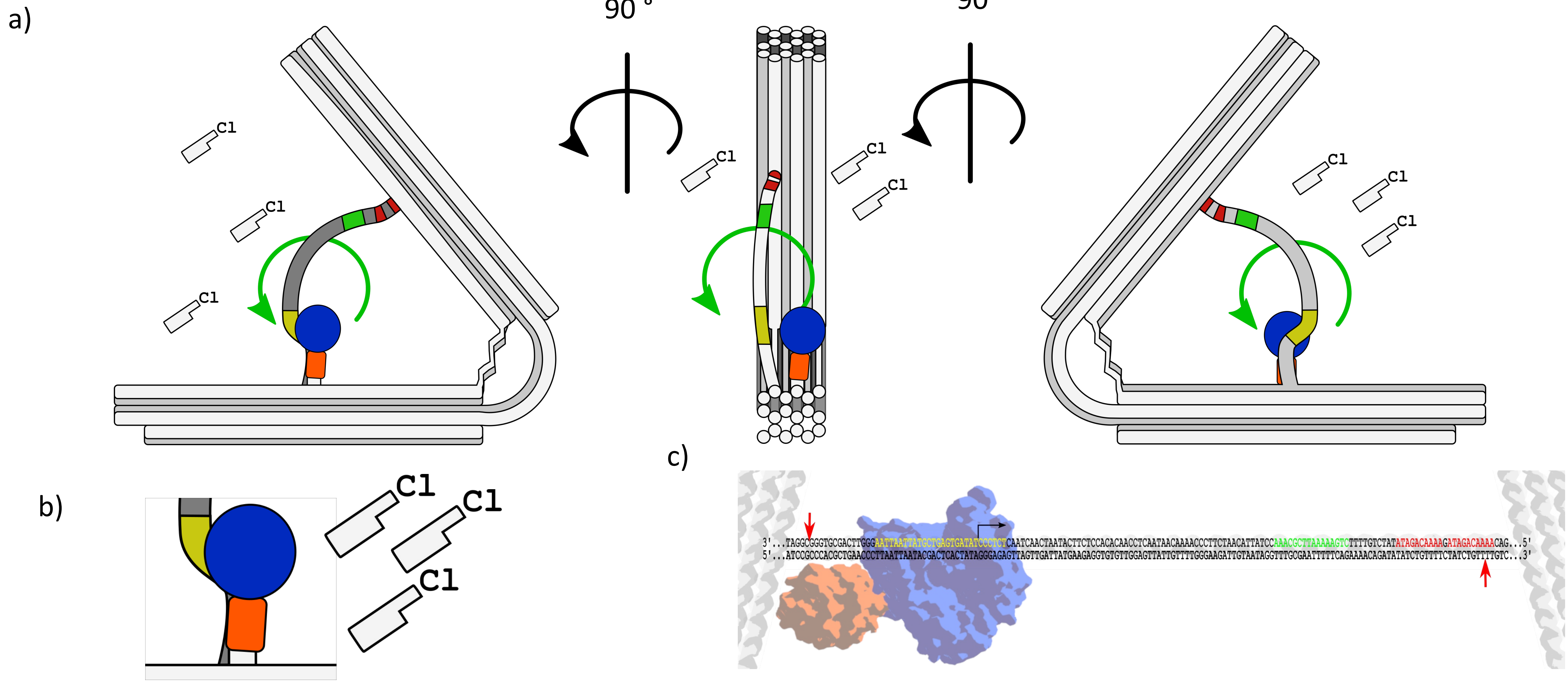

Nicked-nanoengine in presence of intermolecular chloroalkane linker. Once the HT-T7RNAP is covalently bound to the origami, further addition of the chloroalkane modified ODN is not able to displace the polymerase a) with detail in b). The transcribable sequence remains the same as in the nicked-nanoengine in the same configuration as in the nicked-nanoengine in proximity of the covalently bound HT-T7RNAP shown in c).

VIII. Nicked-nanoengine lacking the T7 promoter

a)

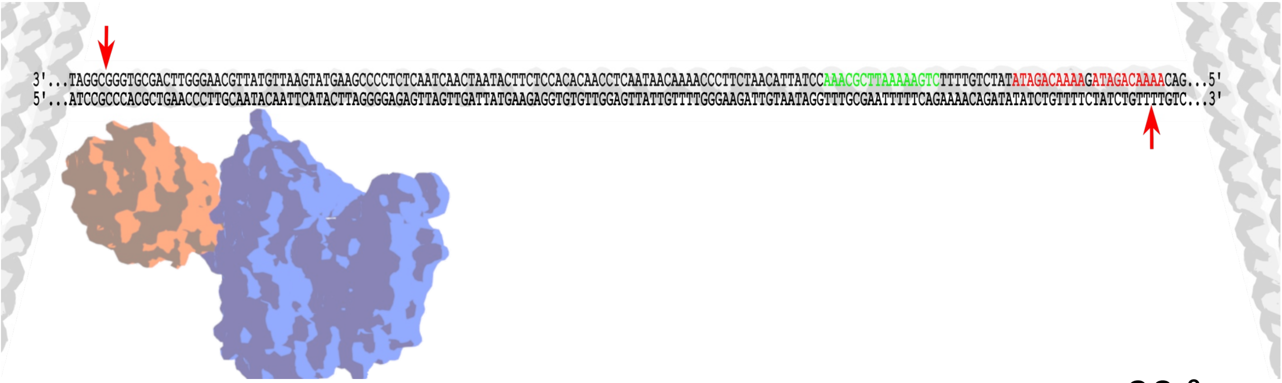

b)

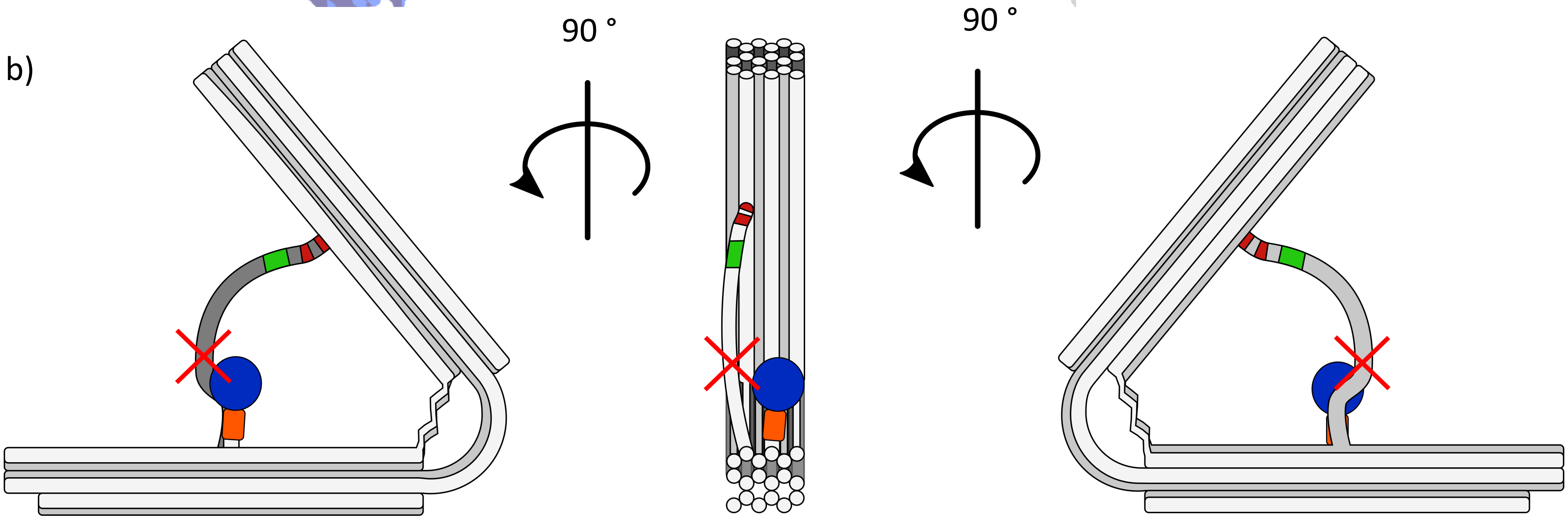

Nicked-nanoengine lacking the promoter region: The promoter region is substituted with a scrambled nucleotide sequence of same length and with same GC content as visible in a). The red X in the schematic representation shown in b) indicate the absence of the promoter sequence.

IX. Nicked-nanoengine with dsDNA-t not connected next to the HT-T7RNAP

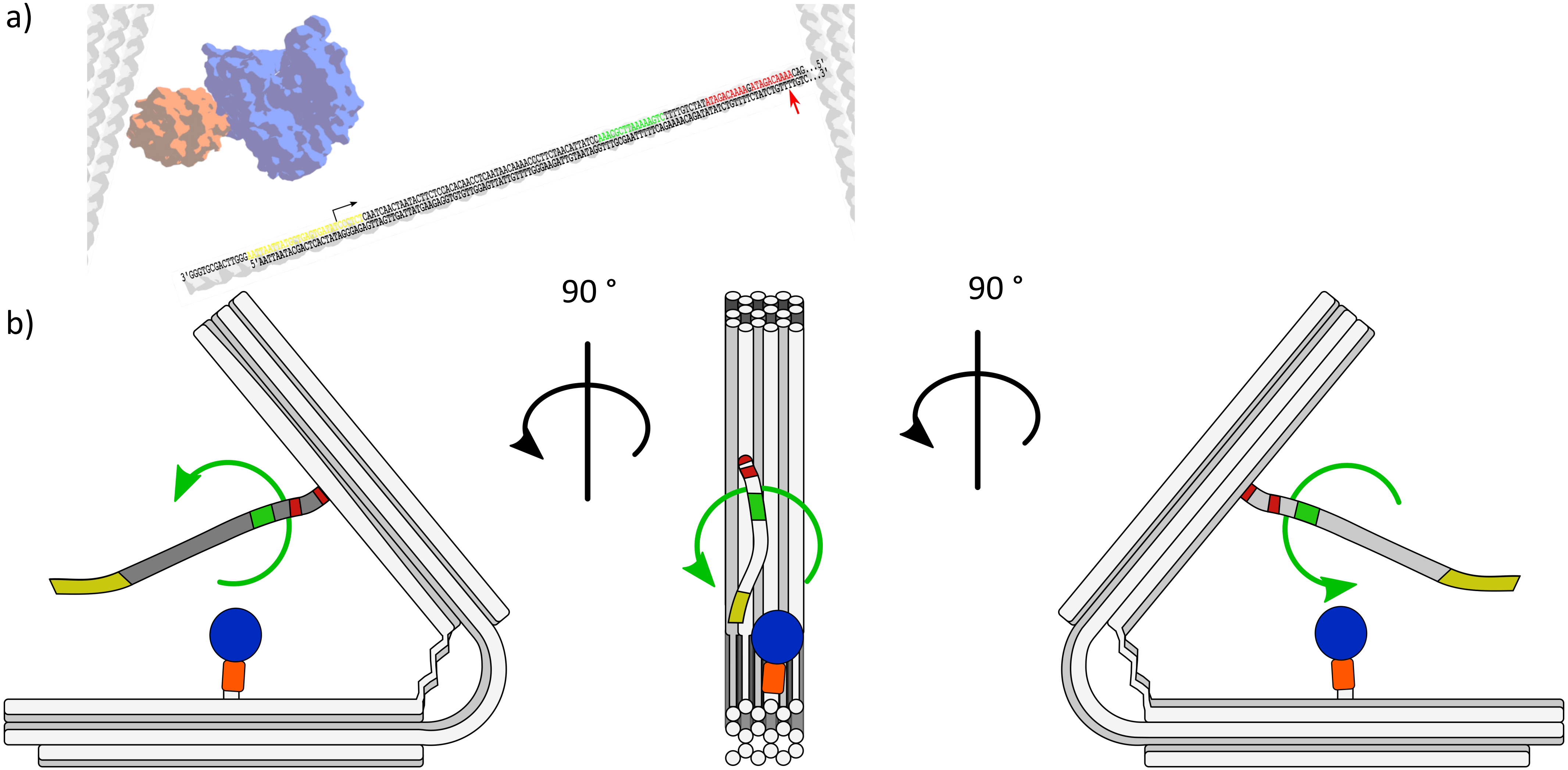

Nicked-nanoengine with dsDNA template not connected next to the HT-T7RNAP. In this structure the transcribable dsDNA sequence is not anchored to the origami next to the polymerase but only on the opposite DNA origami arm as depicted in a). The transcribable sequence has a higher degree of free motion and the promoter is not held close to the polymerase as can be seen from the schematic representation in

X. Nicked-nanoengine with dsDNA-t only connected next to the HT-T7RNAP

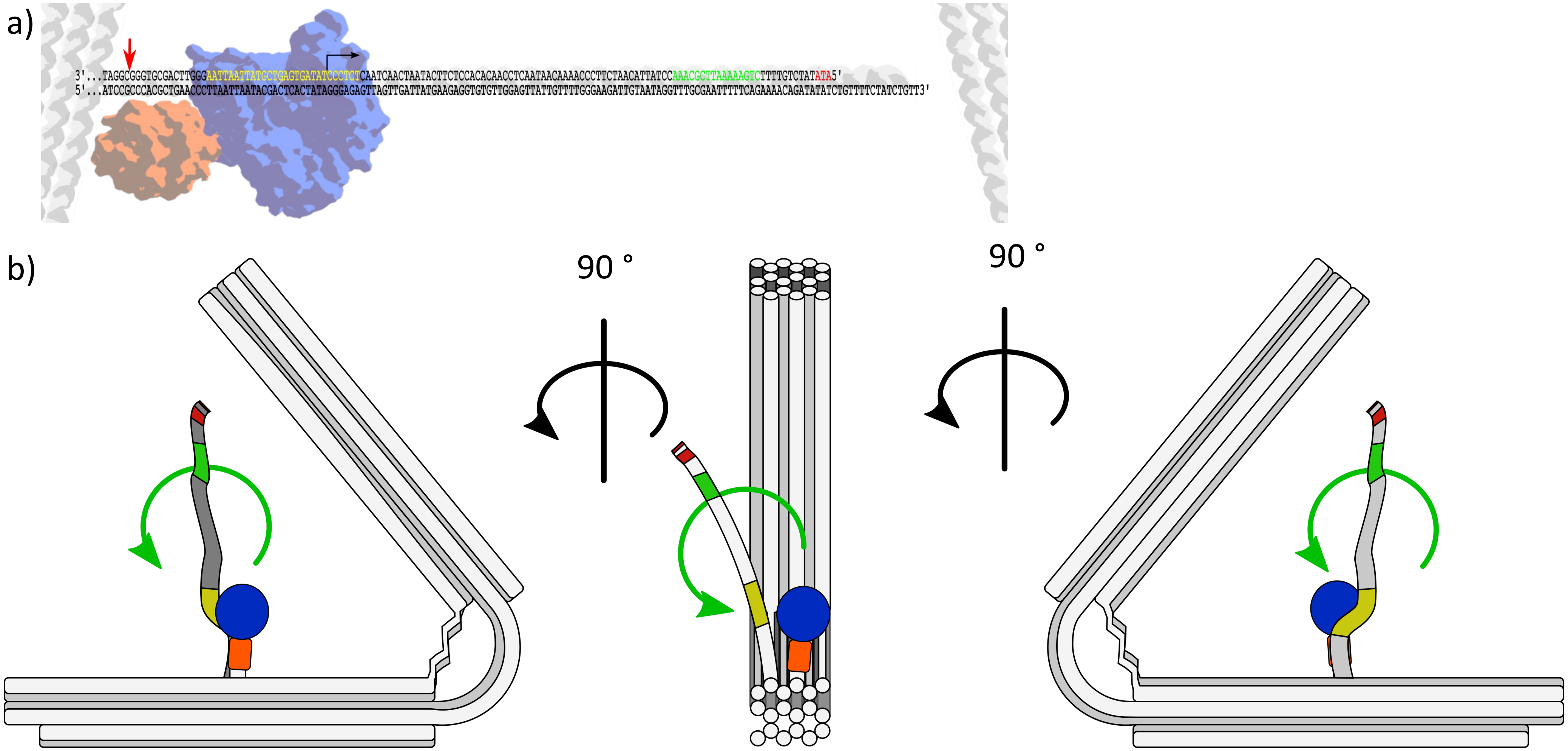

Nicked-nanoengine with dsDNA only connected next to the HT-T7RNAP. The transcribable dsDNA sequence is only anchored to the origami next to the polymerase as can be seen in the detailed depiction in a). The transcribable sequence is held in place close to the polymerase as can be seen in the graphics in b). The transcription in this case does not result in the closing of the origami. The sequence experience a slightly higher degree of freedom compared to the fully connected sequence.
