# Supplementary material for: A rhythmically pulsing leaf-spring DNA-origami nanoengine that drives a passive follower
Source: Nat Nanotechnol. 2023 Oct 19;19(2):226–36. doi: 10.1038/s41565-023-01516-x (PMC10873200; doi:10.1038/s41565-023-01516-x)
Supplement: Supplementary file 9 — Unprocessed AFM scan of Fig. 1f, unprocessed TEM micrograph of Fig. 1g,i,j, unprocessed TEM micrograph of Supplementary Fig. 4b, unprocessed AFM scan of Extended Data Fig. 2a, unprocessed TEM micrograph of Extended Data Fig. 3b [file 41565_2023_1516_MOESM9_ESM.pdf]

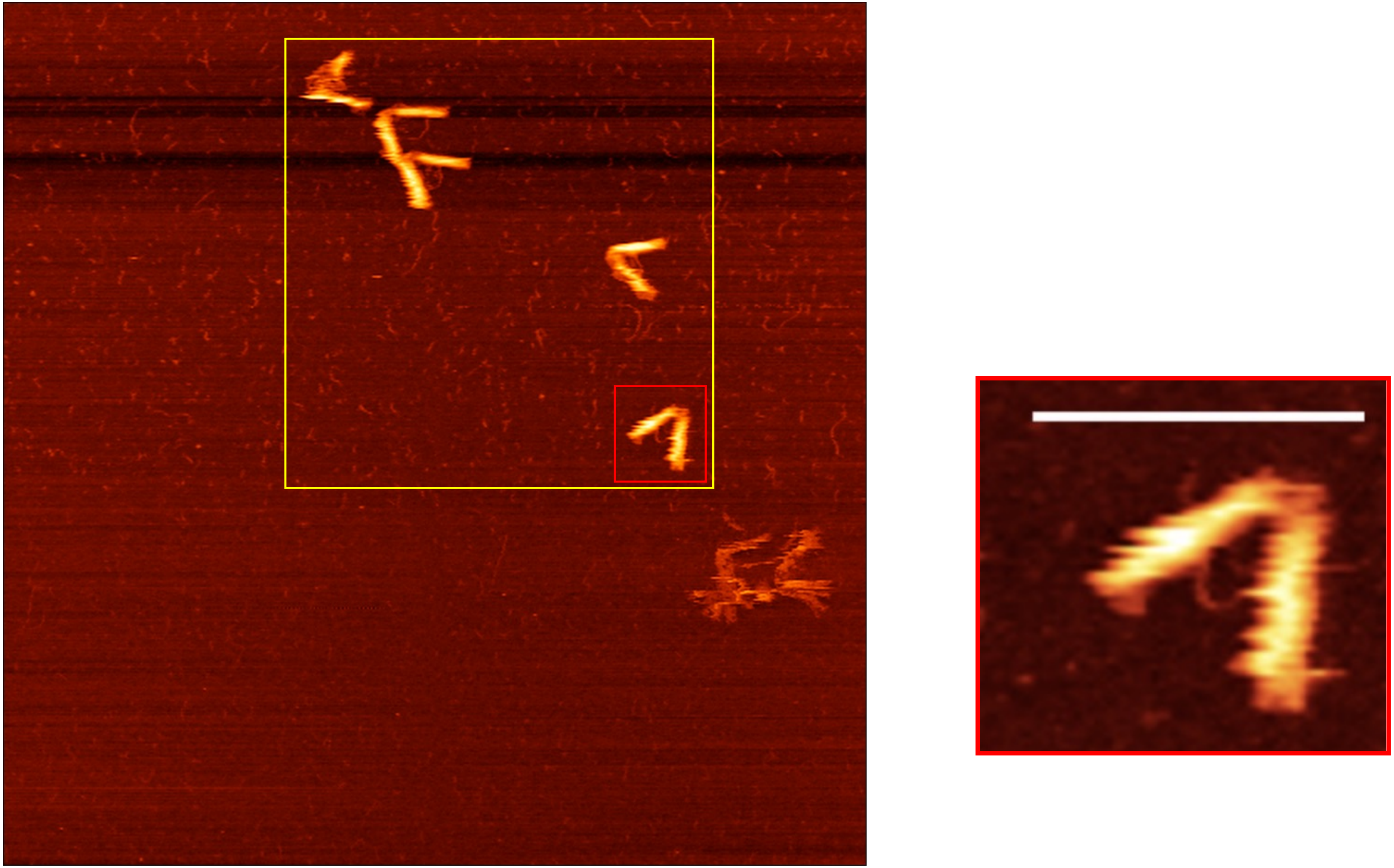

Figure 1f (1) Uncropped AFM scan for Figure 1f (left panel). The yellow framed region is the part shown in the left panel of Figure 1f. The region framed in red is the part shown in the right panel of Figure 2f.

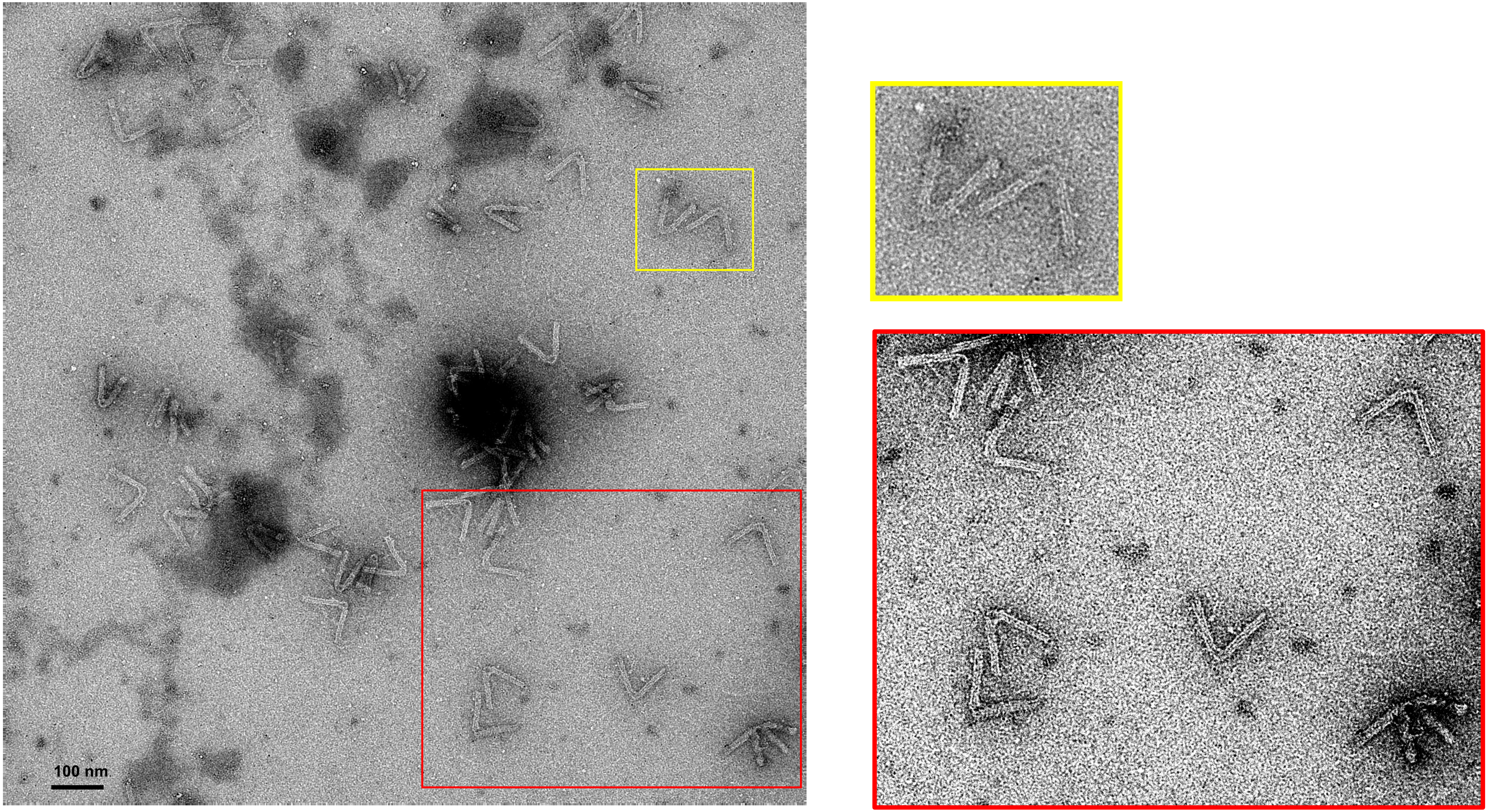

Figure 1g (1) and Suppl. Fig. S4b. Unprocessed TEM micrograph for Figure 1g (left panel) and Suppl. Fig. S4b. The region framed in red is the part shown in the left panel of Figure 1g, the region framed in yellow is the part shown in Suppl. Fig. S4b.

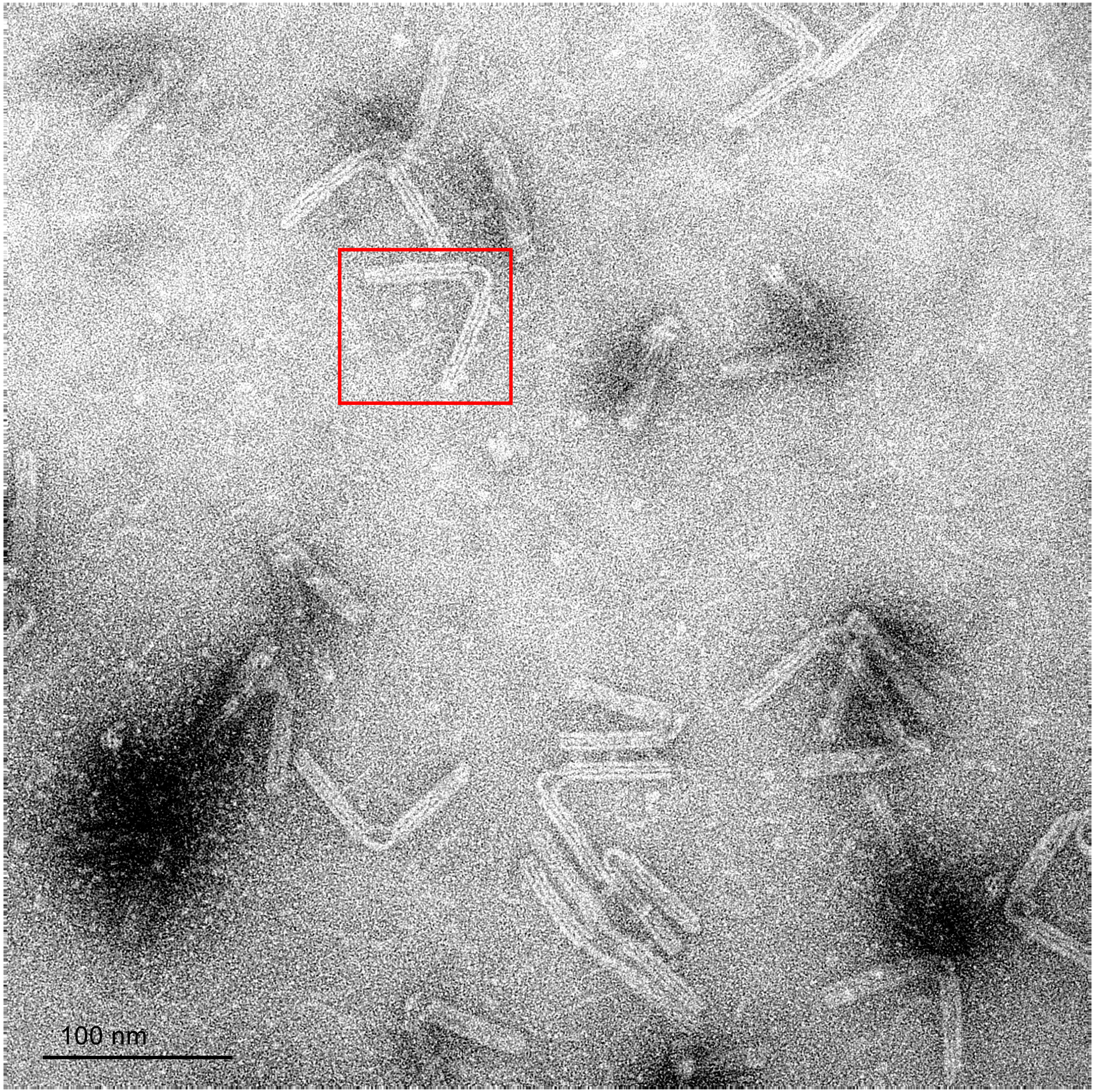

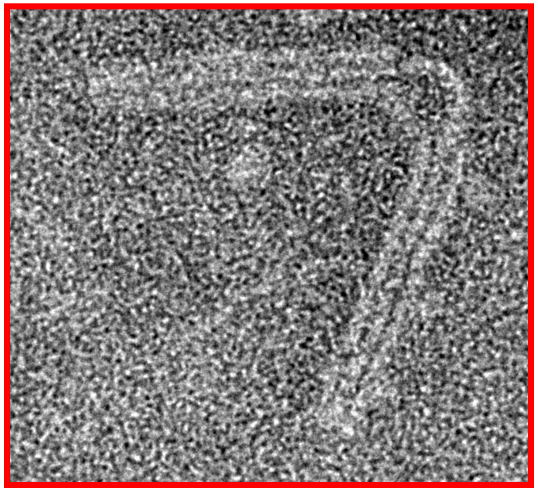

Figure 1 g (2) Unprocessed TEM micrograph for Figure 1g (right panel). The framed region is the part shown in the Figure

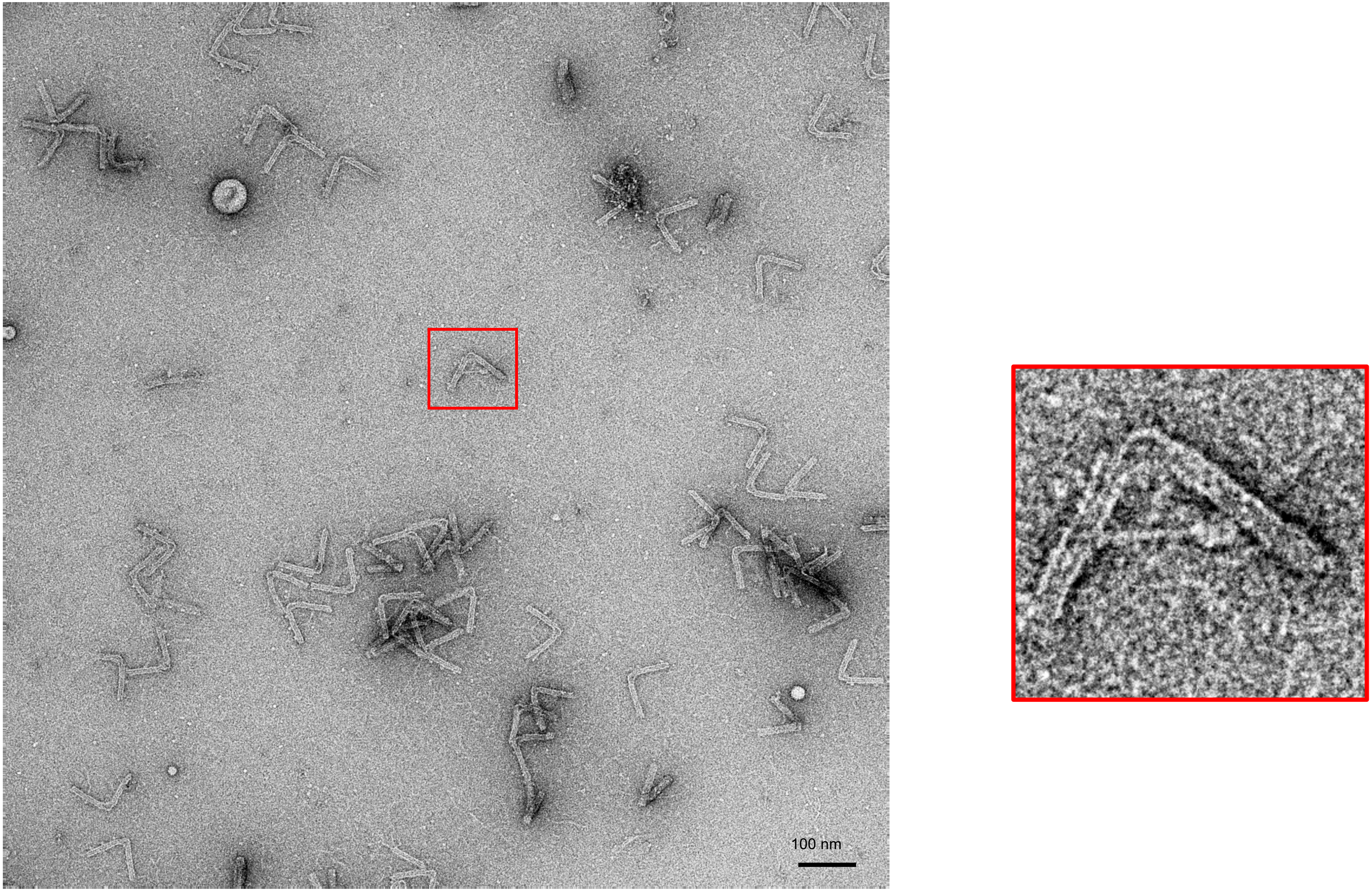

Figure 1i Unprocessed TEM micrograph for Figure 1i. The framed region is the part shown in the Figure

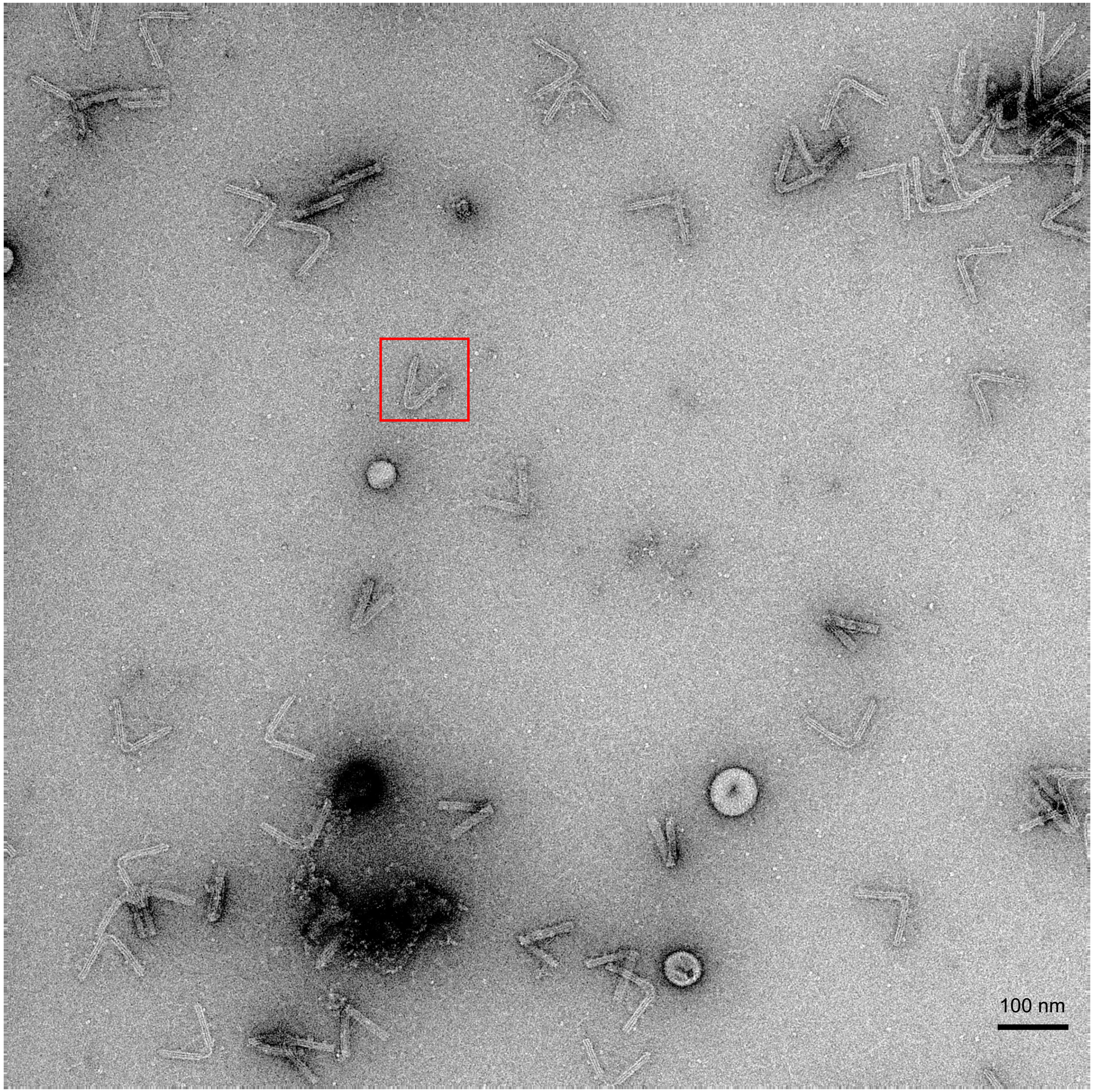

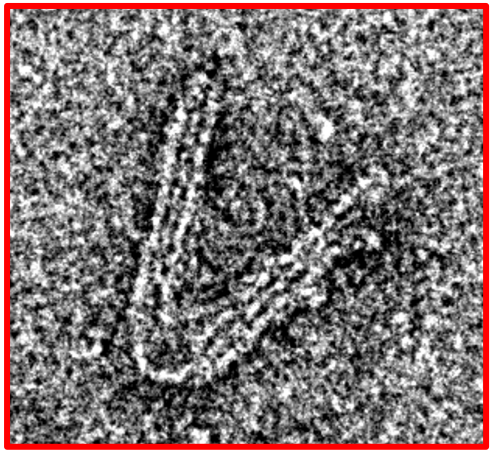

Figure 1j Unprocessed TEM micrograph for Figure 1j. The framed region is the part shown in the Figure

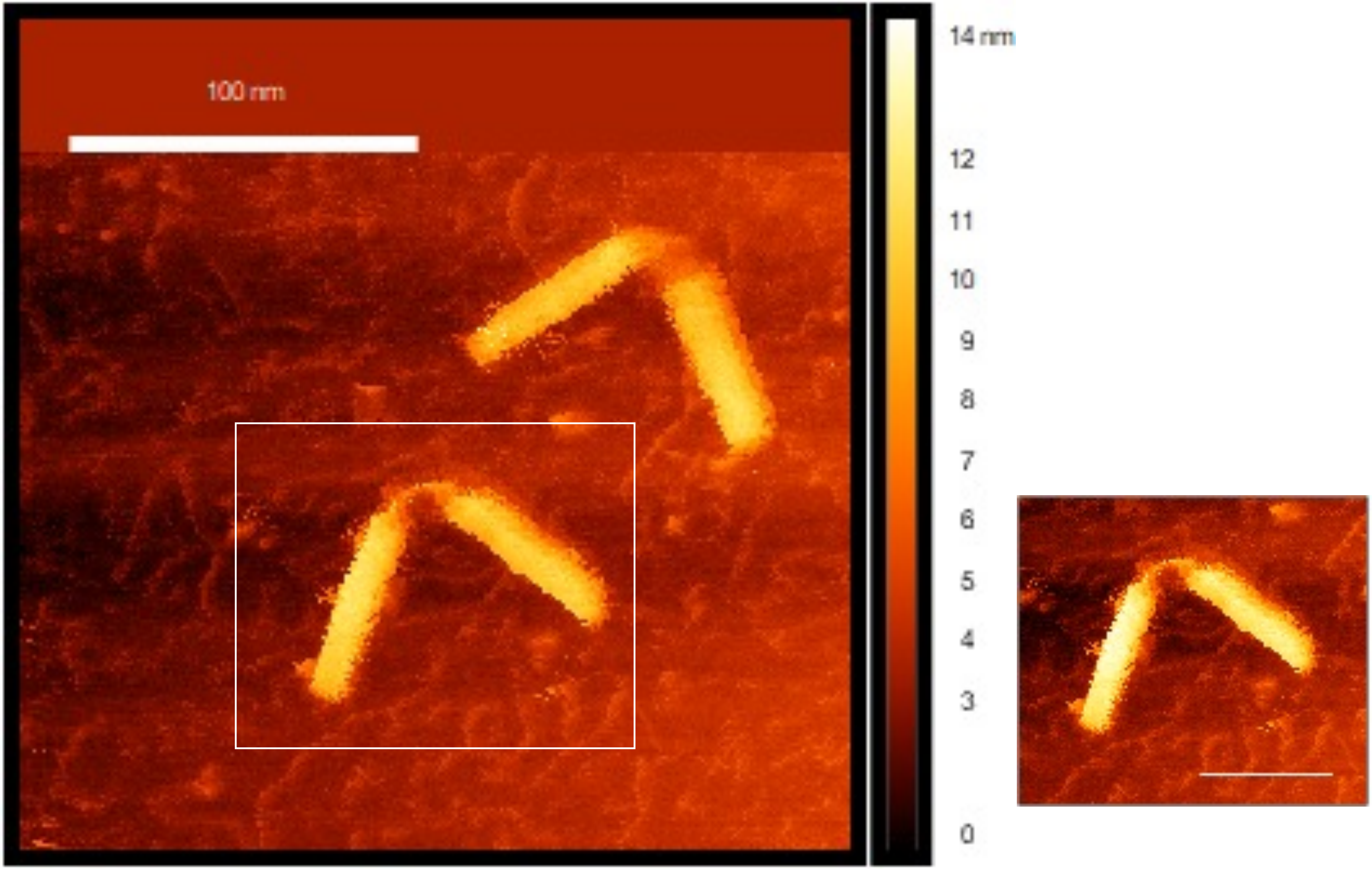

Ext. Data Figure S2a Unprocessed AFM scan for Ext. Data Figure S2a. The framed region is the part shown in the Figure

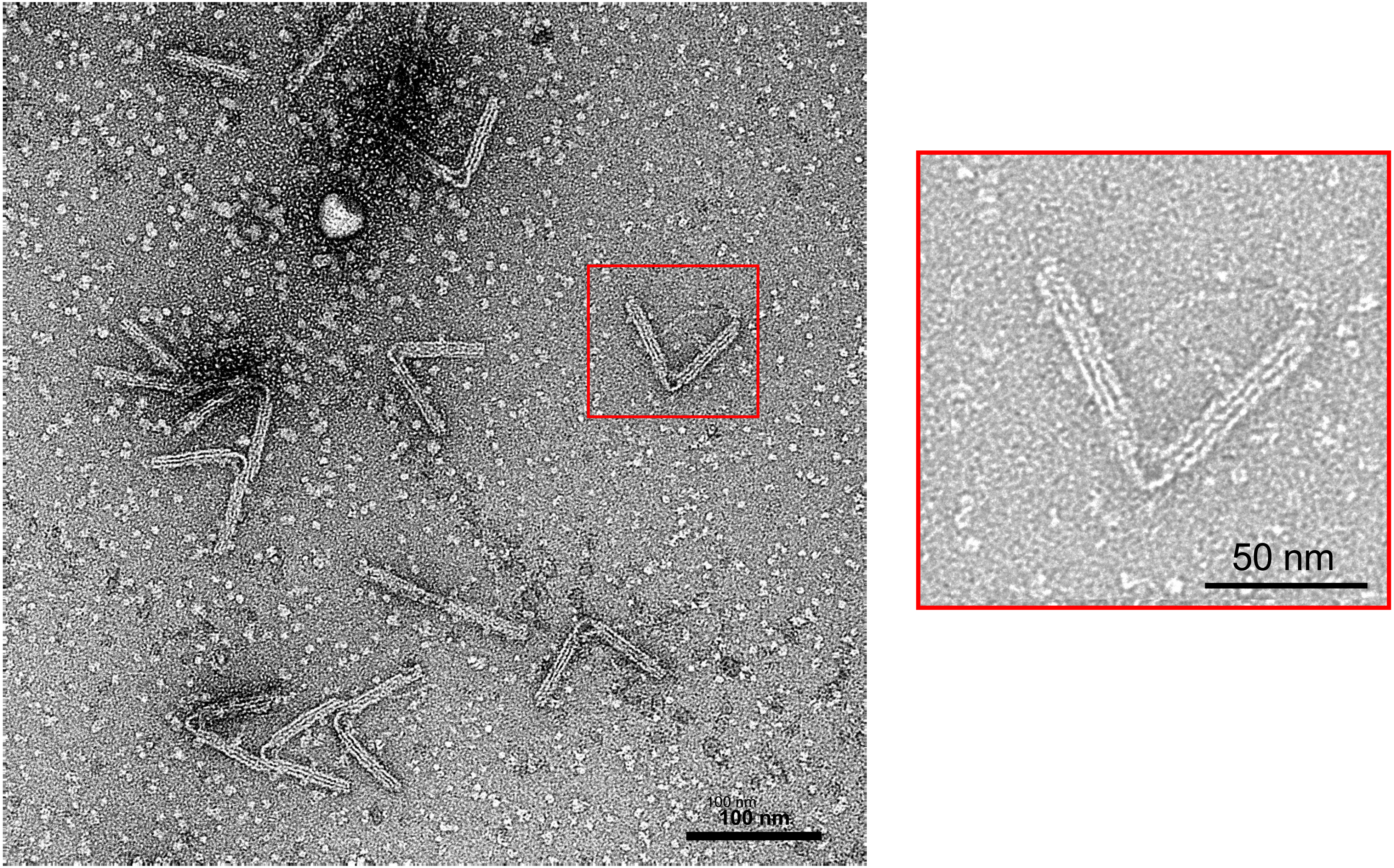

Ext. Data Figure S3b Unprocessed TEM micrograph for Ext. Data Figure S3b. The framed region is the part shown in the Figure
